# Image analysis driven single-cell analytics for systems microbiology

**DOI:** 10.1186/s12918-017-0399-z

**Published:** 2017-04-04

**Authors:** Athanasios D. Balomenos, Panagiotis Tsakanikas, Zafiro Aspridou, Anastasia P. Tampakaki, Konstantinos P. Koutsoumanis, Elias S. Manolakos

**Affiliations:** 1 Department of Informatics and Telecommunications, National and Kapodistrian University of Athens, Ilissia, Greece; 2 Biomedical Research Foundation of the Academy of Athens, 4 Soranou Ephessiou Street, Athens, Greece; 3 Laboratory of Food Microbiology and Hygiene, Department of Food Science and Technology, School of Agriculture, Forestry and Natural Environment, Aristotle University of Thessaloniki, Thessaloniki, Greece; 4 Department of Agricultural Biotechnology, Agricultural University of Athens, Athens, Greece; 5 Northeastern University, Boston, USA; 6 Wyss Institute for Biologically Inspired Engineering, Harvard University, Boston, USA

**Keywords:** Time-lapse microscopy, Machine learning, Bacterial image analysis, Colonies segmentation, Cell segmentation, Lineage tree construction, Visualization, Single-cell informatics, Single-cell analytics

## Abstract

**Background:**

Time-lapse microscopy is an essential tool for capturing and correlating bacterial morphology and gene expression dynamics at single-cell resolution. However state-of-the-art computational methods are limited in terms of the complexity of cell movies that they can analyze and lack of automation. The proposed Bacterial image analysis driven Single Cell Analytics (BaSCA) computational pipeline addresses these limitations thus enabling high throughput systems microbiology.

**Results:**

BaSCA can segment and track multiple bacterial colonies and single-cells, as they grow and divide over time (cell segmentation and lineage tree construction) to give rise to dense communities with thousands of interacting cells in the field of view. It combines advanced image processing and machine learning methods to deliver very accurate bacterial cell segmentation and tracking (F-measure over 95%) even when processing images of imperfect quality with several overcrowded colonies in the field of view. In addition, BaSCA extracts on the fly a plethora of single-cell properties, which get organized into a database summarizing the analysis of the cell movie. We present alternative ways to analyze and visually explore the spatiotemporal evolution of single-cell properties in order to understand trends and epigenetic effects across cell generations. The robustness of BaSCA is demonstrated across different imaging modalities and microscopy types.

**Conclusions:**

BaSCA can be used to analyze accurately and efficiently cell movies both at a high resolution (single-cell level) and at a large scale (communities with many dense colonies) as needed to shed light on e.g. how bacterial community effects and epigenetic information transfer play a role on important phenomena for human health, such as biofilm formation, persisters’ emergence etc. Moreover, it enables studying the role of single-cell stochasticity without losing sight of community effects that may drive it.

**Electronic supplementary material:**

The online version of this article (doi:10.1186/s12918-017-0399-z) contains supplementary material, which is available to authorized users.

## Background

Systems biology is an interdisciplinary field with ultimate goal to elucidate the relationships between molecular states and higher order properties of complex biological systems. Microbial communities are such systems and the study of their collective behavior is a major challenge in the post-genomic era [1–5] in order to identify the sources and role of heterogeneity in the behavior of microbial populations and uncover the mechanisms that lead to specific phenotypes of interest, such as persister cells [6] and biofilms [7]. It has also become clear that deciphering the dynamics of evolving bacterial communities requires multidisciplinary approaches [8, 9]. Microscopy is an important tool that can help us capture data and correlate information at multiple scales, from cell populations to molecules [9]. In particular, time lapse microscopy allows us to monitor the evolution of bacterial communities and generate "cell movies" massively [10]. However, accurate and fully automated image analysis and single-cell analytics methods are required before we can really exploit this abundance of "big data" [9, 10] for systems biology.

It is a fact that we currently know very little on the role single-cell heterogeneity plays in the dynamic behaviour of microbial communities. Technical difficulties hamper the automatic monitoring and tracking of subpopulations and individual cells in growing bacterial colonies at a large scale [9]. Studies on the variability of individual cells behaviour rely on laborious manual annotation of cell movies with only a small number of cells per frame [9–12]. Tracking cells across image frames in overcrowded (dense) bacterial communities with many colonies and thousands of cells in the field of view, extracting automatically single-cell attributes (e.g. size, elongation rate, division time, etc.) and correlating them to molecular and other signatures (e.g. expression of fluorescently tagged proteins) remains elusive. Developing robust and high throughput image analysis pipelines that routinely accomplish these tasks effortlessly will enable single-cell analytics and provide new insights to compelling open questions. It is the combination of accurate single-cell image analysis and single-cell analytics that will empower the development of effective stochastic modeling and systems microbiology approaches. This new capability will allow us to characterize stochasticity in colonial growth dynamics of single-cells [13, 14], model stochastic gene expression in single-cells [15], measure phenotypic variation in bacteria [16], model bacterial state transitions from regular to persister cells [6, 17], or from planktonic to biofilm cells [6].

Bioimage analysis has evolved to become an important discipline in bioinformatics and computer vision [18]. For bacterial image processing, well known open source software packages for analyzing cell movies are the TLM-Tracker [19], CellTracer [20], MicrobeTracker [21] and its successor Oufti [22], and Schnitzcells [23]. The TLM-Tracker [19] uses multiple alternative algorithms for cell segmentation, such as threshold-based, watershed transform and level-set methods. To construct the lineage tree of a colony, it matches overlapping cells in consecutive movie frames. The CellTracer [20] employs the concept of hybrid grey-scale/black-white images and extends image filtering and mathematical morphology operators developed for grey-scale images to work with such hybrid images. This allows it to extract cells iteratively as it gradually converts the original grey-scale image into a binary mask of segmented cells. The MicrobeTracker [21] and its successor Oufti [22] combine several algorithms developed for medical image segmentation and computer vision, including clustering, template-matching, active contours, region growing and level set methods. Schnitzcells [23] can segment cells in either fluorescence or phase contrast images, track cells in a frame-to-frame manner [24] and measure fluorescence.

The main features of the aforementioned state-of-the-art methods are summarized in (Additional file 1: Table S1). Their most important limitations that have motivated our work are the lack of generality and automation. Currently available methods fail quite often to process cell movies acquired using diverge imaging settings (e.g. microscope type, imaging modality etc.) and require time consuming human involvement in a trial-and-error mode in order to produce acceptable results. Moreover, they require extensive parameterization thus becoming unfriendly since users need to become familiar with image processing concepts to make good use of them. For all these reasons, bacterial image analysis has remained a serious bottleneck limiting the complexity of cell movies that can be analyzed efficiently and ultimately the throughput of systems microbiology studies. It is therefore important to develop fully automated computational approaches that can analyze more complex cell movies with many frames, many colonies per frame, many cells per colony, and extract, characterize and track colonies and single-cells successfully, even in movies with imperfect image quality and multiple overcrowded colonies with thousands of cells in the field of view.

Motivated by this need, we present here a complete pipeline that combines image processing and machine learning algorithms to achieve precise bacterial colonies and single-cell segmentation, tracking and phenotypic characterization. Our pipeline, called *BaSCA* (Bacterial Single-Cell Analytics), allows the fully automated segmentation and morphology/expression analysis of individual cells in time-lapse cell movies. We employ a “divide-and-conquer” strategy allowing the independent analysis of different micro-colonies in the input movie. At the colony level, we divide again the problem in order to successively reach down to the single-cells level. This recursive decomposition approach allows us to analyze efficiently colonies regardless of their cell density and deal effectively with dense cell images. To the best of our knowledge, our bacterial image analysis approach is the only one in the field following an aggressive “divide-and-conquer” computation strategy that also facilitates a parallel processing software implementation (work in progress).

Besides its robustness across different imaging modalities and its complete automation (the only information the user has to set is the pixel-to-μm correspondence, the imaging modality, and the type of species imaged), our pipeline supports a high throughput analysis and estimation of a plethora of single-cell properties, a prerequisite for developing a high throughput micro-environment data analytics platform. Moreover, BaSCA offers several unique capabilities: tracking of multiple colonies (that may merge) in the field of view, constructing the lineage tree of each colony, visualizing on the lineage tree the evolution of any desirable single-cell property (e.g. cell length, cell area, cell distance from the colony's centroid, fluorescence intensity etc.), construction of time trajectories of selected single-cell properties (cell property tracks) across image frames etc. All these data analytics capabilities favor high throughput analysis and enable systems biology orientated research both at a higher resolution (i.e. zooming down to the single-cell level) and at a large-scale (observing dense community dynamics). It therefore becomes possible with BaSCA to account for single-cell stochasticity in different phenomena without losing sight of the community effects that may drive it [6, 7, 16, 17].

The rest of the paper is organized as follows. In the Methods section we first describe the time lapse movies and evaluation metrics used to compare BaSCA to other state-of-the-art methods (Materials subsection), and then elaborate on the pipeline of algorithms involved in BaSCA (Methods subsection). In the Results and Discussion section, we present evaluation results with different datasets demonstrating the most important single-cell analytics features of BaSCA and examples of how they can be used in practice. Finally, in the Conclusions section we summarize our findings and point to interesting future research directions.

## Methods

### Materials

#### Datasets

The following datasets were used in the evaluation of this work:

##### SalPhase

A time lapse movie acquired by phase-contrast optical microscopy, monitoring four single cells of *Salmonella enterica* serotype Typhimurium that divide to become three discrete micro-colonies (86 frames in total, 5 min sampling period, 1360x1024 pixels resolution, see [13] for more details). From now on, we will refer to this movie as "SalPhase" and going from top left to bottom right we will refer to the three colonies encountered as colony 1, 2 and 3 respectively. This dataset is provided as Additional file 2.



**Additional file 2:** SalPhase time-lapse movie. (MP4 2644 kb)


##### Multi-SalPhase

This time lapse phase-contrast optical microscopy movie includes multiple growing micro-colonies of *S.* Typhimurium (101 frames, 5 min sampling period, 1360x1024 pixels resolution, see [13] for more details). This more complex movie contains overcrowded merging colonies and the total number of cells exceeds three thousands in late frames. This dataset is provided as Additional file 3.



**Additional file 3:** Multi-SalPhase time-lapse movie. (MP4 4064 kb)


##### Individual frames

We have also analyzed several image frames of different imaging modalities generated by different laboratories that are publically available. MicrobeTracker’s frame is a phase-contrast CSLM image (1344x1024 pixels resolution) of sparse *Escherichia coli* cells and micro-colonies available at MicrobeTracker’s webpage [25]. CellTracer’s frame contains a micro-colony of *E. coli* acquired by phase-contrast optical microscopy (901x689 pixels resolution) and was derived from a movie produced in [26] and provided at CellTracer’s webpage [27]. TLM-Tracker’s image contains a micro-colony of *Bacillus megaterium* acquired by bright-field optical microscopy (1300x1030 pixels resolution) generated in [28] and available at TLM-Tracker’s webpage [29]. Finally, Schnitzcells’ frame contains a micro-colony of *E. coli* acquired by phase-contrast optical microscopy (472x538 pixels resolution) and was derived from a movie provided at the Schnitzcells’ webpage [30]. Let us mention that Schnitcells can handle only uint16 format images, thus each of the aforementioned datasets was converted to this format too so as to enable comparative evaluation of Schnitchells with the rest of the methods.

#### Evaluation metrics

Comparative evaluation was performed based on metrics commonly used in pattern recognition, such as true positives (TP), i.e. actual cells that were correctly classified as cells, false positives (FP), i.e. artifacts that were incorrectly classified as cells, and false negatives (FN), i.e. actual cells that were missed. We remark that artifacts can be either due to noise or fragments of over-segmented cells. Furthermore, for each method we computed the *True Positive Rate* (TPR) or recall

*TPR* = *TP*/(*TP* + *FN*),

which represents the percentage of the true cells found, the *Positive Predictive Value* (PPV) or precision

*PPV* = *TP*/(*TP* + *FP*),

which represents the probability that a detected cell is a true cell. The former metric is used to characterize the sensitivity and the latter the specificity of a method. Additionally, we computed the *F-measure* [31], i.e. the harmonic mean of *TPR* and *PPV* that is commonly used to assess the recall versus precision trade-off:

*F* = 2 ⋅ (*PPV* ⋅ *TPR*)/(*PPV* + *TPR*).

### Methods

The developed BaSCA analysis pipeline consists of five stages: image preprocessing, bacterial colonies segmentation, single-cells segmentation, cells tracking and lineage trees construction, single-cell attributes estimation and visualization. Specifically, we formed a computational pipeline, focusing on detecting, segmenting and characterizing each colony and individual cell in the movie. Initially, we process the whole image and extract individual colonies. Then we analyze each colony into a partition of “objects” containing one or more cells. Gradually, we zoom in and reach the desired result; accurate single-cell boundaries detection and cell features estimation. Below, we describe each stage of the developed pipeline in detail.

#### Image preprocessing and colonies segmentation

**Fig. 1:**
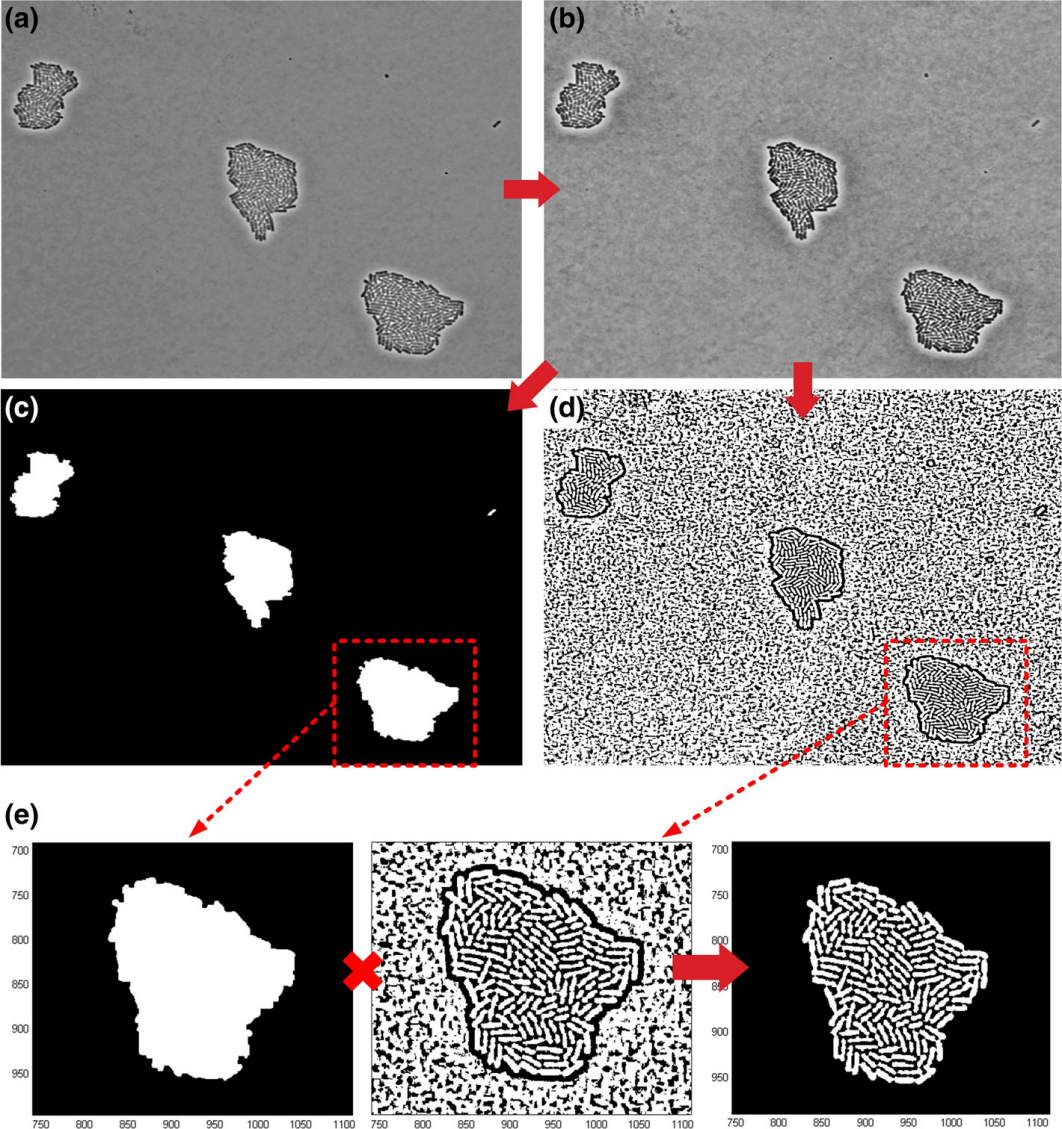
Image preprocessing and Colonies Segmentation. **a** Input image with three colonies (SalPhase movie). **b** Contourlet based denoising and adaptive histogram equalization used to sharpen cell edges. **c** Colony masks created using morphological filtering, Otsu’s global thresholding and Canny edge detection; they are used to separate colony regions from image background so that each colony can be processed separately in the pipeline (divide-and-conquer). **d** Adaptive thresholding is used to remove the colony’s local background pixels. **e** Multiplication of the image generated by adaptive thresholding with the corresponding extracted mask (Colony in the red rectangle) removes the noise (existing locally in the colony) and the artifacts (produced by the adaptive thresholding algorithm) while revealing cell objects inside the colony

Image preprocessing is a necessary initial step to suppress noise and correct for image background abnormalities (see Fig. 1(a-b)). We apply first Contourlet Transform based image denoising, as described in [32–35]. Then, we use Contrast-Limited Adaptive Histogram Equalization (CLAHE) [36] to enhance cell regions in the image and suppress any luminous local micro-colonies background that exists in some imaging modalities (see Fig. 1(b)). As a result, we manage to remove noise and at the same time separate the background from information bearing cell regions.

After preprocessing, our colony segmentation method creates a binary mask used to separate each colony from the image background. We first apply a mathematical morphology operation (rolling ball method) [37] to estimate the image background. Then we use Otsu’s global thresholding [38] and Canny’s edge detection algorithms [39] to create a binary image (global background mask) outlining as precisely as possible the region of each colony, regardless of its size (see Fig. 1(c)).

After completing this task for each image frame, we track each colony and extract its properties (e.g. number of cells, total area, location of the colony’s centroid etc.) in the time-lapse movie. Specifically, in order to determine the correspondence of two colonies in two consecutive frames, we check whether the centroid of a colony in the previous frame lies inside the bounding box of a colony in the current frame, and if so this colony is matched. If a colony in the current frame matches to no colony in the previous frame, we treat it as a new one, since we consider it possible for a colony to enter to the microscope’s field of view while the experiment is running. When two or more colony centroids of the previous frame lie inside the bounding box of the same colony in the current frame, the algorithm identifies that these colonies have merged. This capability is important since in a large bacterial community colonies can merge or move out of the field of view. Keeping track of colonies as they grow and merge is important not only for archiving their time varying properties but also for knowing the colony from which each individual cell has emerged i.e. the subpopulation to which it belongs to, without using any fluorescent markers. To the best of our knowledge, our divide and conquer computational approach is the only one that can track multiple subpopulations (colonies) that may merge, in addition to single-cells, in cell movies.

#### Single-cell segmentation algorithm

**Fig. 2:**
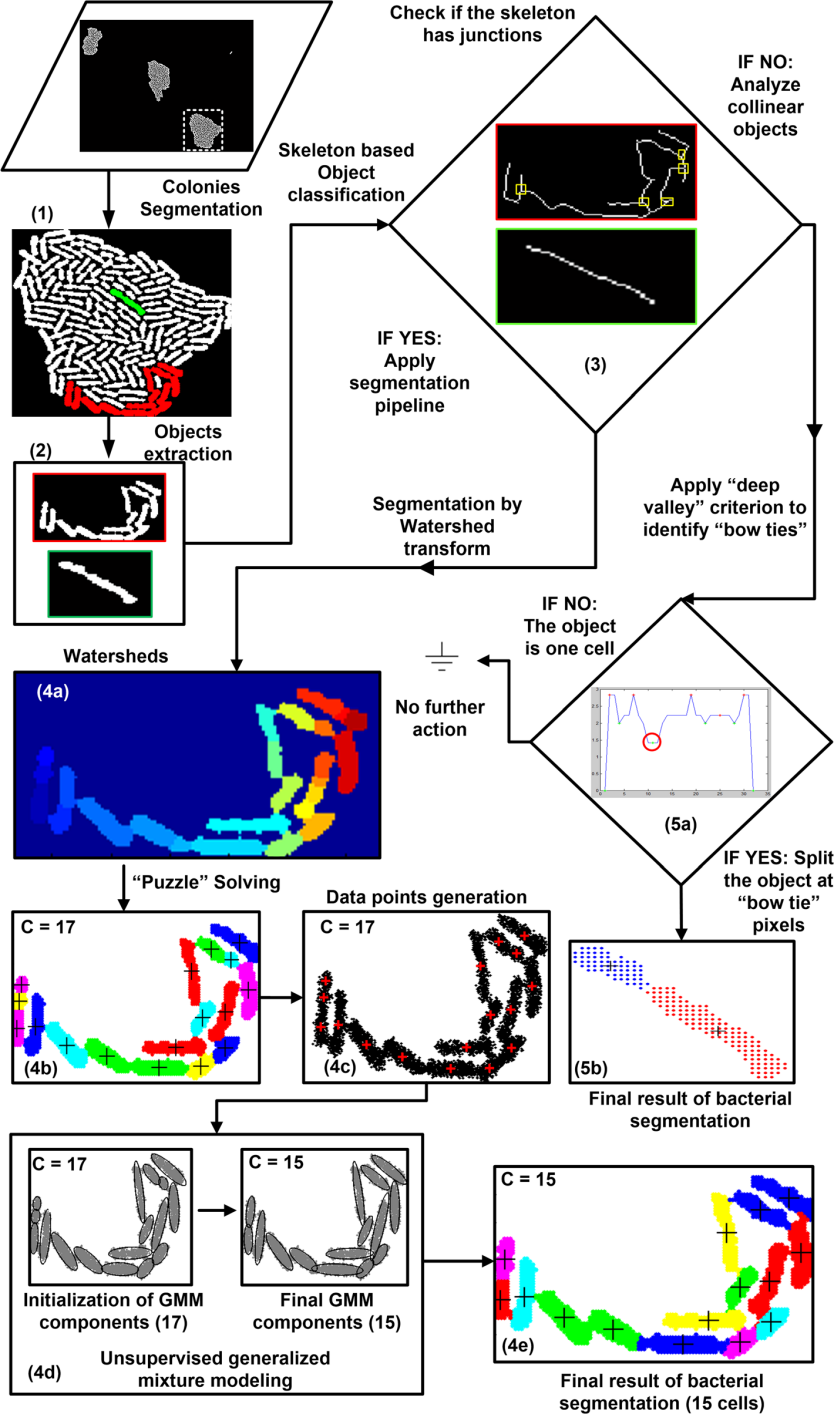
Overview of the cells segmentation method. (1) Colonies segmentation. (2) Objects identification inside a colony. (3) Skeleton based object classification; (4) *Complex object segmentation:* 4a) Watershed algorithm application, 4b) “puzzle solving” step, 4c) new dataset generation, 4d) unsupervised Gaussian mixture modeling, 4e) final result of bacterial cells segmentation. (5) *Collinear object segmentation:* 5a) Application of the “deep valley" criterion to identify “bow tie” points, 5b) final result of bacterial segmentation. At step (3), there is a bifurcation leading to different processing routes based on the object's classification. See text for details

Having defined a colony’s region as accurately as possible, we can now “zoom in” and detect individual cells effectively. A visual overview of the whole segmentation approach is provided in Fig. 2.

##### From colonies to cell objects

Despite the applied preprocessing, each colony may still contain local background pixels due to large illumination variations (e.g. see Fig. 1(b)). To overcome this problem, we use adaptive thresholding [37] to separate cells from the non-uniformly illuminated background (see Fig. 1(d)). We remark that this type of background variability is typical for images acquired by optical microscopes (either bright field or phase contrast) but not for images acquired using confocal laser scanning microscopes (CLSM) [40]. Nevertheless, the adaptive thresholding method used here has no negative effect on CLSM images. However, as illustrated in Fig. 1(d) adaptive thresholding introduces "salt and pepper" noise, so in order to eliminate it we multiply the processed image with the global background mask. The result is an image with the global background removed and the local noise inside each colony suppressed (see Fig. 1(e) for the 3rd colony of SalPhase movie).

**Fig. 3:**
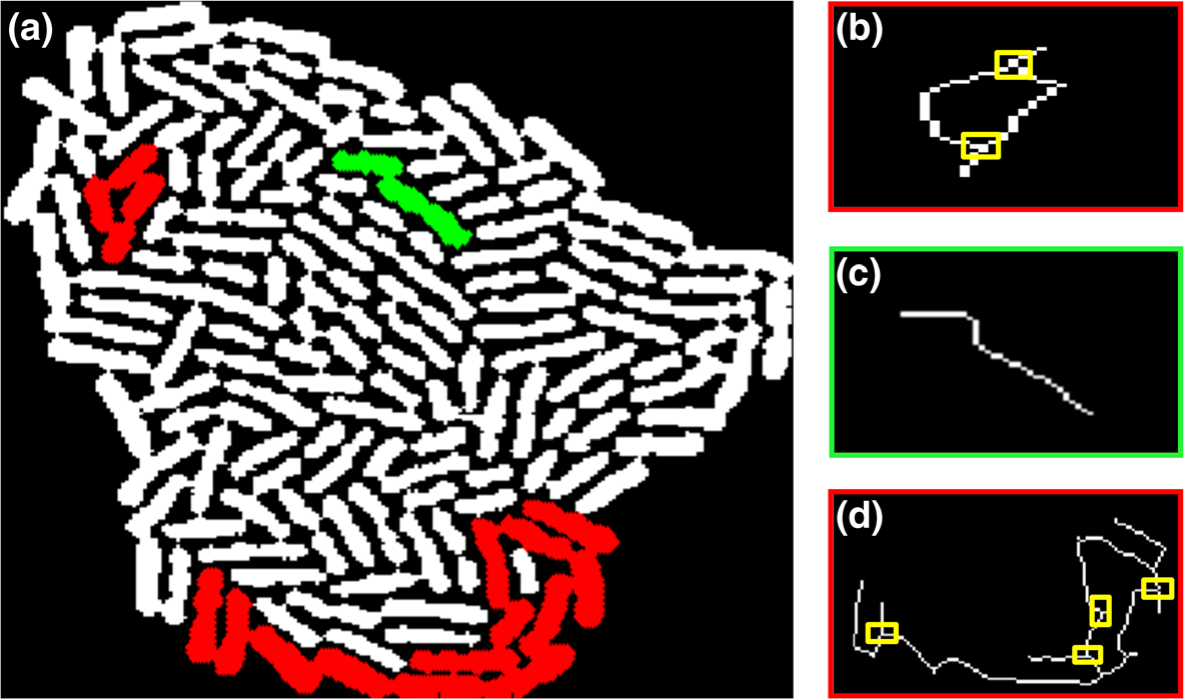
Cell Objects extraction and classification. **a** Each colony is an ensemble of cell objects corresponding to one or more “touching” cells; e.g. see three cell objects marked with color. We first extract the skeleton of each object and classify it as *complex* (*red*) or *collinear* (*green*) according to the presence (or absence) of skeleton junctions (i.e. skeleton pixels with more than two neighbors). **b** The skeleton of the smaller *red object* has two junctions (marked with *yellow boxes*) so it is classified as a *complex* object. **c** Colony object with no junctions (*green*) classified as *collinea*r object. **d** A complex object (*large red*) with four junctions. See text for details

However, the individual cells inside the colony may not be fully separated (segmented) at this stage. We rather observe that the colony is partitioned into "cell objects", where each object is a set of cells "touching" each other (see Fig. 3(a) for examples). The union of these objects covers all cells in the colony and their pairwise intersection is empty (every single-cell belongs to one and only one object). In the colony of Fig. 3 (a) three objects have been colored for demonstration purposes, one green and two red. The green is a so called **collinear** object while the two red ones are considered **complex** objects. To determine an object’s category (collinear vs. complex) we developed a divide-and-conquer approach where we analyze each object individually. First, we compute the object's skeleton as presented in [37] (refer to Fig. 3 (b-d)). Then, if the skeleton has "junction points" (i.e. pixels that have more than two pixel neighbors [37]) the object is considered complex. On the other hand, if we find no junction points, the object is either a single-cell or a cascade of single-cells (collinear object), see Fig. 3 (c).

##### Collinear object analysis

**Fig. 4:**
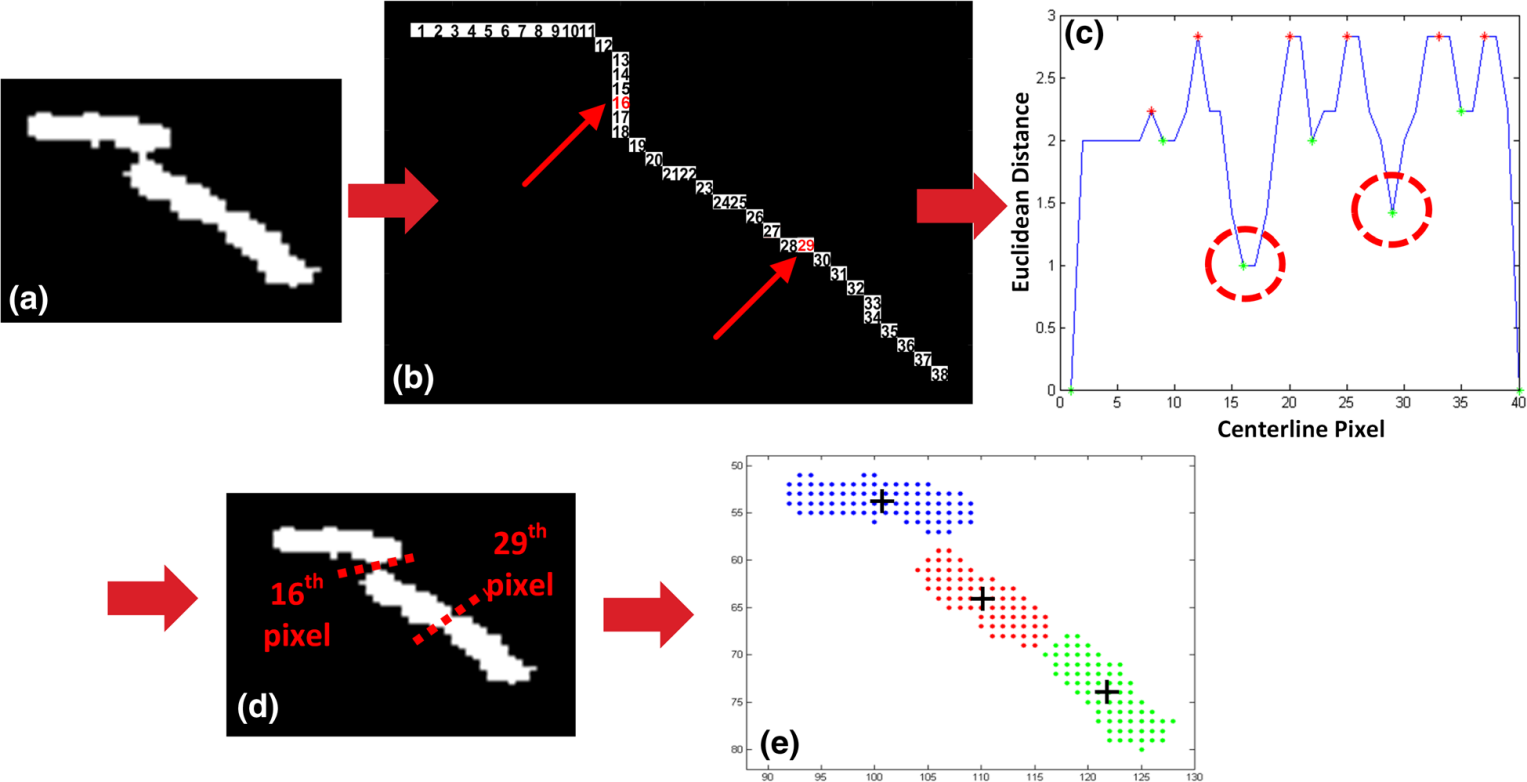
Collinear objects analysis - "Bow ties" identification. **a** Extracted collinear object. **b** Skeletonized object with skeleton pixels numbered. The *red numbers* mark the “bow tie" locations on the centerline. **c** Distance curve of the object: To construct it we form pairs of opposite-side diametric boundary pixels (w.r.t. the skeleton) and compute their Euclidean distance (local width). Then we search for “deep valleys” (i.e. significant local minima relative to neighboring local maxima (marked with *red circles* for illustration purposes) and, (**d**) we split the object at bowtie points (marked by *red dashed lines* for illustration purposes), that correspond to the deep valley positions in (**c**). **e** The collinear object is segmented into three single-cells

In order to ascertain if a collinear object corresponds to a single-cell or a series of connected single-cells (i.e. a cell "sausage" like structure) we developed a simple algorithm based on the observation that the shape of a bacterium at the fission stage [41] resembles that of a bow tie (refer to Fig. 4 (a)). The algorithm searches for "bow tie points" by computing the Euclidean distances [42] of all pairs of diametric pixels (antipodal) lying on opposite boundaries with respect to the object’s centerline. Then, it identifies significant local minima (Fig. 4 (c)) of the distance curve, meeting the following criteria:

*localMin*/*leftLocalMax* ≤ *T* and *localMin*/*rightLocalMax* ≤ *T*

where *localMin* is the local minimum and *leftLocalMax* and *rightLocalMax* are the local maxima lying around the local minimum. Such local minima (if they exist) are called “deep valleys” (Fig. 4 (c), indicated by red circles). Intuitively, *T* is the ratio of the minimum to maximum width (i.e. the distance of diametric pixels with respect to the object’s centerline from the center of the one pole semi-circle to the center the other pole semi-circle) measured at the frame before the first cell division. The value of threshold *T* is automatically set by the pipeline and is usually in the range [0.65, 0.75]. When the algorithm identifies the existence of deep valleys, we split the object into discrete cells at these locations (marked with red dashed lines in Fig. 4(d)), that correspond to the deep valley points on the centerline (the x-axis in Fig. 4(c)) because the centerline pixels are ordered (see Fig. 4(b)). Otherwise the collinear object remains intact and is considered as a single-cell. Finally, for each segmented cell, we identify its centroid by averaging its pixel coordinates (see Fig. 4(e)).

##### Complex object analysis

Complex objects are treated differently than collinear objects. First we use the watershed transform [43] to estimate how many cells are possibly "hidden” inside a complex object. We determine the centroids of extracted watersheds and consider them as initial estimates for the centroids of potential cells in the complex object (see Fig. 2 (4a)). However, a well-known problem of the watershed transform is that it favors over-segmentation [44]. So, we apply the “deep valleys” algorithm again, but in a slightly modified way. This time we need to decide whether we should merge erroneously over-segmented cell fragments, so as to avoid generating false positive cells. Fragments in the neighborhood of a given fragment are examined to determine if they should be merged with it or not. Two touching fragments can be merged if there is no bowtie point between them, identified using the deep valleys criterion. If a potential cell (watershed fragment) can be merged with more than one neighboring cells, then the merging that leads to maximum solidity is chosen. Solidity is defined as the ratio of the resulting area to the resulting convex hull area, based on the conjecture that elementary objects, i.e. well-formed single-cells, tend to have a solidity value close to one. Moreover, the new object (after the merging) is inserted into a processing queue in order to be further examined whether it should be merged with another watershed fragment. We call this iterative procedure “puzzle solving” algorithm (see Fig. 2(4b)).

**Fig. 5:**
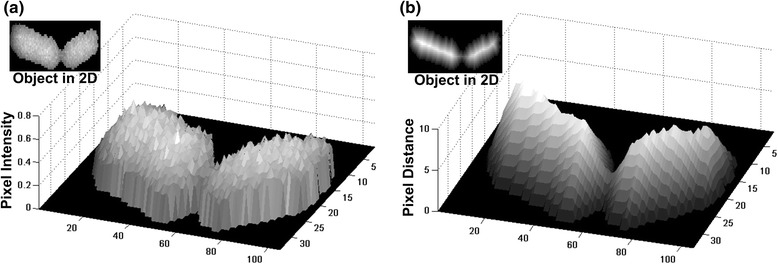
Distance transform. **a** Cell object depicted in 3D; the image pixel intensities are shown in the z-axis. **b** Same object's distance transform in 3D; the distance values are shown in the z-axis. It is obvious that the distance transform [40] smoothens object abnormalities while sharpening the valley between the two cells comprising the cell object

**Fig. 6:**
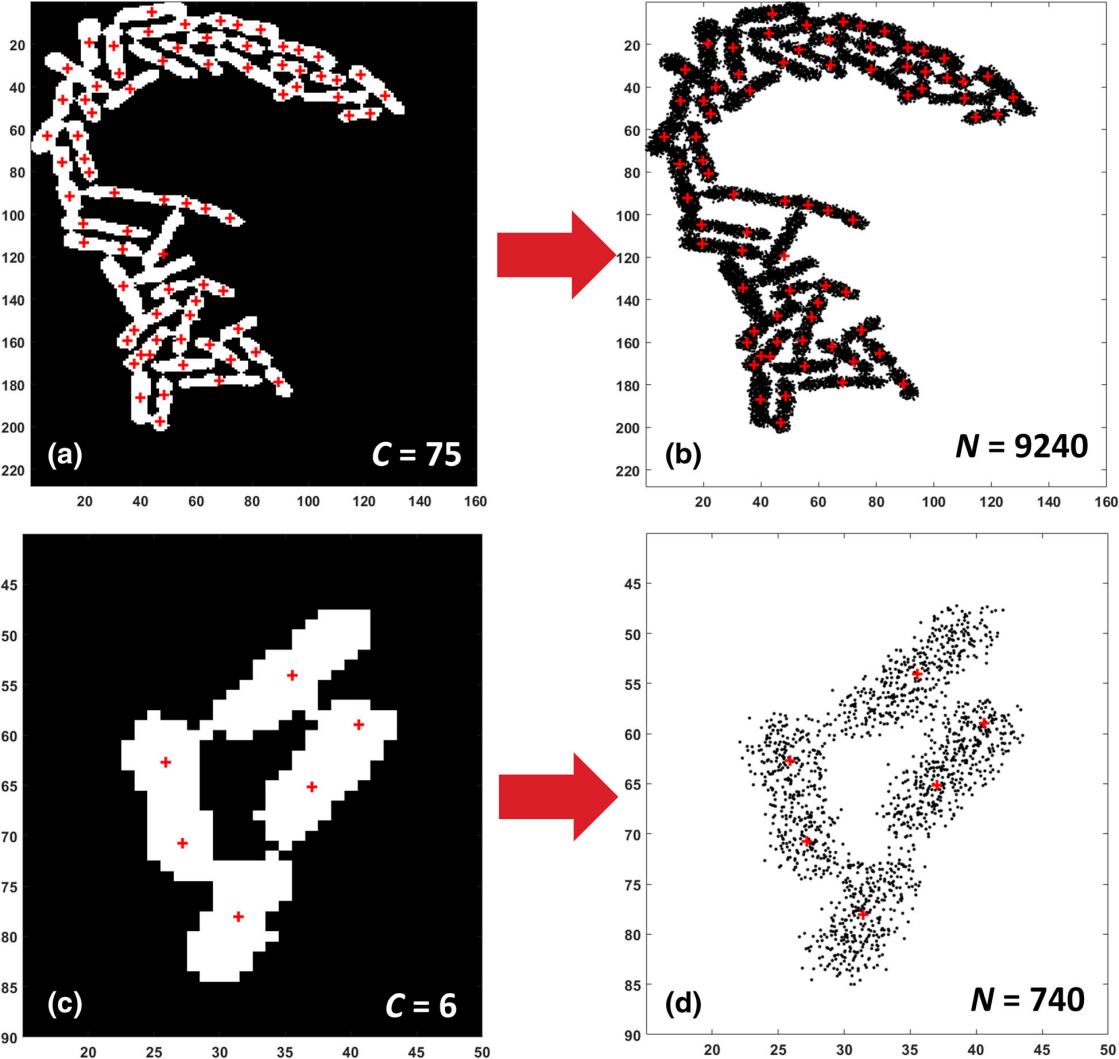
Complex object Analysis - Generation of a dataset representing the object. **a** A very complex object (large number of cells) including potentially 75 single-cells. **b** Data points generation for the object in (**a**) by random sampling (see text for details), 9240 data points generated. **c** A less complex object with only 6 potential cells. **d** Data points generation for object in (**c**), 740 data points generated. The number of generated data points is proportional to the number of the complex object centroids, thus it depends on the object’s structural complexity (number of potential cells included). Also, more data points are randomly “thrown” around the cells’ medial axes, so as to best represent cell structures (see text for details)

In order to outline the detected cells as accurately as possible and improve the segmentation result, we apply a machine learning method based on Gaussian Mixture Modeling (GMM) [45]. In [45], we have shown how to transform image pixel intensities to a properly constructed set of data points before applying GMM to identify protein spots in a 2D electrophoresis (2DGE) gel image. In our case, pixel intensities provide no information about cell structure since they follow a uniform distribution (see Fig. 5 (a)). So we use the minimum distance from cell boundary (Euclidean distance transform [46], see Fig. 5 (b)) as the basis for data points generation. Specifically, we consider each pixel of the object acting as a data points generator in its neighborhood (refer to Fig. 6). The total number of data points, *N,* to be generated by random sampling and used to represent each object will be proportional to the number *C* of its estimated cell centroids. These *N* data points are apportioned to the pixels of the object according to each pixel’s Euclidean distance from the object’s boundary, meaning that more internal pixels will be allowed to “throw” more data points in their neighborhood. Following this data generation scheme, the pixels closer to the object’s centerline belong to it with higher probability than the more distant ones.

We may think of this process (moving from pixel intensities to data points) as a reverse engineering step where the resulting data points represent best the bacterial shape. Specifically, a pixel *i* located at coordinates *(x*_*i*_ *, y*_*i*_ *)* with distance *d*_*i*_ acts as a data generator using a 2-D Gaussian model *N(μ,* ***Σ*** *)* with center *μ = (x*_*i*_ *, y*_*i*_ *).* Finally, we get a GMM [47] having as many Gaussian components as the number of the pixels *M* in the object. Each component has a mixing coefficient proportional to its distance value and equal to:

*π*(*i*) = *d*_*i*_/∑_*j* = 1_^*M*^ *d*_*j*_.

So for each pixel *i* of the object we draw *N∙π(i)* data points from a 2-D Gaussian distribution which is centered at the pixel’s location *μ = (x*_*i*_ *, y*_*i*_ *)* with diagonal covariance matrix ***Σ*** having its elements set to 0.3. The variance value (0.3) was selected to be smaller than 0.5 (half-distance between neighboring pixels) in order to ensure that data points generated by the model (representing “cell structure”) will be distributed in a manner that guarantees that their abundance reflects the distance, thus preventing the generation of “hills” of data points in-between pixel locations. This variance value was determined by experimentation and is kept fixed throughout the analysis, irrespectively of the image modality.

As mentioned before, the number N of data points generated for a complex object is proportional to the number of the estimated candidate cell centers C it may contain. In particular we use

$$ N=\frac{1}{2}\  C\ \frac{\  C ellLength\kern0.5em \times \kern0.5em  C ellWidth}{CalFacto{ r}^2}, $$

where *CellLength* is the expected cell length, *CellWidth* is the expected cell width of the species under examination and can be estimated from literature. For example for *Salmonella* S. Typhimurium the width (diameter) range considered is [0.7, 1.5] μm (so we chose *CellWidth* = 1.1 μm), and in length range is [2, 5] μm (so we choose *CellLength* = 3.5 μm) based on literature [48]. Let us remark that even if these cell size parameter values are overestimated the approach will suffer only from a performance loss (e.g. by using a larger than needed *N* value). Moreover, *CalFactor* is the spatial calibration factor of the experiment’s microscope used to convert the size of an image object from pixels to physical units (μm).

Using an *N* value that is proportional to C was a deliberate choice because the number of identified candidate centers can act as an object’s complexity indicator approximating the number of single-cell structures expected to be present in a complex object's region. So, if a complex object contains a lot of candidate centers, it is justified to “throw” more data points (use a larger *N*) in order to capture adequately the underlying structure of the different single-cells included in it. As shown in Fig. 6, the generated data set for the object with the six candidate centers (Fig. 6(c)) "spent" less data points (Fig. 6(d)) than the object with the 75 candidate centers (see Fig. 6(a) and (b)).

After generating the data points we associate each one of them with its closest center using Euclidean distance [42] and nearest neighbor classification [49]. Starting with this initial assignment, we can determine the initial parameters of a 2-D GMM having *C* components (as many as the estimated cell centroids in the object) and compute the log-likelihood of each data point to belong to this initial mixture model:

$$ \log\ \mathrm{p}\left({\mathbf{y}}^{(i)}\left|\varTheta \right.\right)= \log {\displaystyle \sum_{\mathrm{m}=1}^C{w}_m\mathrm{p}\left({\mathbf{y}}^{(i)}\left|{\theta}_{\mathrm{m}}\right.\right)} $$,

where *Θ*≡{*θ*_1_, …, *θ*_*c*_, *w*_1_, …, *w*_*c*_} is the complete set of mixture model parameters and *w*_*m*_ are the mixing coefficients.

**Fig. 7:**
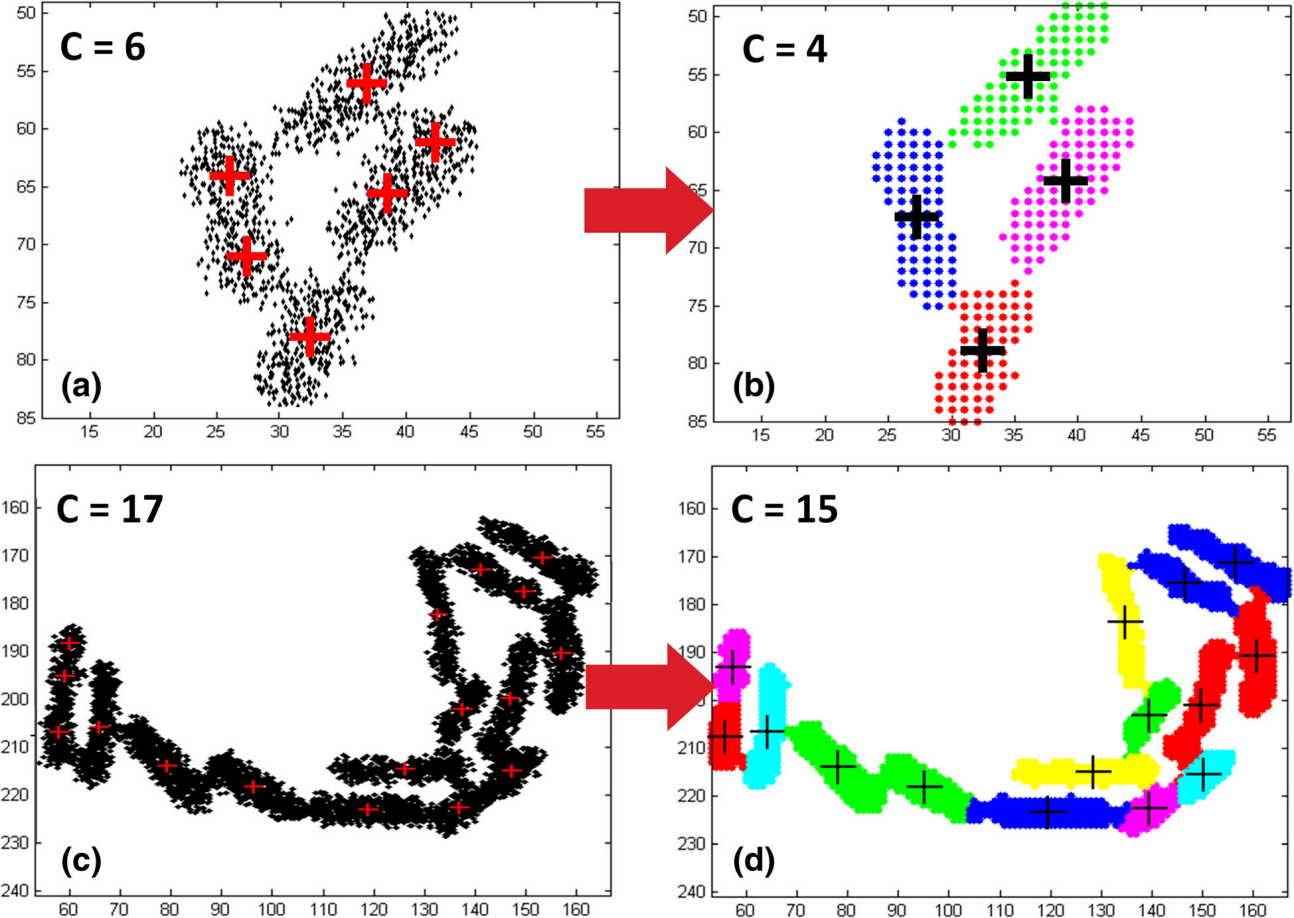
Cells segmentation - Best Gaussian Mixtures Model fit. Initialization of Gaussian mixture model parameters is performed after associating data points to cluster centers using nearest neighbor classification. For the complex object in (**a**) we have initially *C* = 6 components (clusters) in the mixture and for the model in (**c**) *C* = 17 components. We then apply the Expectation-Maximization (EM) algorithm and use the Minimum Message Length (MML) model selection criterion to identify the number of mixture model components that produces the best model fit. In (**b**) this results to a reduction of clusters from 6–4, and in (**d**) from 17–15. Each component in the final model represents a segmented single-cell (see text for details)

The next step is to apply Finite Mixture Modeling (FMM) [50] in two dimensions (see Fig. 7). An important issue in mixture modeling is the selection of the proper number of components to use. With too many components the mixture may overfit the data, while with too few components it may not be flexible enough to capture the true underlying reality [50, 51]. In our case, this would translate to a solution with either more or less components than the actual number of cells present inside a complex object. In order to overcome this difficulty we apply a modified Expectation Maximization (EM) algorithm [50] which also employs the Minimum Message Length (MML) criterion [51] for best model selection. However, since the EM with MML is computationally expensive we first apply the "puzzle solving" algorithm to estimate the initial number of components properly i.e. start the iterative algorithm at a number not too far from that of the best model. Using the MML criterion ensures that the best model will not end up being an unnecessarily complex one unless if it pays for itself. Therefore, it is possible that the best model may end up containing less than the initial number of C components (see Fig. 7). At the end, it is possible that some circular object fragments may still remain isolated and not merged with the cell they really belong to, especially when analyzing low resolution cell movies. To overcome this difficulty, we check if a fragment’s area is under a pre-specified threshold

$$ A=\frac{1}{4}\pi \cdot {\left(\frac{CellWidth}{CalFactor}\right)}^2 $$

determined automatically by the pipeline using the pre-specified parameters. Fragments with area less than *A* are merged with the "touching" neighbor that maximizes the resulting object's solidity after the merging (as mentioned in the "puzzle solving" step of the analysis) otherwise they remain intact and are considered to be undersized single-cells. Intuitively, considering a cell's projection on the plane as a curved parallelogram with two attached semi-circles (poles), its area must be at least the area of the two cell poles, i.e. the area of a circle with radius equal to one half of the expected cell's width as in *A*.

#### Lineage tree construction

A cell in a time-lapse movie frame can: 1) proliferate, 2) divide, and 3) die or disappear from the microscope’s field of view. In order to construct the lineage of a single cell, first we have to segment the cells of each colony efficiently and then we have to track the colonies and their cells along consecutive frames. In order to track cells in overcrowded bacterial time-lapse movies, we have developed an algorithm inspired by motion estimation techniques used for video compression [52] which also follows a divide-and-conquer strategy. Our cell tracking algorithm will not be discussed here since it has been published in [53].

#### Single-cell and colony properties extraction

For each colony, we compute growth curves in terms of the colony’s area (either in pixels or in micrometers) or in terms of cell population (cell counts). In addition, we extract numerous properties at the single-cell level. We divide single-cell properties into two categories: cell *attributes* and cell *life attributes. Attributes* are cell properties changing with time, characterizing each cell at each frame of the time lapse movie. For example, cell attributes that we track are: area, fluorescent protein quantity (if any), major axis length (length), minor axis length (width), distance from the colony’s centroid etc. On the other hand, cell *Life attributes* are statistics of attributes (e.g. min, max, average, median, standard deviation) characterizing a cell’s whole life "trajectory", i.e. from its birth to its division. In addition, elongation (i.e. how much a cell has changed length before it divides) and division time (i.e. the duration of a cell’s life) are life attributes too. Additionally, given an attribute’s evolution (time-series) we can estimate life attributes e.g. estimate an individual cell’s elongation rate by fitting an exponential model to the cell’s trajectory. All these properties are estimated and exported (stored) to a bio-database for each individual cell and time frame in the movie. Moreover, several useful visualizations can be produced for displaying the computed cell attribute values directly using color on top of the input time-lapse movie videos (refer to Additional files 4, 5 and 6) or on top of a colony’s lineage tree or divisions tree as it will be demonstrated in the Results and Discussion section.



**Additional file 4:** Computed cell length overlaid on SalPhase time-lapse movie. (MP4 792 kb)

**Additional file 5:** Multi-SalPhase time-lapse movie; cell length visualization after segmentation. (MP4 6740 kb)

**Additional file 6:** SalPhase time-lapse movie; color indicates cell generation index as determined by the cell tracking algorithm. (MP4 516 kb)


#### Implementation

The pipeline of algorithms presented here has been coded in Matlab version R2015b [54]. We used extensively the image processing, statistics and machine learning, wavelet and curve fitting Matlab toolboxes. All presented results were obtained using a desktop computer with Intel Core i5-3350 processor running at 3.10 GHz, 8GB RAM, under Microsoft Windows 7 professional operating system. The current implementation is not optimized for performance. Nevertheless, a discussion of the trends of its running time is provided in Supplementary Material section 5 (see Additional file 1). Due to the divide-and-conquer strategy followed that breaks large problems into smaller subproblems, we expect that the use of parallel processing can significantly accelerate the analysis. All post-processing capabilities are also implemented in Matlab, so as to enable users to interact with the data produced using simple function calls. The authors' intention is to complete the software integration and performance optimization, add a proper user interface and then make the software accessible to the systems biology community. The emphasis of this methodology paper is on the innovative methods integrated in the BaSCA pipeline and not on the software architecture and implementation.

## Results and discussion

### Comparison to the state of the art

In order to compare the segmentation performance of the proposed pipeline with state-of-the-art methods, we chose software packages that are extensively used by different labs worldwide to analyze cell movies and that have been referenced in several studies [55–66]. Moreover, we analyzed representative still (single) frames provided by the state-of-the-art software packages at their websites (see Materials), as well as two more images, a frame from our SalPhase movie (frame 74) and a frame from our Multi-SalPhase movie (frame 78). The Recall, Precision and F-measure was computed for each dataset and method used in the comparative evaluation.

**Fig. 8:**
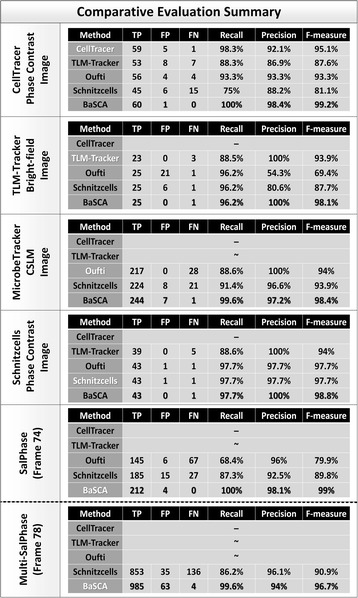
Comparative Evaluation Summary. Each section of the table reports the evaluation results for an image suggested by one of the methods under comparison. The table columns list the true positives (TP), false positives (FP), false negatives (FN), as well as the Recall, Precision and F-measure achieved by each method. SalPhase frame 74 and Multi-SalPhase frame 78 were used to assess the performance of the methods on images with dense and overcrowded colonies. Dashes (-) indicate failure to return results for a specific dataset. Tildes (~) indicate very poor performance. The proposed method (BaSCA) achieved consistently very high F-measure (≥97.3% for all cases), suggesting that it is robust across imaging modalities and datasets produced by different labs. (Refer to Additional file 1: Figures S1-S5 for the detailed segmentation results)

The table in Fig. 8 summarizes the evaluation results. Dashes (tildes) in table entries indicate that the specific method did not return results (or gave very low quality results) respectively. CellTracer was able to analyze only the frame downloaded from the CellTracer’s movie. TLM Tracker performed well with images having only one small-size colony in the field of view (such as in CellTracer’s and Schnitzcells’ images). Oufti and Schnitzcells were the most robust among the state-of-the-art methods, returning results for all images. The proposed pipeline exhibited a notable F-measure advantage for all imaging modalities and datasets used; its F-measure remains very high (over 96.7%) even for images with multiple overcrowded colonies in the field of view. Moreover, it outperformed the other approaches even when using images from their own repertoire. Only BaSCA returned near perfect results (F-measure of 99%) for the SalPhase movie, achieving perfect recall and an almost 10% higher F-measure than Oufti. In Multi-SalPhase, a movie containing many overcrowded colonies in the field of view, BaSCA achieved F-measure 96.7% in comparison with the state-of-the-art software, Schnitzcells which achieved 90.9%. Oufti did not provide reliable segmentation results for this dataset. The results of the comparative evaluation for all methods along with the used parameterizations and input images are provided for each dataset as Supplementary Material (see Additional file 7).

**Fig. 9:**
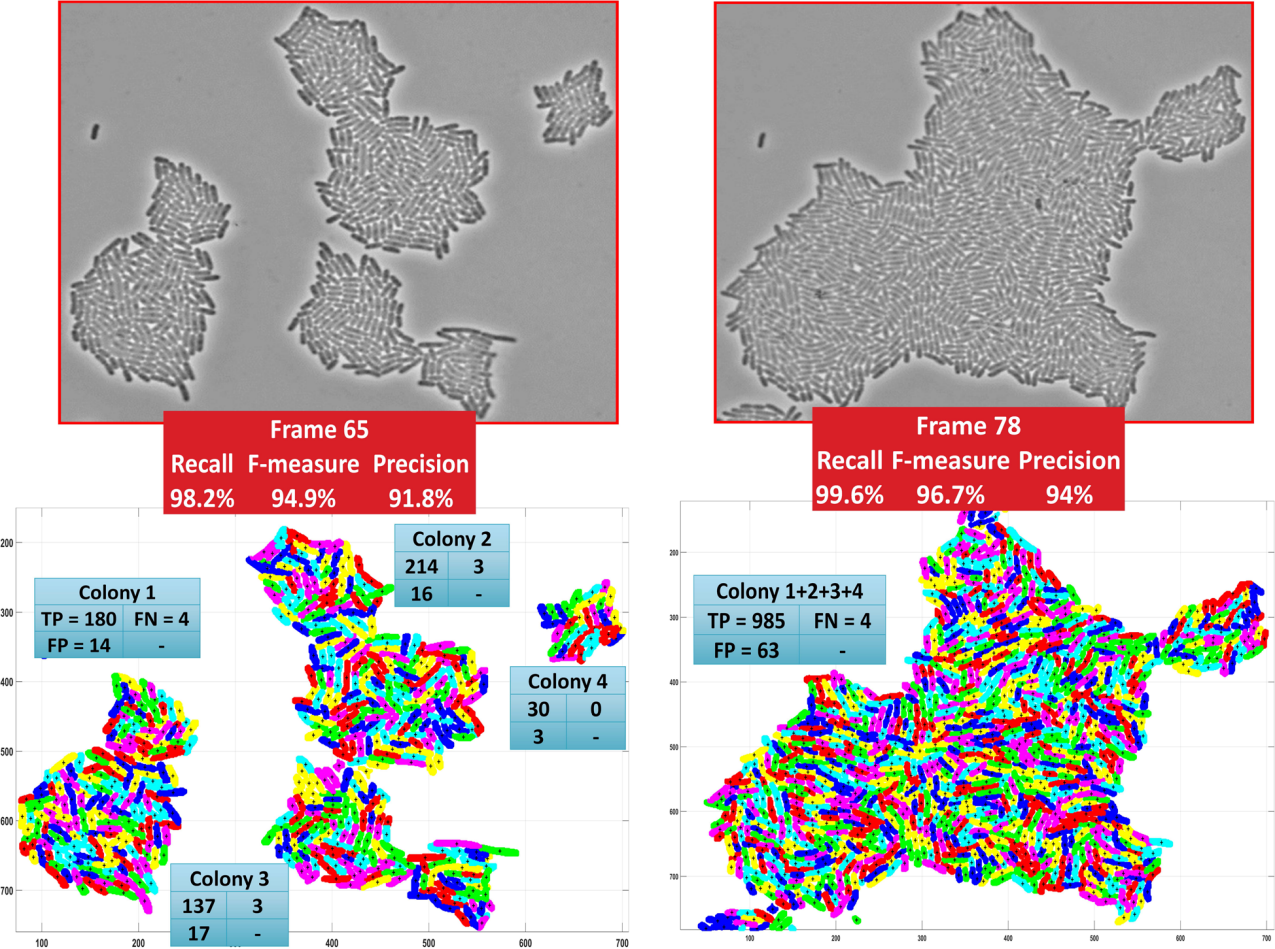
Segmentation of overcrowded and merging colonies. The four colonies in frame 65 of movie Multi-SalPhase (left) are merged several frames later (frame 78, right). *Top panels:* input image frames. *Bottom panels*: Segmentation results: The *cyan boxes* report the TP, FN and FP for each colony. The *red boxes* summarize the evaluation measures for each frame. Pseudo-colors are used to make cell boundaries visible. BaSCA achieves both high recall and high precision and an F-measure over 94%

One of the main contributions of this work is that, in contrast to the state-of-the-art methods, BaSCA can segment successfully images with multiple overcrowded colonies as they grow and merge, potentially with thousands of bacteria in a field of view (see Additional file 5 providing a video with the segmentation results of Multi-SalPhase movie). Figure 9 illustrates this fact. Segmentation accuracy when analyzing frames with overcrowded colonies is almost as high as in non-overcrowded frames. Recall remains over 98%, precision over 91% and the F-measure over 94%. Due to its high segmentation robustness, BaSCA can estimate several time-varying colony and single-cell level properties accurately fulfilling the expectations of image driven single-cell analytics for predictive microbiology.

We should emphasize that the state-of-the-art software packages used in the evaluation require a different parameterization for each imaging modality. Thus, we had to perform extensive experimentation to find the most appropriate parameter settings for each dataset before the evaluation (see Additional file 7 for the parameterization of each software and datasets). This is a laborious task that requires the user has knowledge of image processing concepts e.g. to select filter types, set parameters, select segmentation methods etc. (refer to Additional file 1: Table S1). In contrast, the parameterization of BaSCA is trivial. All parameters used by the pipeline are set automatically once the user inputs the spatial calibration factor, image modality and the expected value for the average cell length and cell width (diameter) of the species under examination as discussed in Methods. Eliminating the need for an elaborate parameterization was a deliberate choice since our objective was for the pipeline to be used trivially by microbiologists with no expertise in image analysis and to be easy to integrate into larger service workflows for high throughput single-cell analytics and systems biology.

In summary, BaSCA extends the current state of the art by being: (a) more accurate, (b) more versatile since it can process datasets produced using different microscopy types/imaging modalities and return trustworthy results, (c) high throughput, automated, without requiring a human in the loop, and (d) able to analyze efficiently and at many levels cell movies with multiple colonies that grow and merge, resulting in overcrowded bacterial communities with thousands of cells in the field of view.

### Comparison to the ground truth

**Fig. 10:**
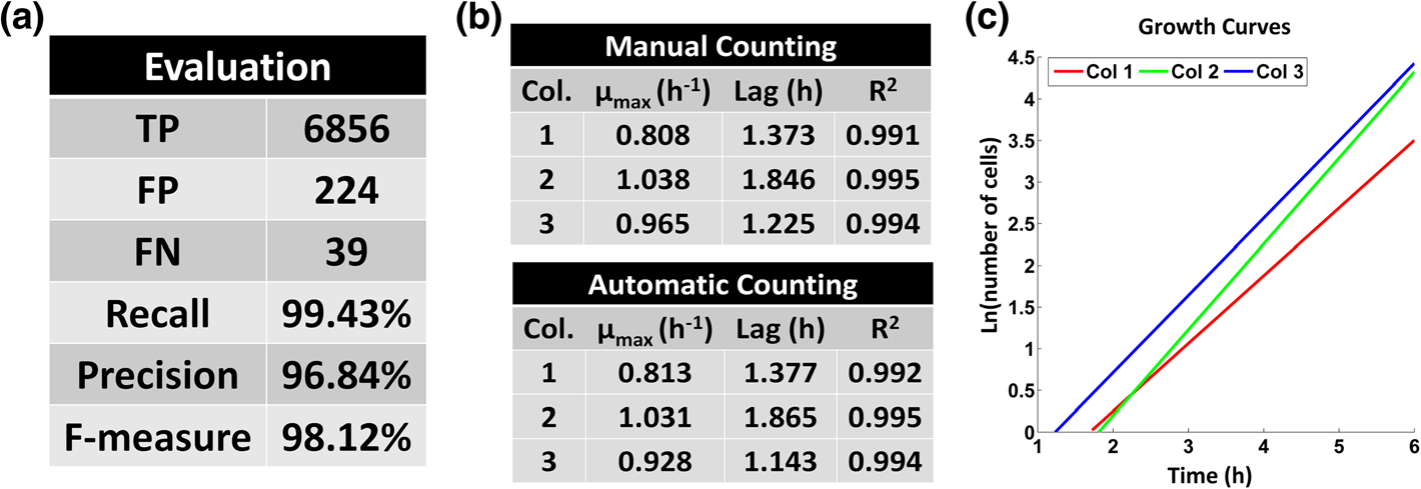
Growth rates - Evaluation w.r.t. the ground truth. **a** Evaluation results summary for the SalPhase movie (86 frames). The developed pipeline achieves a very high F-measure above 98%, (**b**) Comparison of manual vs. automatic BaSCA counting for the three colonies of the SalPhase movie by fitting a *Baranyi and Roberts* model [43]; the kinetic parameters of microbial growth are almost identical, (**c**) Automatically estimated growth curves of the three micro-colonies in the dataset

In Fig. 10(a), we present a summary of the comparison of our pipeline's results versus the ground truth for the whole SalPhase movie (86 frames). We observe that BaSCA achieves a recall over 99% and precision over 96%. Overall it achieves a very high F-measure of 98%. In this movie we have accounted correctly for 6856 out of the 6895 true cells and made only 263 errors, mostly false positives. The results per frame and per colony are provided in Additional file 8. It is significant that the proposed methodology achieves a very high F-measure score (>93.27%) in all frames, which proves its segmentation robustness. The segmentation results for the frame with the lowest F-measure (frame 63) are provided in (Additional file 1: Figure S1). The majority of the errors appear during the exponential phase (after the 8^th^ cell generation); beyond this point the colonies start growing in the 3^rd^ dimension. In summary, the results suggest that the developed pipeline remains both very sensitive and very precise as long as colonies maintain their 2-Dimension structure.

BaSCA can provide, in a high throughput no-human in the loop manner, accurate data to support predictive modeling for systems microbiology. In order to demonstrate this important capability we also fit the primary model of *Baranyi and Roberts* [67] to the growth curves of each colony in the SalPhase movie. This mathematical model is commonly employed by microbiologists to estimate growth kinetic parameters of a cell population in a given environment. The kinetics parameters of the population are its lag time (*λ*) and the maximum specific growth rate (*μ*_*max*_). To describe the abrupt transition of the observed cell growth from the lag to the exponential phase the values of the model parameters *m* and *n* were fixed to 0 and 20 respectively as in [13]. In Fig. 10(b), we present the estimated kinetic parameters for the three growing colonies in the SalPhase movie extracted using manual and automatic cell counting. We can see that the kinetic parameters using the ground truth (manual counting) are almost identical to those estimated using automated BaSCA counting. In Fig. 10(c), we observe that although in the movie we have three colonies emanating from three single-cells and growing concurrently in the same micro-environment, their growth dynamics are quite different as also shown in [13]. Consequently, it becomes apparent that our analysis pipeline not only provides trustworthy results without any human involvement, but also provides a useful tool for characterizing the stochasticity exhibited in colony dynamics. This can accelerate predictive microbiology studies that try to elucidate the functional role of stochasticity [3, 4, 13–15] and how it is affected by community effects [6, 7, 16, 17].

### Single-cell analytics and visual exploration

**Fig. 11:**
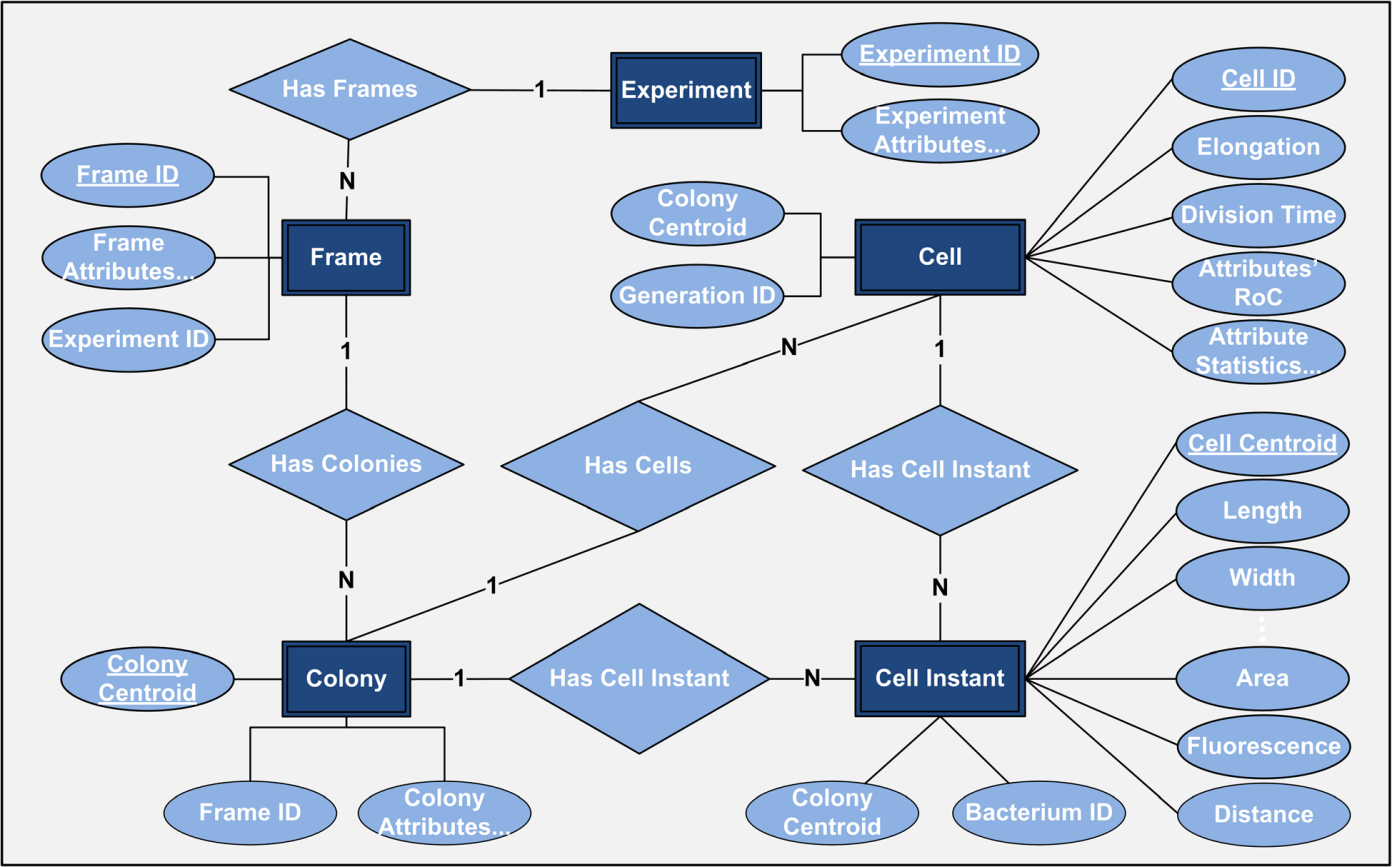
Single-cell analytics database ER-Diagram. Organization of the database storing information about the experiment that generated the cell movie (Experiment table) and the time lapse microscopy characteristics (Frame table). In addition, we store the image analysis generated information for each colony in the field of view (Colony Table) and for each segmented cell within each colony. Specifically, single-cell *attribute* values changing at every time point are stored in the Cell Instant Table, while cell *life attributes* that characterize the whole cell life trajectory are stored in the Cell Table. The database summarizes the cell movie image analysis completely and can be used for downstream single-cell analytics and visualization. Moreover it forms the basis for building repositories of cell movies under different conditions for large scale high throughput systems microbiology experiments

Accurate and automated image analysis for large size bacterial communities enables single-cell analytics and high throughput systems microbiology. In Fig. 11, we present the Entity-Relationship (ER) model [68] of a database schema that supports the image-driven single-cell analytics system developed. In this bio-database representation of a cell movie, we capture essential information regarding the settings of the underlying experiment (Experiment table), and the time lapse microscopy characteristics (Frame table). In addition, we store a plethora of properties extracted by the image analysis for each colony in the field of view (Colony Table), as well as for each segmented single-cell of each colony (Cell Instant Table and Cell Table). Specifically, we store single-cell properties that may change at every time instant (*cell attributes*) as well as properties that characterize a cell’s whole life span, from birth to division time (*cell life attributes*). For example, in the Cell Instant Table we store extracted cell attributes (e.g. cell area), while in the Cell Table, we store the estimated/measured cell life attributes (e.g. the cell's elongation, division time etc.). The bio-database stores the "big data" that result from the analysis performed at different levels (frame, colony, and single-cell), providing essential meta-data for the experiment that can accompany the imaging data, and/or used in lieu of the imaging data in subsequent analytics and modeling efforts. This unique aspect of BaSCA not only enables image-driven systems microbiology, but provides a mechanism for reducing the cell movies massive data down to the useful information that can be exploitable by downstream processes. This is a very useful feature for building repositories of annotated cell movies with meta-data that can be searchable and usable in systems biology workflows over the internet.

**Fig. 12:**
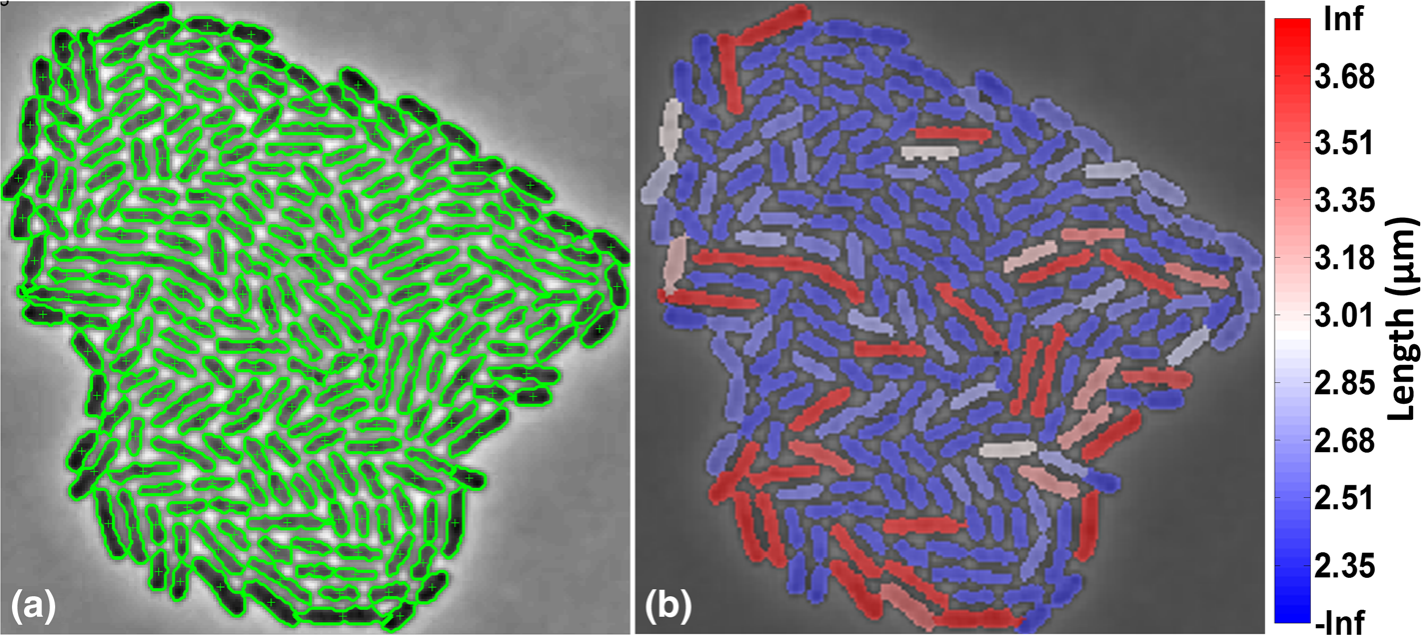
Cell attribute visualization. **a** BaSCA segmentation results for colony 3 of the SalPhase movie (frame 86). *Green curvatures* mark the contours of segmented cells. **b** Cell length visualization using color, overlaid on each segmented cell. Movies of cell length visualization are provided in Additional file 4 and 5 for SalPhase and Multi-SalPhase datasets respectively

In Fig. 12, we present a visualization of the cell segmentation results. We observe (Fig. 12(a)) that segmentation is accurate since the overlaid green contours outline the real contour of each cell. As a consequence, we can have confidence that we can measure cell attributes correctly. In Fig. 12(b, we show how a cell attribute (e.g. cell length) can be visualized using BaSCA. The different colors represent cell length (in micrometers, μm), in the range indicated by the color bar. This kind of simple visualization allows the user to distinguish spatial patterns, or even focus on specific bacteria according to the chosen cell attribute of interest. Moreover as a byproduct of the multi-scale image analysis BaSCA can generate time-lapse movies of segmented growing colonies that animate the visualization of any extracted cell attribute of the user's choice. Additional files 4 and 5 provide video animations of the space-time evolution of the cell length attribute for the SalPhase and Multi-SalPhase cell movies, respectively.

Automated lineage tree construction of large-size over-crowded colonies is also an important requirement for high throughput systems microbiology. CellTracer, TLM-Tracker and Schnitzcells support lineage tree construction; however, they are limited to colonies with a small number of cells and do not exploit lineage trees as drivers for single-cell visual analytics [53].

**Fig. 13:**
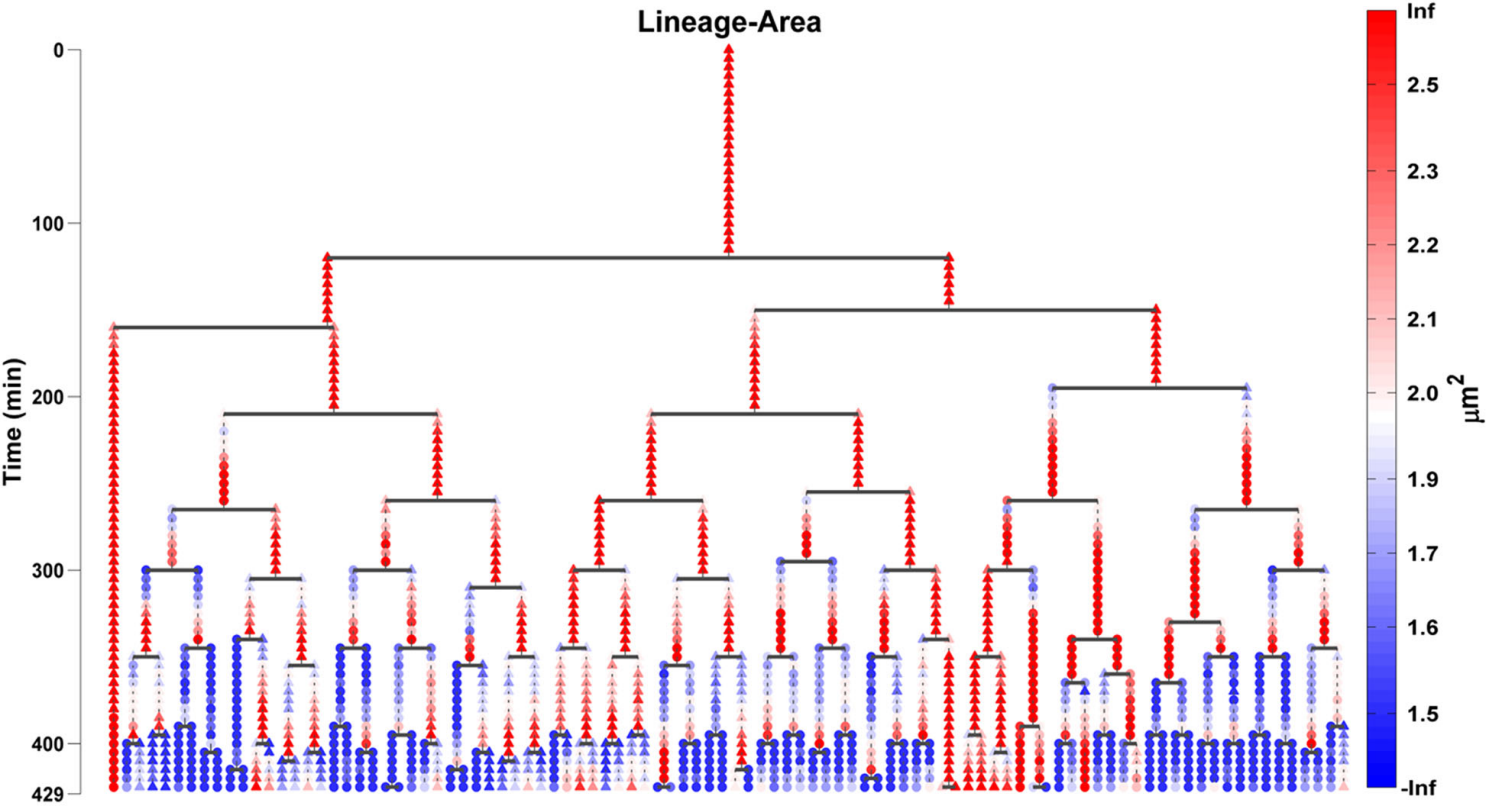
Single-cell attribute evolution visualization on the lineage tree. The lineage tree of colony 1 (top left) of the SalPhase movie. The area attribute is visualized using color on the tree for every cell and time instant (frame) of the movie. Triangular (circular) shape node glyphs are used for time instants that a cell lies in the colony’s boundary or within the colony respectively. Any cell attribute available in the database produced by the image analysis can be visualized in the same manner

**Fig. 14:**
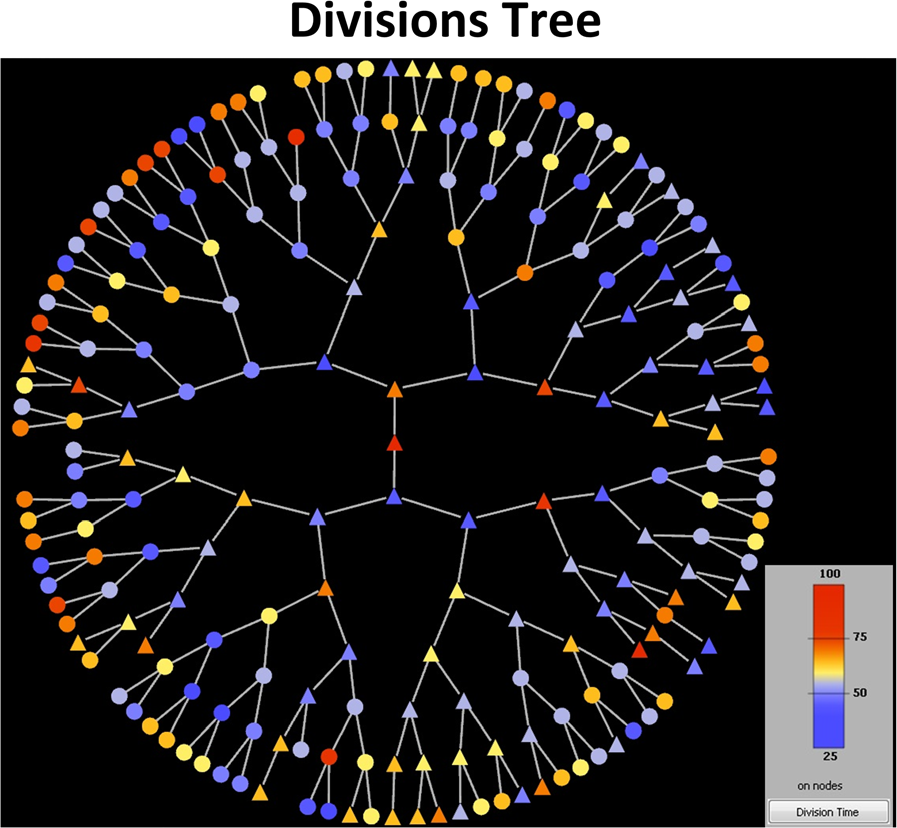
Cell life attribute visualization on the cell divisions tree. A circular tree of cell divisions (root cell in the middle) for colony 3 of the SalPhase cell movie. Colors represent here division times (min) as indicated by the color bar. We can easily assess visually how division times vary along tree branches (cell clones) and tree levels (cell generations). Triangular (circular) nodes represent cells that lie on the colony’s boundary (within the colony) respectively. Any cell life attribute available in the database produced by the image analysis can be visualized in the same manner. The Figure was created using the Tulip software package [67]

Our methodology allows users to exploit lineage tree construction to visualize the evolution of single-cell attributes extracted from the automated image analysis of time-lapse movies. For example, we can visualize on top of the lineage tree the evolution of single-cell area in a colony (see Fig. 13), and thus inspect easily how it tends to change during the cells' life span. Moreover, we can visualize the evolution of cell life attributes e.g. their division time. We achieve this by constructing a condensed circular tree, called divisions’ tree, containing as nodes only the cell division events. In Fig. 14, we use Tulip [69] to visualize on the divisions tree, the cell division times of the 3rd colony cells of the SalPhase movie. This is a very useful visual analytics feature since it can help us to quickly assess how cell life attributes evolve across generations under different experimental conditions.

### Image-driven single-cell analytics support systems microbiology

**Fig. 15:**
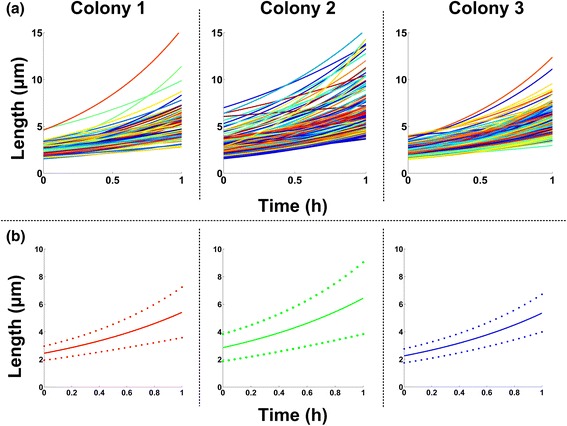
Single-Cell Exponential Growth curves. **a** Single-cell growth curves estimated from the image-analysis data after fitting an exponential individual cell length model (see text for details). Different colors represent different single-cell lifespan trajectories. **b** Average cell growth curves of each colony (*solid lines*). Dashed lines represent one standard deviation above and below the average curve. We observe that cell growth exhibits considerable variability among colonies, and cells within the same colony (SalPhase cell movie)

Using the results of the analysis at the single-cell level, i.e. cell attributes and cell life attributes, allows us to verify literature results and formulate interesting new hypothesis for future research. For example, given the extracted lineage tree of a colony and the estimated length of every cell at every time instant, we can fit to each cell's length trajectory (time series) an exponential model [70, 71]. Specifically, let us consider as in [71] a single-cell length model of the form *l*_*t*_ = *l*_0_ ⋅ *e* ^*kt*^, where *l*_*0*_ is the birth length of a stalked cell and *k* is its growth rate (cell elongation rate). In this way we can capture the growth characteristics of each cell and estimate its *personalized* kinetic parameters. In Fig. 15(a) we provide the growth curves of each individual cell of the SalPhase movie estimated using non-linear least squares [72]. We observe that single-cell growth kinetics exhibit great variability, something that cannot be observed by population based experiments. In Fig. 15(a) we provide the average single-cell growth curves for each colony of the SalPhase movie. We notice that average growth curves vary among colonies of the same movie that grow in the same micro-environment under the same experimental conditions. Clearly, colony 3 exhibits less heterogeneity (smaller variance) than the rest of the colonies. Single-cell analytics enables zooming in and extracting useful information regarding subpopulation characterization for any cell attribute of interest among the many extracted by BaSCA.

**Fig. 16:**
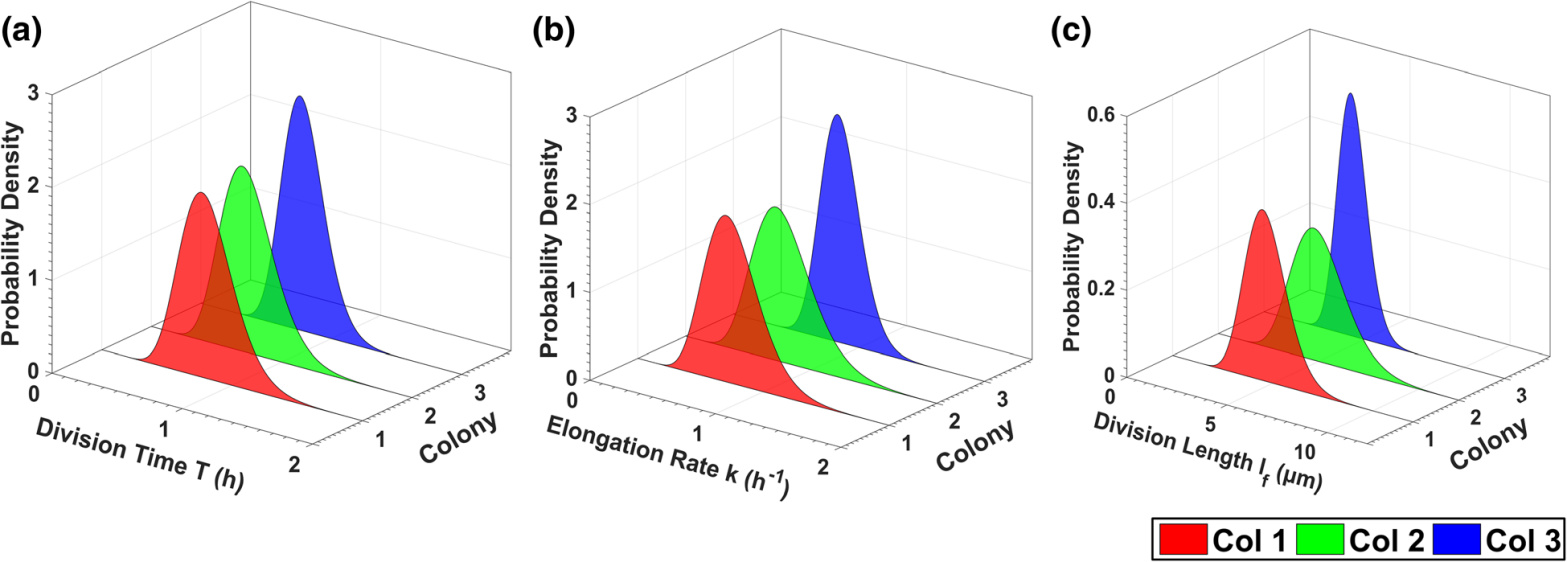
Life attributes variability per colony. Gamma distributions (best fit) of (**a**) the cell division time *T*, (**b**) the cell elongation rate *k*, (**c**) the cell division length *l*_*f*_ for colonies 1, 2 and 3 (SalPhase movie). The proposed methodology allows us to characterize the variability of cell life attributes across colonies. Similar analysis can be performed for any life attribute available in the database after image analysis using BaSCA

Single-cell analytics also allows characterization of intra and inter-colony as well as intra and inter-generation variability of cell life attributes. In Fig. 16, we present the best gamma distribution fits of the single-cell elongation rate *k,* division time *T*, and cell length at division *l*_*f*_ per colony (cells from 3^rd^ to 8^th^ generation were pooled, SalPhase movie). The estimated best model parameters are provided in (Additional file 1: Table S2). We observe that the cell life attribute distributions of the 3rd colony have a lower variance than the rest of the colonies. Intuitively, this suggests that the cells of the 3rd colony grow and divide more synchronously across generations. This is actually confirmed by examining the cell generations movie, i.e. a version of the SalPhase movie in which we have colored cells according to their generation index at any instance as determined by our automated tracking and lineage tree construction algorithms (see generations movie in Additional file 6). Such subpopulation investigations would be impossible without a single-cell analytics focus; our image-driven single-cell analytics approach enables zooming-in and characterizing cell properties at any desirable level of community organization, in space (colonies) or in time (subtrees of the lineage tree).

**Fig. 17:**
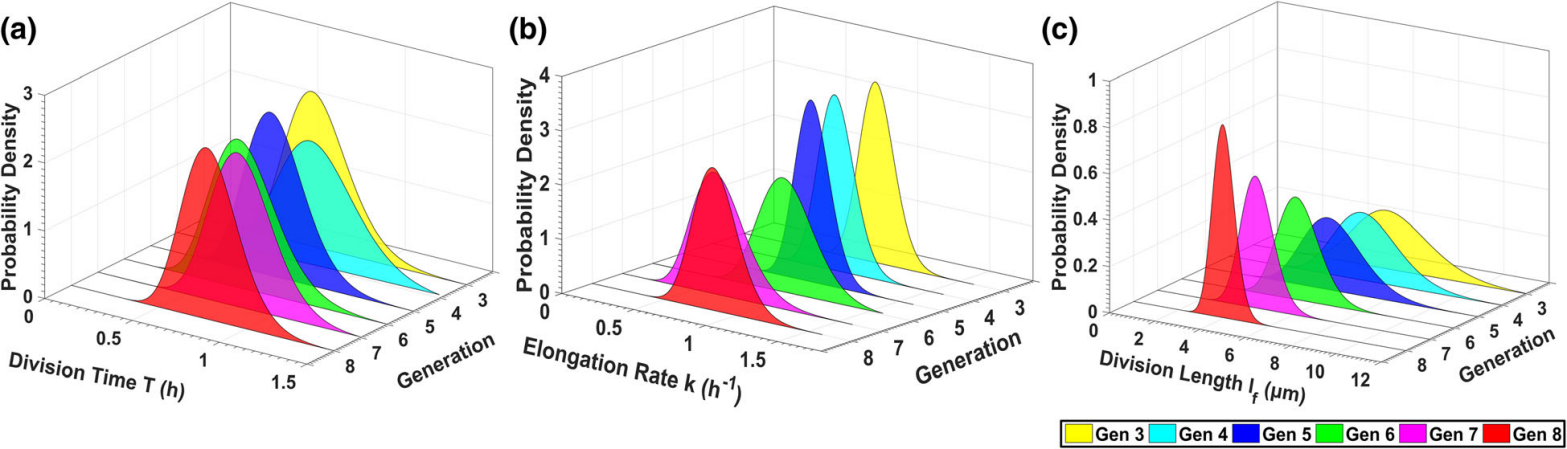
Life attributes variability per generation. Gamma distributions (best fit) of (**a**) the cells division time *T*, (**b**) the cell elongation rate *k*, and (**c**) the cell length at division *l*_*f*_ for the 3^rd^ to the 8^th^ generation of cells of the SalPhase movie (cells from all colonies pooled). By delving into each generation’s individuals we can characterize a life attribute's intra and inter-generation variability (stochasticity). Similar analysis can be performed for any life attribute available in the database after image analysis using BaSCA

In Fig. 17, we provide the best fit Gamma distributions of the aforementioned cell life attributes, but now per cell generation (cell data from the three colonies are pooled, SalPhase movie). We chose the Gamma distribution because the histograms of the aforementioned attributes have similar shape (skewness) to it (in Additional file 1: Figure S2 and Figure S3). Moreover, the gamma distribution is a flexible two-parameter distribution that belongs to exponential family and is used to model physical quantities that take positive values in microbiology, such as the cell division time (as in [14, 73]), the cell elongation rate (as in [73] and cell division length (as in [71]). The estimated parameters are provided in (see Additional file 1: Table S3). In Fig. 17(c) we observe that there is a trend towards lower mean and smaller variance for the cell division length attribute as the generation index increases. Single-cell analytics offer the capability to quantify the stochasticity and examine inter and intra-generation variability by estimating epigenetic correlations of single-cell attributes.

However, the division length does not correlate explicitly neither with the elongation rate nor with the division time, since the estimated Pearson correlation coefficients are very low for both cases *(r* = 0.2 and *r* = 0.01, respectively for the SalPhase movie). This is a rational result because cells growth depends both on the elongation rate and on the life span (i.e. division time) of the cell simultaneously, as shown in [70, 71], factors which are both epigenetic information inherited by the cell's progeny as mentioned in [11, 12]. So, we cannot assume that a cell that is going to live longer, i.e. that will have higher division time, will necessarily become lengthier too, given a specific birth length. In order to demonstrate this we computed the correlation coefficients of cell life attributes estimated by our image analysis and compared them to corresponding coefficients estimated in [70] where *E. coli* cells were grown individually in microfluidic devices. Specifically, we considered single-cell elongation, defined as $E\kern0.5em =\kern0.5em  log\frac{l_f}{l_0}\kern0.5em =\kern0.5em  k\cdot T$, and computed its correlation to the natural logarithm of the cellular birth length (*ln*(*l*_0_)). The Pearson correlation coefficient was found to be *r* = -0.44 (see Additional file 1: Figure S4(a)), and this is in striking accordance to what was found in [70] (see Fig. 2 of [70]). The anti-correlation between elongation and cell birth length (see Additional file 1: Figure S4(a)) is entirely due to a modulation of the division time, with the elongation rate appearing to be independent of birth length (see also Additional file 1: Figure S4(b-c)) as it was also found in [70]. It is also interesting that the correlation coefficients of (*ln*(*l*_0_)) to cell division time *(T)* and elongation rate *(k)* we found through single-cell analysis match closely the values reported in [70] (see Additional file 1: Figure S4(b-c)). These findings verify that BaSCA algorithms quantify accurately single-cell properties when analyzing bacterial colonies growing in microenvironments accounting for the community dynamics.

## Conclusions

We presented BaSCA, a novel Bacterial image analysis driven Single Cell Analytics pipeline which enables the high throughput analysis, down to the single-cell level, of complex time lapse cell movies with many colonies and potentially thousands of cells in the field of view. The results presented demonstrate its robustness and universality. In contrast to other methods, BaSCA is fully automated and does not require users to be familiar with image processing concepts and/or provide an elaborate parameterization to get good results any time a new movie is analyzed. BaSCA not only improves the accuracy of segmentation but also achieves this without requiring a human in the loop. Moreover, it tracks effectively colonies and single-cells as they grow and divide to form overcrowded bacterial communities and extracts a plethora of colony and single-cell properties on the fly.

BaSCA is the first bacterial image analysis methodology designed with high throughput single-cell analytics in mind. It thus provides an important tool for dissecting the phenotypic diversity at different levels of community organization and understanding how inter-cellular interactions play a role on important phenomena for human health, such as biofilms formation, persister cells emergence etc.

As it has been demonstrated, the proposed methodology introduces several single-cell data analytics capabilities. It is designed for high throughput and enables systems biology orientated research both at a higher resolution (i.e. zooming into the single-cell level) and at a larger scale (communities with many dense colonies). It can therefore be used to study the role of single-cell stochasticity without losing sight of community effects that may drive it.

Work in progress includes algorithmic improvements, e.g. to allow segmentation of movies with filamentous cells (as in Oufti [22]) and the design of a suitable user interface. Moreover we are optimizing the performance of BaSCA by exploiting the divide-and-conquer nature of the segmentation method in order to parallelize the analysis both at the colony and the cell object levels. The ultimate goal is to be able to analyze efficiently and without human intervention stacks of cell movies in a high throughput mode as needed to construct well annotated repositories of cell movies and calibrate mathematical models accounting for single-cell stochasticity, so as to capture and characterize adequately the "logic" of bacterial communities’ behavior under different stress conditions.

## Additional files

Additional file 1: Supplementary Material. (DOCX 1601 kb)

Additional file 7: Contains for each dataset the segmentation results of each method (.tif images) and corresponding parameterization files (.mat files). (ZIP 62299 kb)

Additional file 8: SalPhase time-lapse movie ground truth table. (XLSX 51 kb)

## Abbreviations

BaSCA: Bacterial image analysis driven single cell analytics
CLAHE: Contrast-limited adaptive histogram equalization
CLSM: Confocal laser scanning microscope
EM: Expectation maximization
ER: Entity-relationship
FMM: Finite mixture modeling
FN: False negatives
FP: False positives
GMM: Gaussian mixture modeling
MML: Minimum message length
PPV: Positive predictive value
TP: True positives
TPR: True positive rate
