# Supplementary material for: Image analysis driven single-cell analytics for systems microbiology
Source: BMC Syst Biol. 2017 Apr 4;11:43. doi: 10.1186/s12918-017-0399-z (PMC5379763; doi:10.1186/s12918-017-0399-z)
Supplement: Supplementary file 6 — Supplementary Material. (DOCX 1601 kb) [file 12918_2017_399_MOESM1_ESM.docx]

# Supplementary Material

# Features of cell movie analysis methods

In the table below we summarize the main features for some widely cited projects found in the literature for which the source code is available and compare them to the proposed pipeline.

**Table S1. Features of different approaches**

| **Approaches/ Features** | **CellTracer [1]** | **TLM-Tracker [2]** | **Schnitzcells [3]** | **Oufti [4]** | **Proposed (BaSCA)** |
| --- | --- | --- | --- | --- | --- |
| **Tracking multiple & merging colonies (subpopulations)** | No | No | No | No | Yes |
| **Tracking cells** | Yes | Yes | Yes | Yes | Yes |
| **Robust to low image resolution** | No | No | No | Yes | Yes |
| **Dealing with overcrowded cell movies** | No | No | No | Yes | Yes |
| **Phase contrast/ Fluorescent images** | Phase | Both | Both | Both | Both |
| **Number of Parameters** | Too many | Too many | Many | Too many | Few |
| **Parameterization** | Complex | Complex | Moderate | Complex | Simple |
| **Single-cell attributes extraction** | No | Few | Average | Few | Many |
| **Visualization Capabilities** | S | S, LT, ALT | S | S | S,LT,GT AS,ALT,  AGT |

*Parameters:*

Too many: ≥ 20 (or the user has to configure an optimal pipeline), Many: ≥ 10, Few: ≤ 5

*Parameterization:*

Complex = Familiarity with image processing required,

Moderate = Some knowledge of image processing required,

Simple = No image processing knowledge is required

*Visualization Capabilities*:

S: Segmentation results

LT: Lineage Tree

DT: Cell Divisions Tree

AS: Attributes of single-cells overlaid on segmented input frame/movie

ALT: Attributes of single-cell over LT

ADT: Life attribute of single-cells over DT

*Single-cell attributes computed:*

Few: Less than 10

Average: Between 10 and 20

Many: Over 20

# Segmentation Results

In additional file 8 we provide the segmentation results (cells detected are outlined by green curvatures) of all the methods when processing the datasets generated by different groups and used in our evaluation (see Materials). The proposed methodology (BaSCA) is robust to different imaging modalities (optical and confocal phase contrast, optical bright field) and a performance that remains consistent with images acquired by different labs.

In Figure S1 below we provide the segmentation results for frame 63 of cell movie SalPhase for which BaSCA exhibited the lowest F-measure.


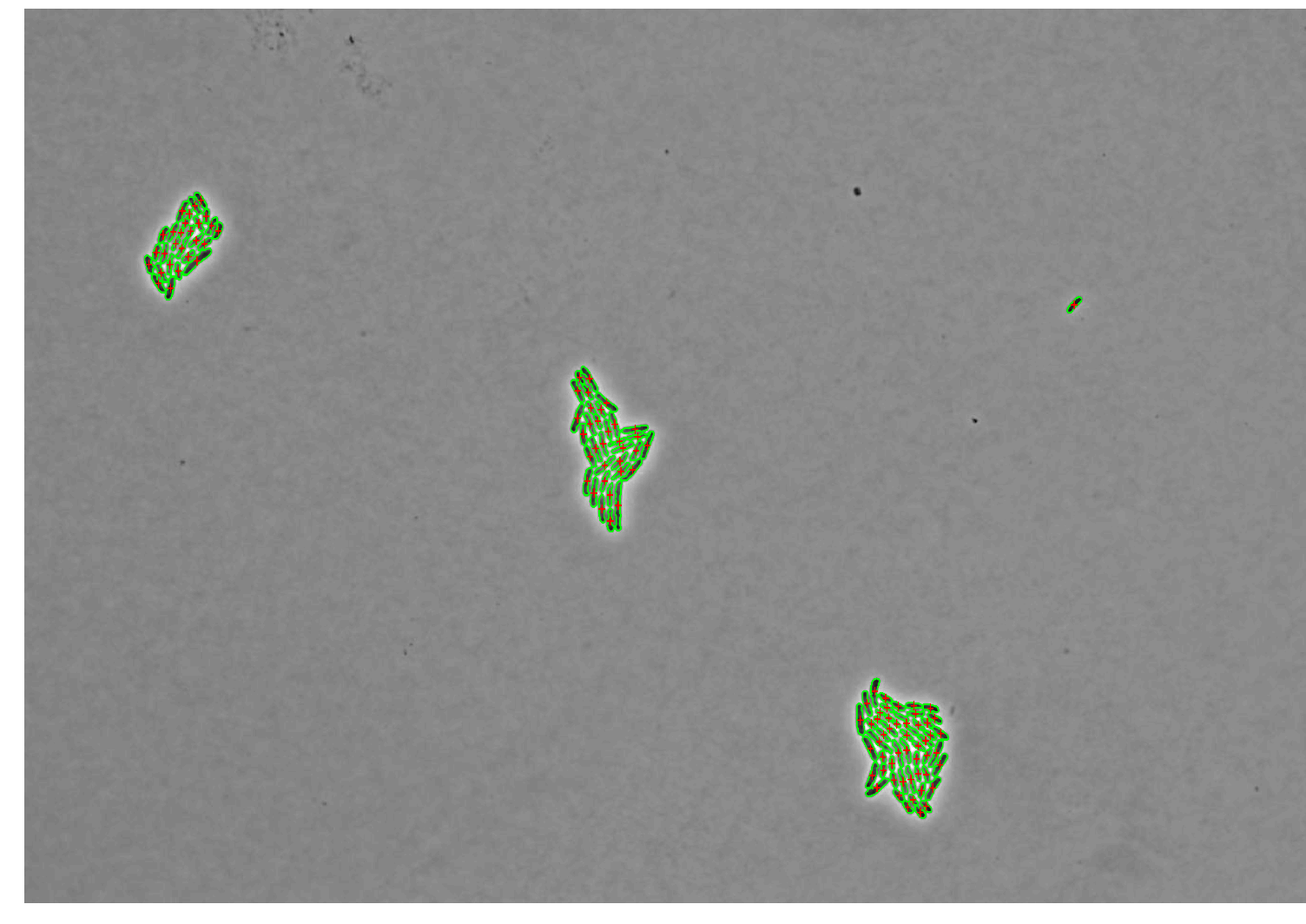


**Figure S1.** BaSCA segmentation results for frame 63 of the SalPhase movie for which it has the lowest F-measure (93.27%) among frames.

# Subpopulations Characterization: Life Attributes Gamma Distributions

The probability density function of a Gamma distribution is given by:

$$\text{f}\left( \text{x;a,b} \right)\text{=}\frac{\text{1}}{\text{Γ(a)b}\text{a}}\text{x}^{\text{a-1}}\text{e}^{\text{-}\frac{\text{x}}{\text{b}}}\text{,}$$

for *x > 0* and *a, b > 0,*  where *Γ(∙)* is the gamma function. We provide below in Table S2 the fitted Gamma model parameters (using maximum likelihood estimate [5]) for the single-cell division time *(T),* elongation rate *(k),* division length *(l*_f_*)* cell life attributes.

**Table S2. Gamma distribution parameters per colony**

| **Colony** | **Division Time** | | **Elongation Rate** | | **Division Length** | |
| --- | --- | --- | --- | --- | --- | --- |
|  | *a* (shape) | *b* (scale) | *a* | *b* | *A* | *b* |
| **1** | 15.801 | 0.052 | 12.379 | 0.062 | 20.647 | 0.229 |
| **2** | 13.322 | 0.057 | 11.101 | 0.071 | 14.303 | 0.358 |
| **3** | 24.384 | 0.033 | 30.308 | 0.028 | 37.829 | 0.118 |

In Figure S2 we provide the histograms of the same attributes for the three colonies of cell movie SalPhase.

**
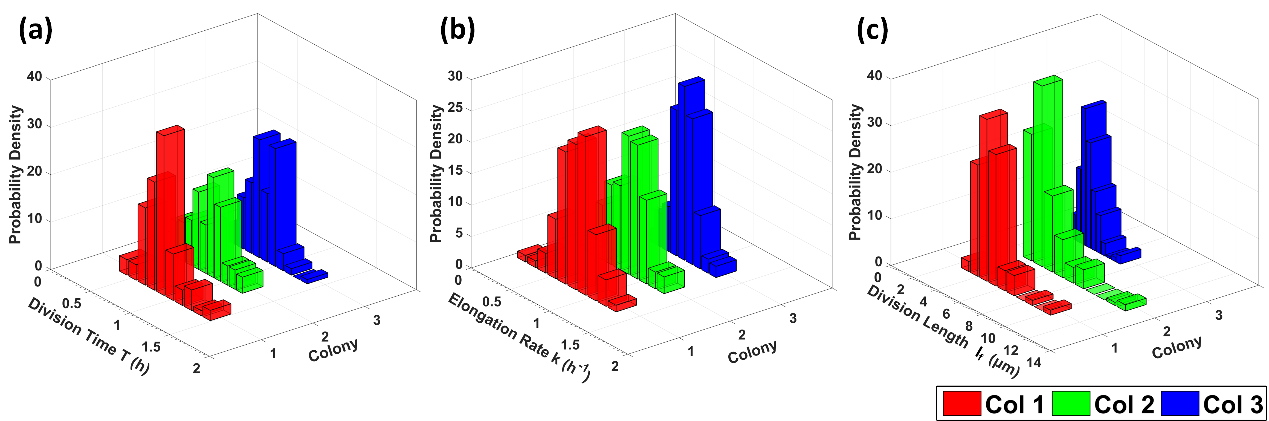
**

**Figure S2. Life attribute histograms per colony.** **(a)** Cell division time *T*, **(b)** Cell elongation rate *k*, **(c)** Cell division length *l_f_* for colonies 1, 2 and 3 (SalPhase movie). The proposed methodology allows us to observe the variability of cell life attributes across colonies.

In Table S3 we provide the best model fit gamma parameters per cell generation for the aforementioned attributes and in Figure S3 the corresponding histograms.

**Table S3. Gamma distribution parameters per cell generation.**

| **Generation** | **Division Time** | | **Elongation Rate** | | **Division Length** | |
| --- | --- | --- | --- | --- | --- | --- |
|  | *a* (shape) | *b* (scale) | *a* | *B* | *a* | *b* |
| 3 | 13.663 | 0.049 | 59.758 | 0.016 | 14.986 | 0.422 |
| 4 | 13.468 | 0.061 | 47.956 | 0.018 | 19.428 | 0.318 |
| 5 | 18.232 | 0.04 | 56.984 | 0.016 | 18.840 | 0.304 |
| 6 | 13.879 | 0.051 | 24.030 | 0.039 | 32.401 | 0.161 |
| 7 | 21.828 | 0.038 | 13.967 | 0.049 | 39.067 | 0.114 |
| 8 | 25.203 | 0.032 | 31.740 | 0.027 | 68.741 | 0.058 |

**
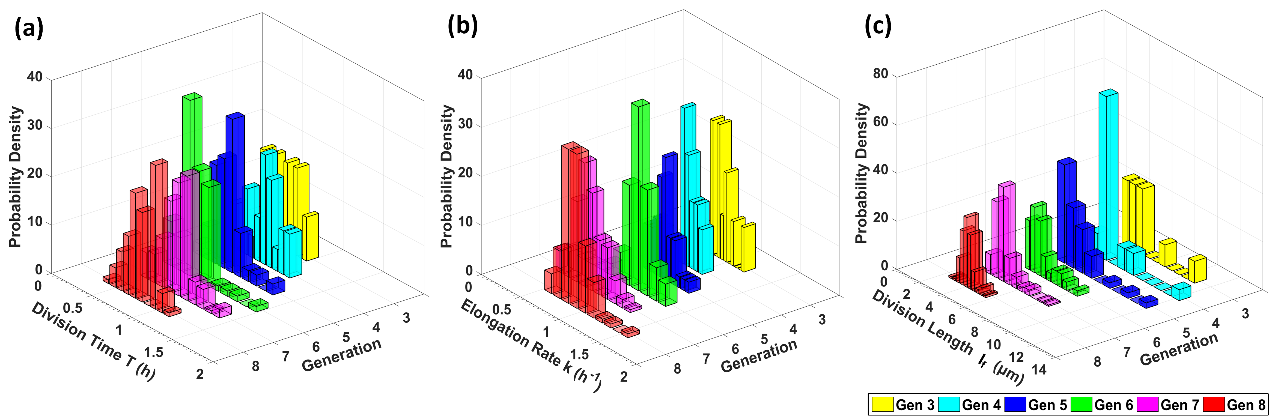
**

**Figure S3. Life attribute histograms per cell generation.** **(a)** Cells division time *T*, **(b)** Cell elongation rate *k*, and **(c)** Cell length at division *l_f_*, for the 3^rd^ to the 8^th^ generation of cells of the SalPhase movie (cells from all colonies are pooled). By delving into each generation’s individuals we can observe a life attribute's intra and inter-generation variability (stochasticity).

# Single-cell life Attribute Scatterplots


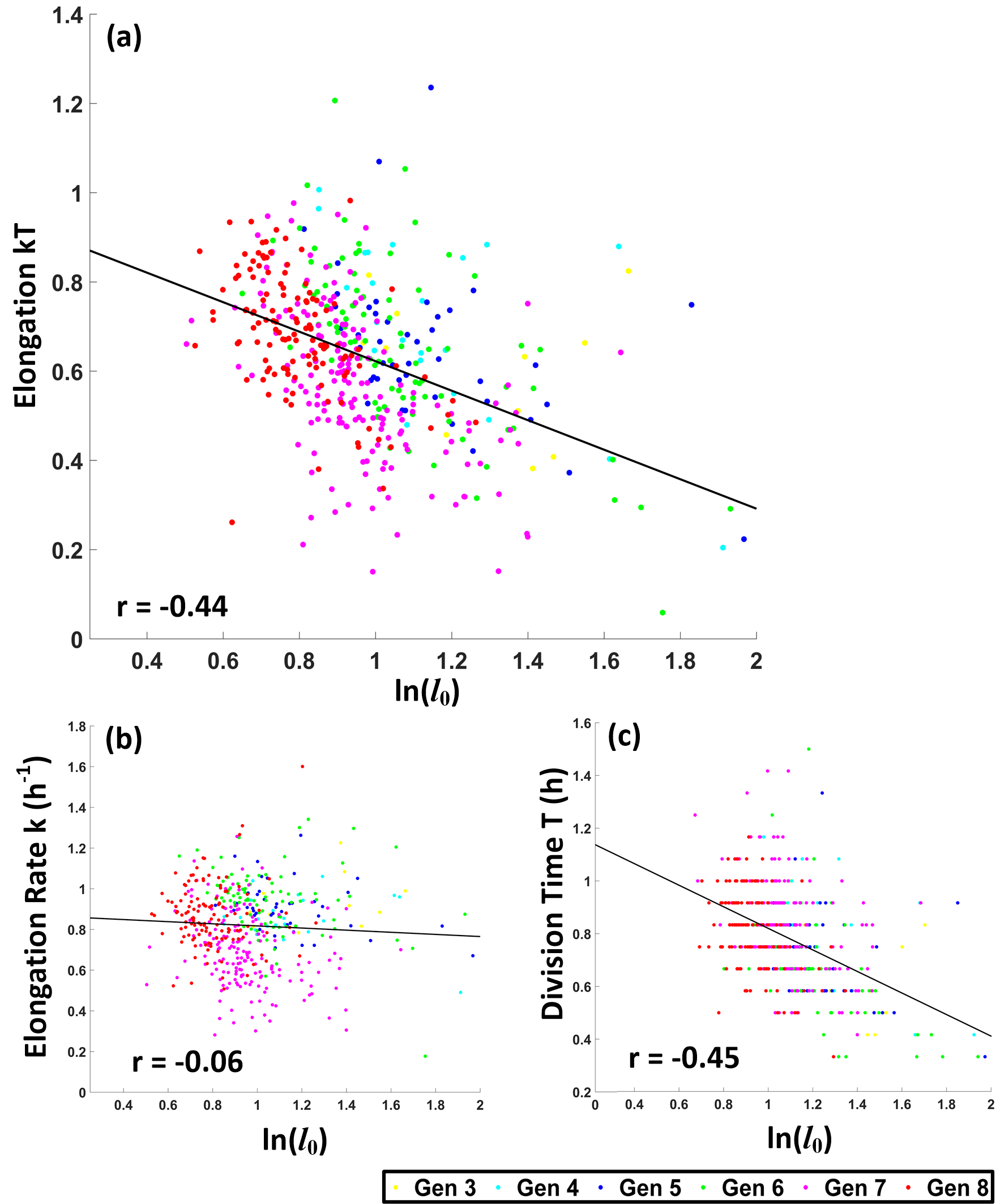


**Figure S4. Correlations of life attributes (SalPhase movie).** **(a)** The logarithm of the cell birth length $\ln\left( \text{l}_{\text{0}} \right)$ and cellular elongation *kT* are anti-correlated (Pearson correlation **≈** -0.44). **(b)** The single-cell growth rate is not correlated with birth length (Pearson correlation **≈** 0). **(c)** The elongation and birth length relation is completely accounted for by the anti-correlation of birth length and division time (Pearson correlation **≈** -0.45).

# Running time

Segmenting a time lapse cell movie of growing bacterial colonies is a very time consuming task with running time growing fast with the number of frames in the movie (see Fig. S5(a)). This is so because the time for segmenting each frame grows as time progresses, since the number of cells per frame grow exponentially (see Fig. S5(b) for the SalPhase movie). However, due to the careful design of the pipeline's algorithms the total running time of BaSCA grows only linearly with the number of cells in a frame as shown in Fig. S5(c). Therefore, the computational cost per cell tends to a constant value as shown in Fig. S5(d). We would like to remark that the results of Figure S5 are obtained using the currently non-optimized version of the BaSCA Matlab code that does not exploit any parallelism at all. This constant value (time per cell) will be reduced significantly when we start exploiting the ample of parallelism available at the colony and the object levels due to the divide-and-conquer processing approach we are following deliberately in BaSCA which breaks down the large problem (segmenting overcrowded colonies in dense frames) into many independent small problems. This is an optimization we plan to complete before the first release of BaSCA's implementation.


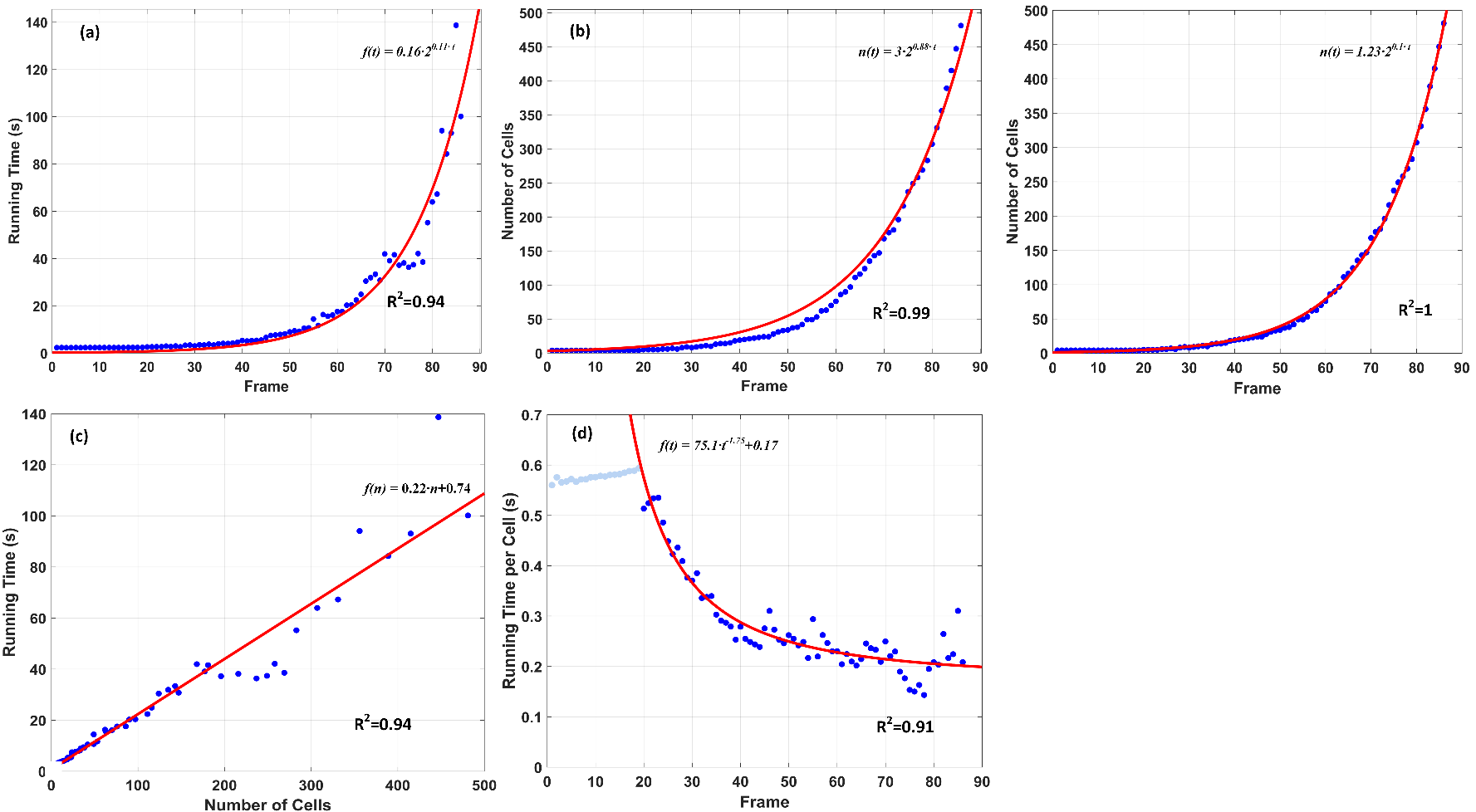


**Figure S5. Running time (**SalPhase movie): **(a)** The running time increases exponentially with time (frame index) as does the number of cells (best fit model is provided by the solid red curve). (b) Exponential growth of number of cells with time. **(c)** Running time increases linearly with the number of cells in the frame. **(d)** The computational cost per extracted cell converges to a plateau. Currently the parallelism available is not exploited.

# References

1. Wang Q, You L, West M. CellTracer 1.0. 2008.

<https://stat.duke.edu/research/software/west/celltracer/>.Accessed 03 May 2016.

1. Klein J. TLM-Tracker. 2012.

<http://www.tlmtracker.tu-bs.de/index.php/Main_Page/>. Accessed 03 May 2016.

1. Young JW, Locke JC, Altinok A, Rosenfeld N, Bacarian T, Swain PS, Mjolsness E, Alon U, Elowitz MB. Schnitzcells. 2011.

<http://easerver.caltech.edu/wordpress/schnitzcells/>. Accessed 03 May 2016.

1. Paintdakhi A, Parry B, Campos M, Irnov I, Elf J, Surovtsev I, Jacobs-Wagner C. Oufti. 2016. <http://oufti.org/>.Accessed 03 May 2016.
2. Theodoridis S, Koutroumbas K. *Machine Learning*, A Bayesian and Optimization Perspective. 1st ed. USA: Elsevier, 2015.
