# Supplementary material for: Image analysis driven single-cell analytics for systems microbiology
Source: BMC Syst Biol. 2017 Apr 4;11:43. doi: 10.1186/s12918-017-0399-z (PMC5379763; doi:10.1186/s12918-017-0399-z)
Supplement: Supplementary file 7 — Contains for each dataset the segmentation results of each method (.tif images) and corresponding parameterization files (.mat files). (ZIP 62299 kb) [file 12918_2017_399_MOESM7_ESM.zip › additional file 8/README.pdf]

README FILE

A reader can produce the segmentation results by using the datasets described in Materials (images enclosed in both in uint8 and uint16 format) and the provided parameterization files (Matlab [1] .mat file). For each dataset (tested image) all relevant files for each software are included in the respective directory, along with the produced segmentation results.

The steps to follow to reproduce the simulation results are described below:

1) Download the software packages source code from the corresponding sites (see below).
2) Open Matlab.
3) Make Matlab's current directory to be the directory of a software's source code.
4) Add from the Matlab environment the path so as to include all sub directories of source code folder.
5) Initialize code according to each software's tutorial,
    a. Provide the path of the input image.
    b. Load the corresponding .mat file with the parameterization (when required).
6) Run segmentation according to each tool as discussed next.

Specifically, for each tool:

For CellTracer (version 1.0.3) [2] we enclose the segmentation results for their dataset (in .tif file format) which are provided in CellTracer's webpage [3] along with the software source code, thus no parameterization file is included. One, in order to reproduce the results, should follow the steps provided in CellTracer's webpage [3]. We did not provide segmentation results for the rest of the images because the program failed to return results for them.

For TLM-Tracker [4] we used its default pipeline arrangement and the parameterization file "watersheds_phasecontrast.mat" available at the TLM-Tracker website, for analyzing phase contrast images (for all the images except for TLM-Tracker's image and Microbe Tracker's image). This parameterization file was found to produce the best segmentation results for all datasets. The default pipeline arrangement and parameterization (.mat file) as well as the source code of the package can be found in [5]. In order to reproduce the results, the image to test should be loaded from TLM-Tracker's GUI along with the parameterization file "watersheds_phasecontrast.mat". In the case of TLM-Tracker's sample image we do not use the aforementioned parameterization file, but we obtain the results for their sample image available at their website. For Microbe Tracker's image, in order to reproduce the results we obtained, the image should be loaded from TLM-Tracker's GUI along with the parameterization file

"MicrobeTracker_TL1_2min_2hrs_St1_Phase_028_TLM-Tracker.mat".

For Oufti [8] we include the image under evaluation along with a .mat file with the optimized parameterization. The source code of the package can be found in Oufti's website [9]. For further details on how to use Oufti a tutorial is available in [9]. Except for the source code, the developers of Oufti provide a compiled version (Matlab independent) of the software working in 64-bit operating systems only and it is available for download from [9]. In order to reproduce our results, one should load an image along with the corresponding parameterization file (.mat files in every image related folder).

For Schnitzcells (version 1.1) [6], in addition to the evaluated image, we include a .mat file with the parameterization exhibiting the best result. The source code of the package can be found in Schnitzcells' website [7]. We remark that Schnitzcells requires the input images to be strictly in uint16 format while the rest of the software packages can handle images in formats uint8 or uint16. In order to run Schnitzcells in Matlab after steps 1-4 (as described above) we need to execute the following steps:

1) Initialization:

a) Run initialization:

```
s = initschnitz('movieName', '2016-11-19', 'bacillus', 'rootDir',...
        '.\directoryName','imageDir','.\directoryName');
```

movieName: the name of the movie to load. For the tested images use the name of the image until the "unit16", e.g. for the image "CellTracer_VNmovie_uint16-p-024.tif", use "CellTracer_VNmovie_uint16" argument as ***movieName***.

directoryName: insert a name (whatever name you want) for the directory to be created for saving the results. Once this directory is created put inside the image to be tested and continue.

b) Load the corresponding parameterization .mat file in Matlab's workspace. A *struct* with name *p* is going to be imported.

2) Run segmentation:

```
s = segmoviephase(s, 'segmentationPhaseSlice', 1, 'segRange', nFrame, 'minCellArea', p.minCellArea, 'edge_lapofgauss_sigma', p.edge_lapofgauss_sigma, 'minCellLengthConservative', p.minCellLengthConservative, 'minCellLength', p.minCellLength, 'maxCellWidth', p.maxCellWidth,'maxThreshCut',p.maxThreshCut, 'maxThreshCut2', p.maxThreshCut2, 'maxThresh', p.maxThresh, 'minThresh', p.minThresh, 'radius', p.radius, 'angThresh', p. angThresh);
```

Please note that the input argument ***segRange***, ***nFrame***, corresponds to the frame number of the tested image, shown in the image filename just before the extension, e.g. for the "CellTracer_VNmovie_uint16-p-024.tif" image the ***nFrame*** should be set to 24. The results are saved in the corresponding subdirectory of the ***directoryName.***

References

[1] MATLAB Release R2015b, The MathWorks, Inc., Natick, Massachusetts, United States.

[2] Wang Q, Niemi J, Tan CM, You L, West M: Image segmentation and dynamic lineage analysis in single-cell fluorescence microscopy. Cytometry A. 2010, 77:101-110.

[3] Wang Q, You L, West M. CellTracer 1.0. 2008. https://stat.duke.edu/research/software/west/celltracer/. Accessed 03 May 2016.

[4] Klein J, Leupold S, Biegler I, Biedendieck R, Münch R, Jahn D: TLM-Tracker: software for cell segmentation, tracking and lineage analysis in time-lapse microscopy movies. Bioinformatics 2012, 28:2276-2277.

[5] Klein J. TLM-Tracker. 2012. http://www.tlmtracker.tu-bs.de/index.php/Main_Page. Accessed 03 May 2016.

[6] Young JW, Locke JC, Altinok A, Rosenfeld N, Bacarian T, Swain PS, Mjolsness E, Elowitz MB: Measuring single-cell gene expression dynamics in bacteria using fluorescence time-lapse microscopy. Nat Protoc 2011, 7:80-88.

[7] Young JW, Locke JC, Altinok A, Rosenfeld N, Bacarian T, Swain PS, Mjolsness E, Alon U, Elowitz MB. Schnitzcells. 2011. http://easerver.caltech.edu/wordpress/schnitzcells/. Accessed 03 May 2016.

[8] Paintdakhi A, Parry B, Campos M, Irnov I, Elf J, Surovtsev I, Jacobs-Wagner C: Oufti: An integrated software package for high-accuracy, high-throughput quantitative microscopy analysis. Mol Microbiol 2015, 99:767-777.

[9] Paintdakhi A, Parry B, Campos M, Irnov I, Elf J, Surovtsev I, Jacobs-Wagner C. Oufti. 2016. http://oufti.org/. Accessed 03 May 2016.
