# Supplementary figures and images for: Image analysis driven single-cell analytics for systems microbiology
Source: BMC Syst Biol. 2017 Apr 4;11:43. doi: 10.1186/s12918-017-0399-z (PMC5379763; doi:10.1186/s12918-017-0399-z)

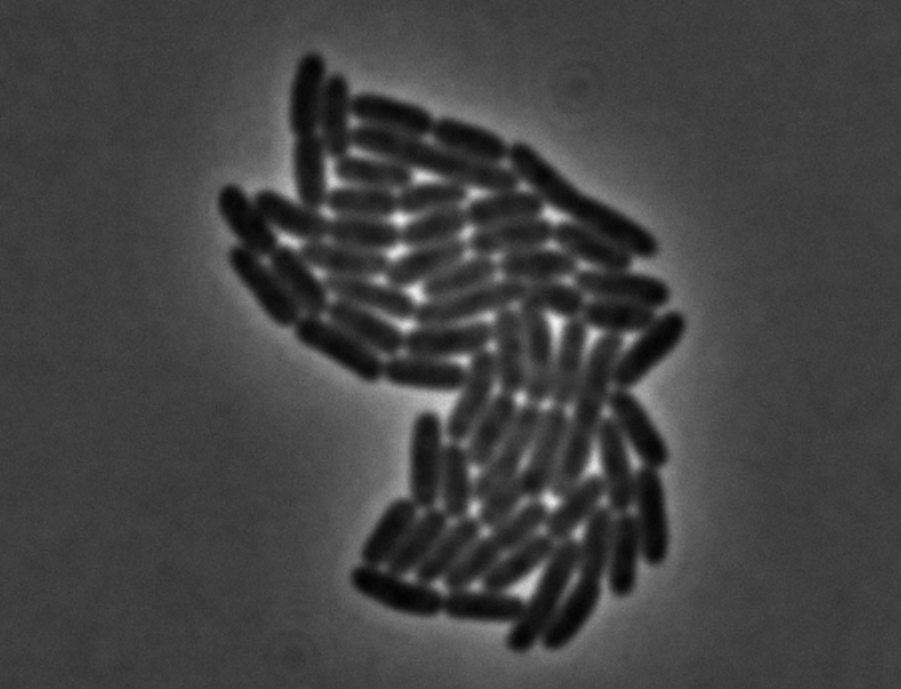

Supplement: Supplementary file 7 — Contains for each dataset the segmentation results of each method (.tif images) and corresponding parameterization files (.mat files). (ZIP 62299 kb) [file 12918_2017_399_MOESM7_ESM.zip › additional file 8/CellTracer image/CellTracer_VNmovie-p-024.tif]

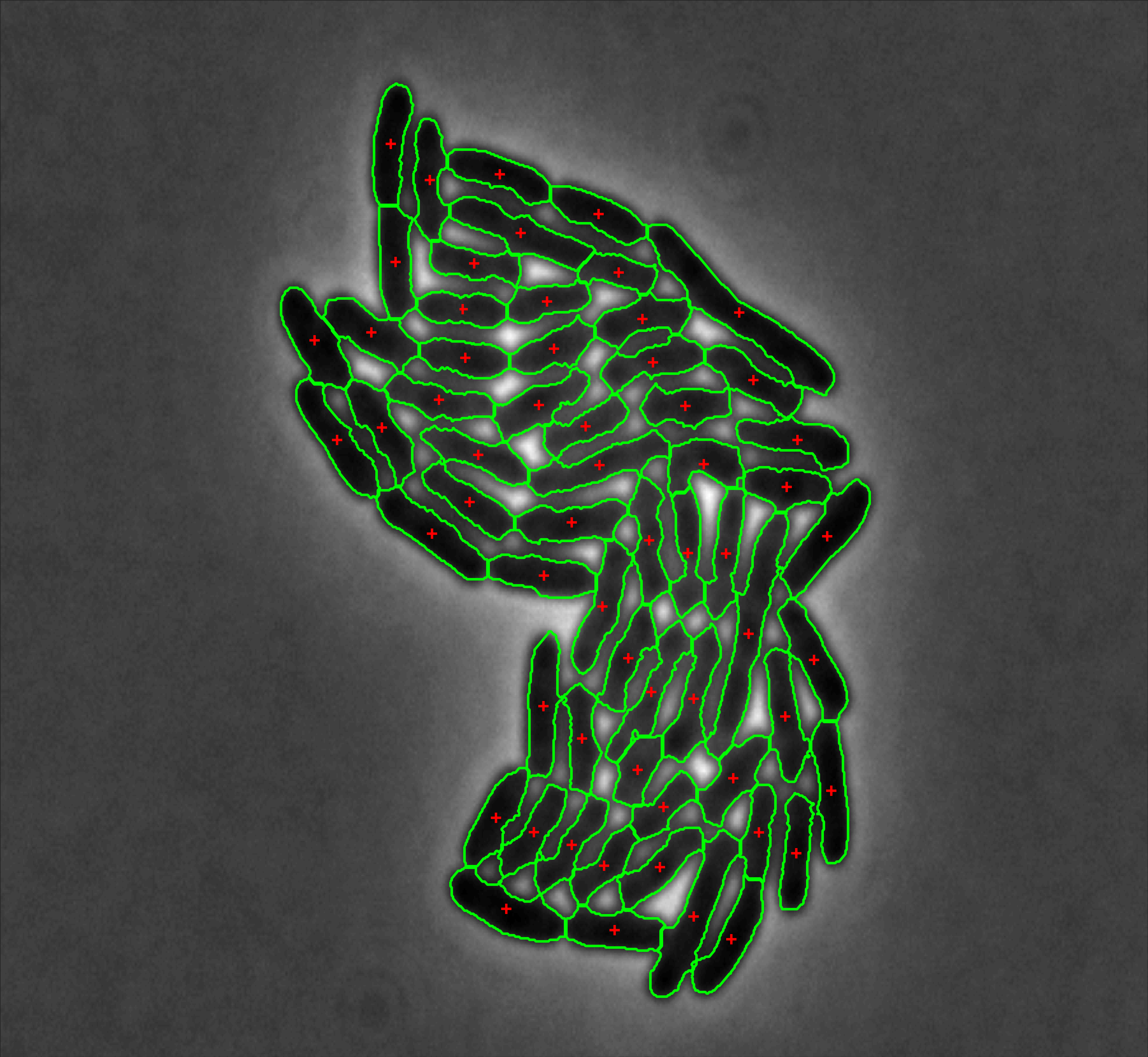

Supplement: Supplementary file 7 — Contains for each dataset the segmentation results of each method (.tif images) and corresponding parameterization files (.mat files). (ZIP 62299 kb) [file 12918_2017_399_MOESM7_ESM.zip › additional file 8/CellTracer image/CellTracer_VNmovie-p-024_BaSCA.tif]

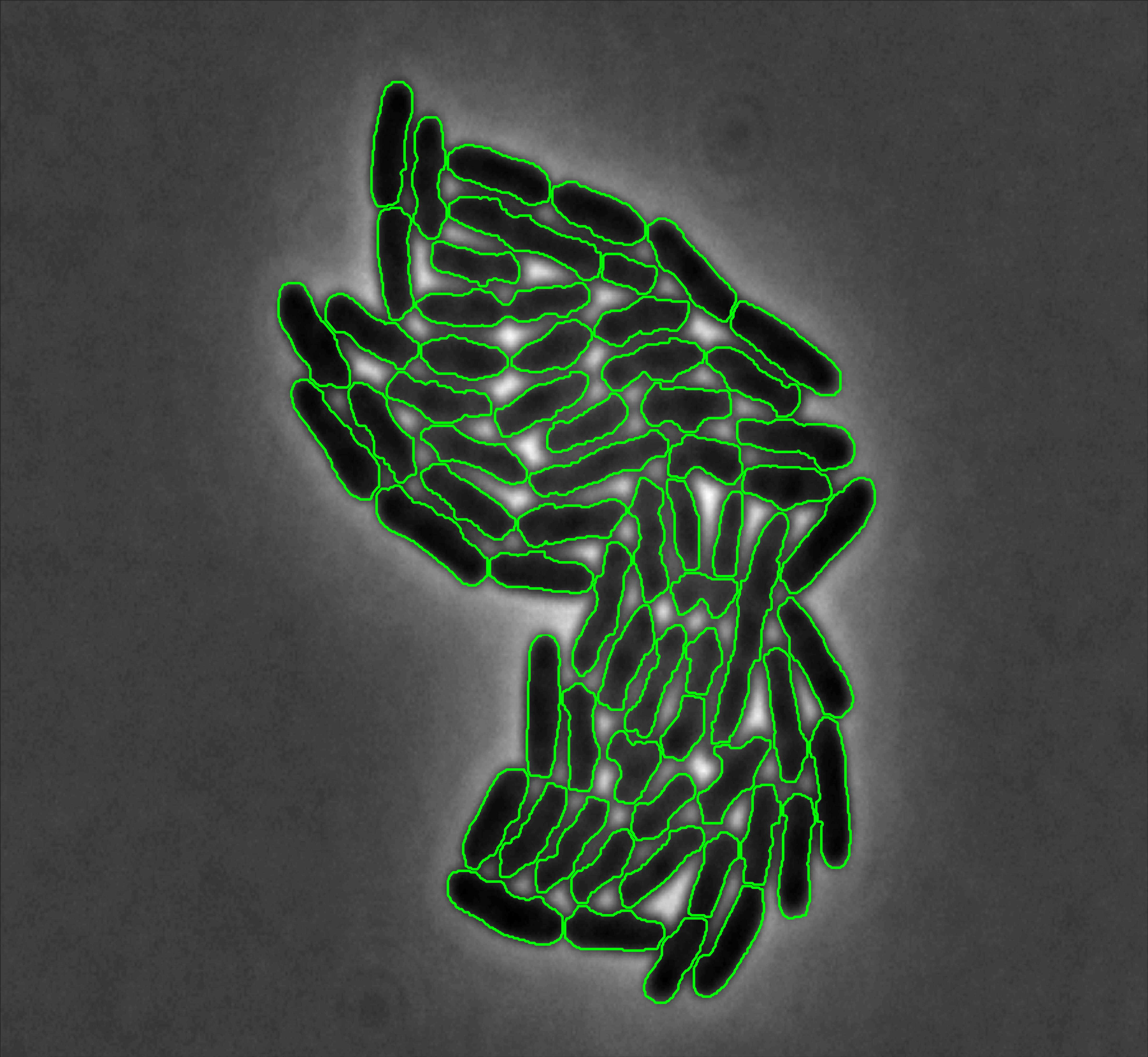

Supplement: Supplementary file 7 — Contains for each dataset the segmentation results of each method (.tif images) and corresponding parameterization files (.mat files). (ZIP 62299 kb) [file 12918_2017_399_MOESM7_ESM.zip › additional file 8/CellTracer image/CellTracer_VNmovie-p-024_CellTracer.tif]

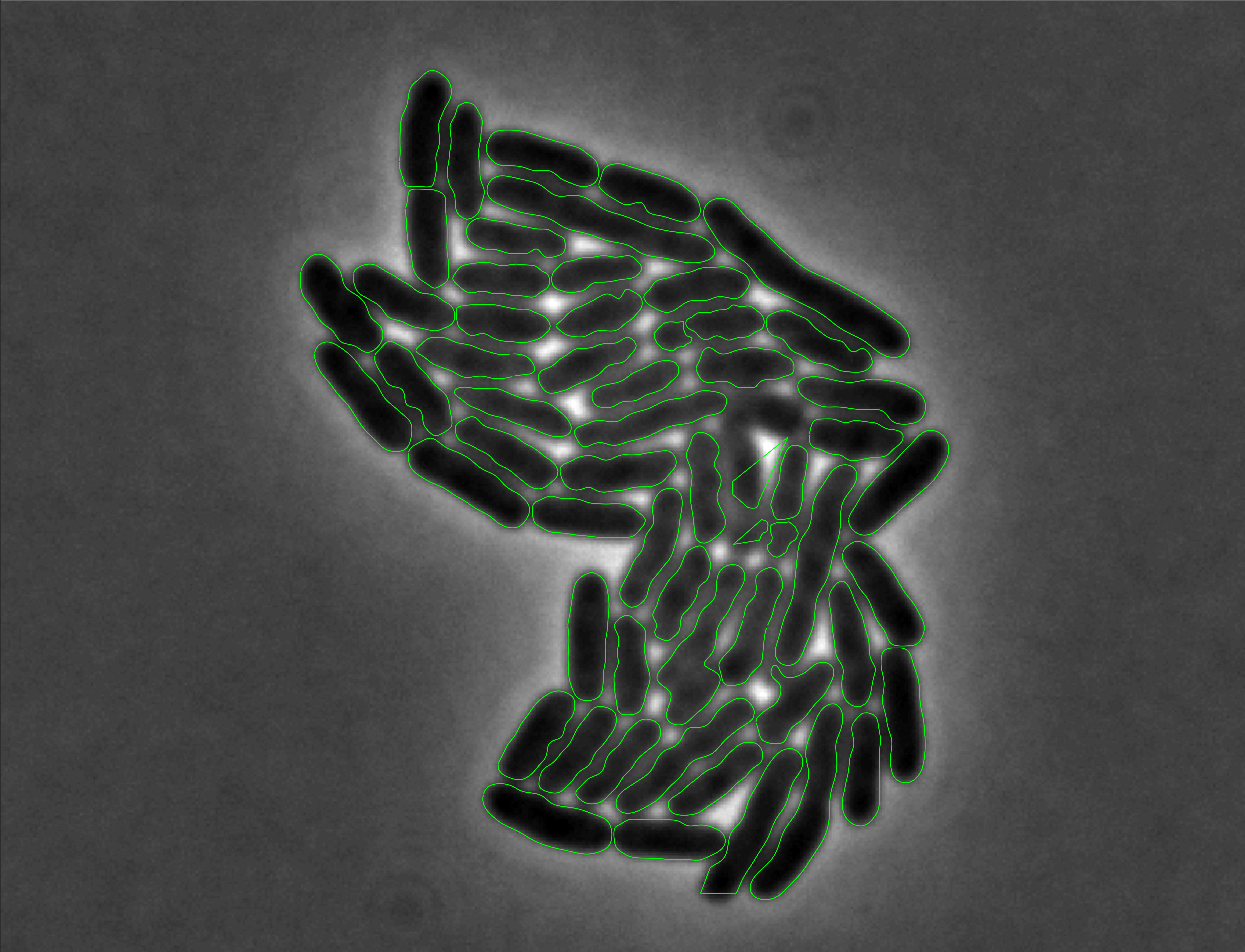

Supplement: Supplementary file 7 — Contains for each dataset the segmentation results of each method (.tif images) and corresponding parameterization files (.mat files). (ZIP 62299 kb) [file 12918_2017_399_MOESM7_ESM.zip › additional file 8/CellTracer image/CellTracer_VNmovie-p-024_Oufti.tif]

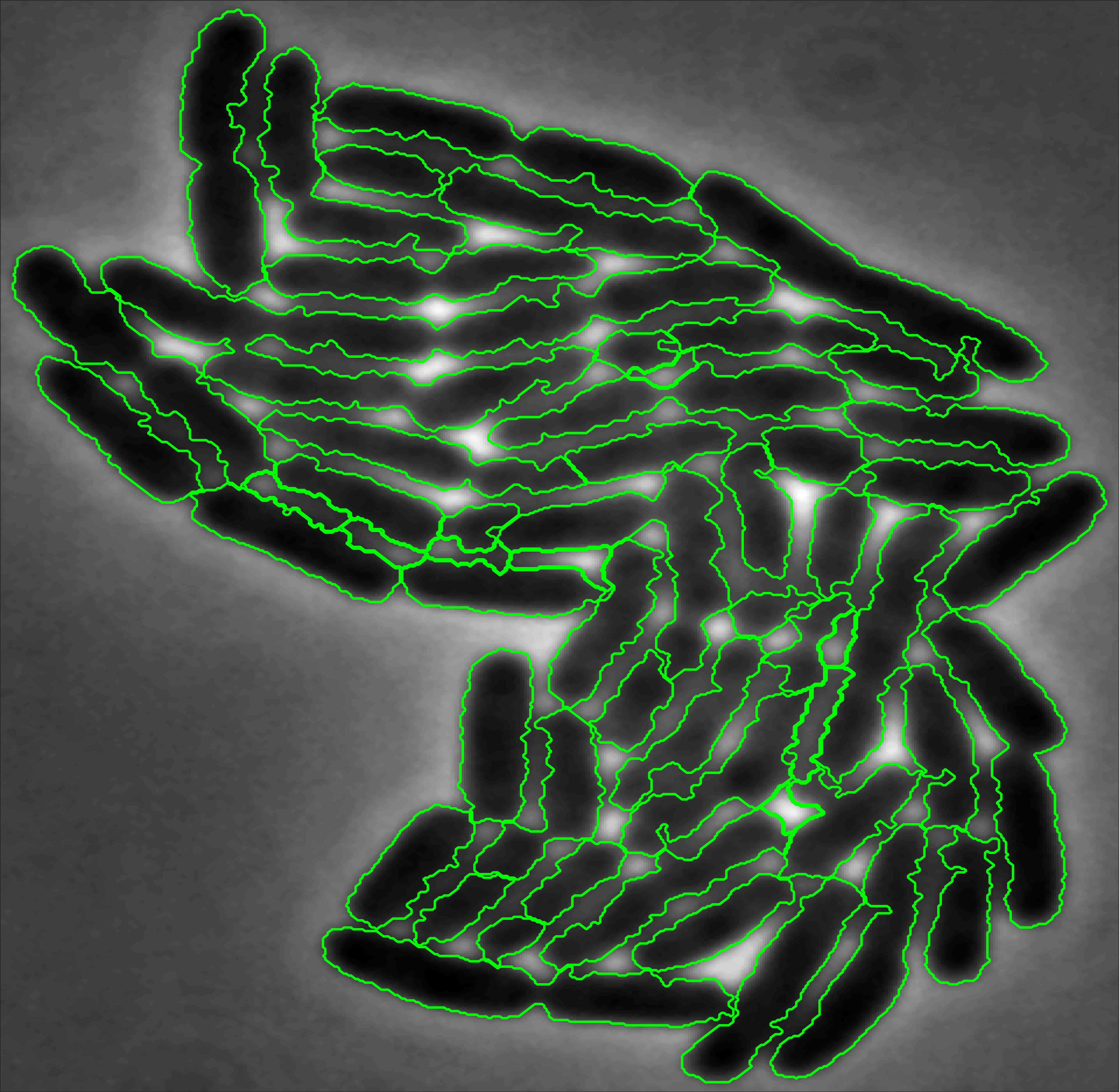

Supplement: Supplementary file 7 — Contains for each dataset the segmentation results of each method (.tif images) and corresponding parameterization files (.mat files). (ZIP 62299 kb) [file 12918_2017_399_MOESM7_ESM.zip › additional file 8/CellTracer image/CellTracer_VNmovie-p-024_Schnitzcells.tif]

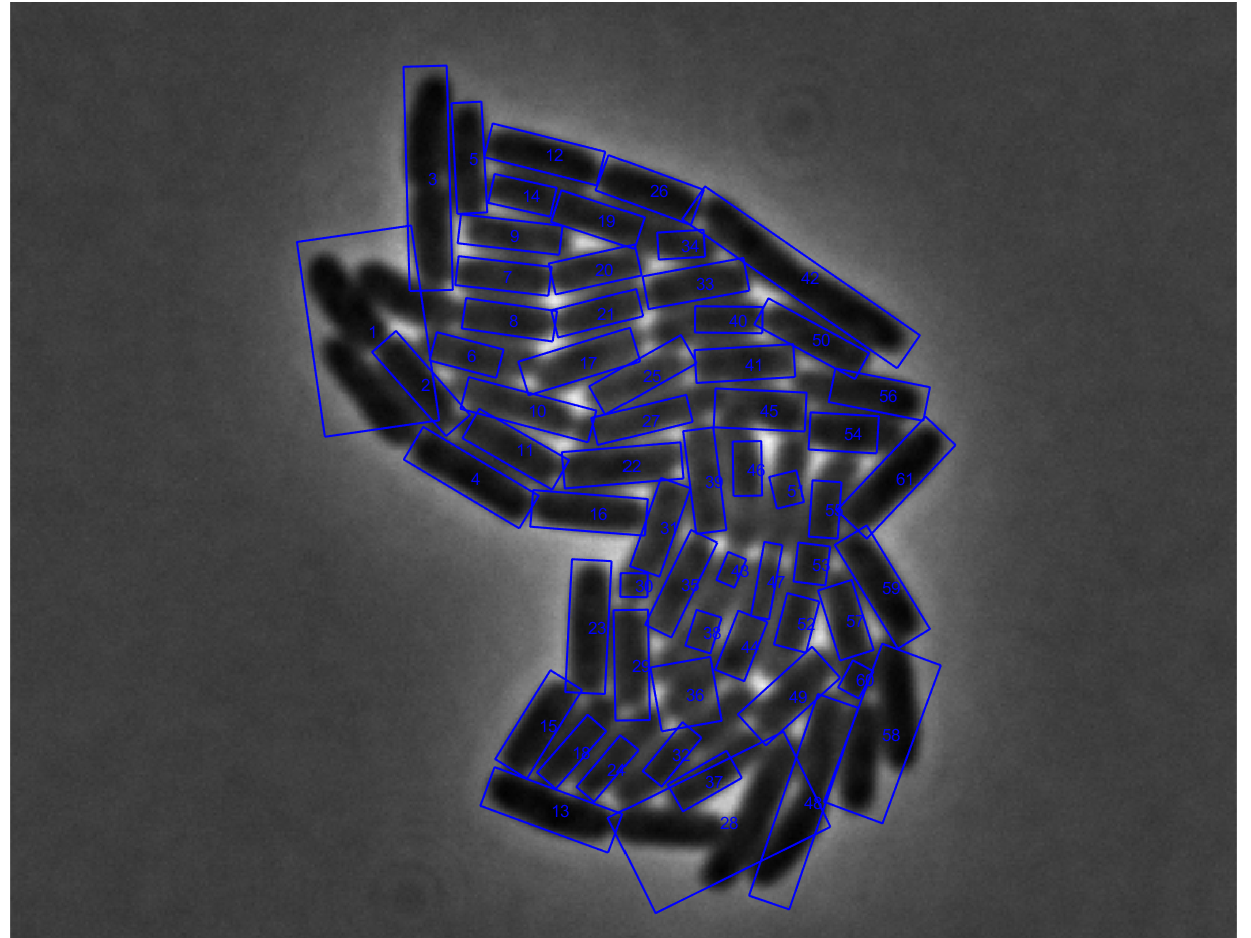

Supplement: Supplementary file 7 — Contains for each dataset the segmentation results of each method (.tif images) and corresponding parameterization files (.mat files). (ZIP 62299 kb) [file 12918_2017_399_MOESM7_ESM.zip › additional file 8/CellTracer image/CellTracer_VNmovie-p-024_TLM-Tracker.tif]

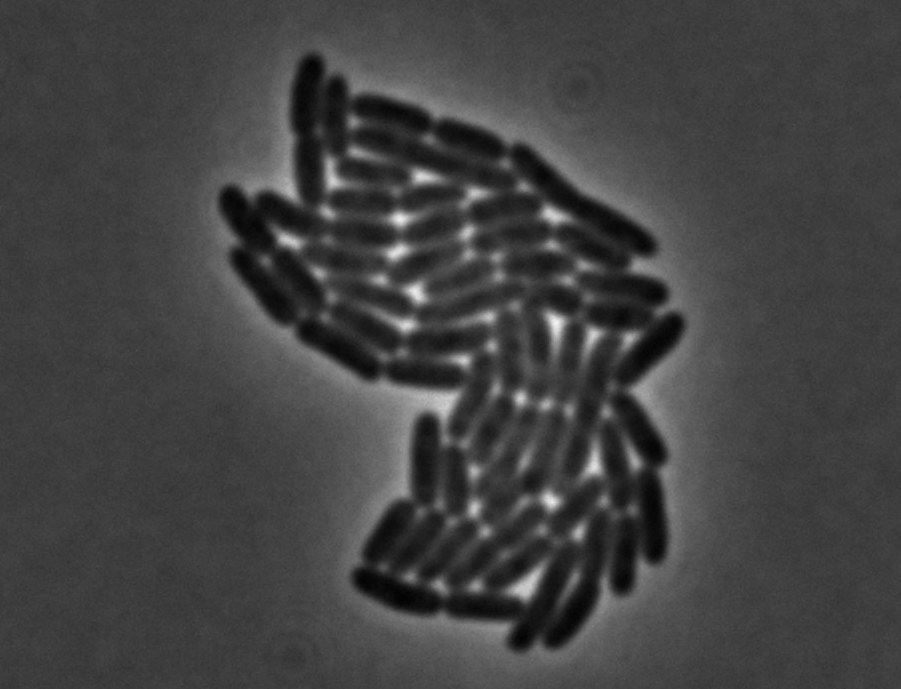

Supplement: Supplementary file 7 — Contains for each dataset the segmentation results of each method (.tif images) and corresponding parameterization files (.mat files). (ZIP 62299 kb) [file 12918_2017_399_MOESM7_ESM.zip › additional file 8/CellTracer image/CellTracer_VNmovie_uint16-p-024.tif]

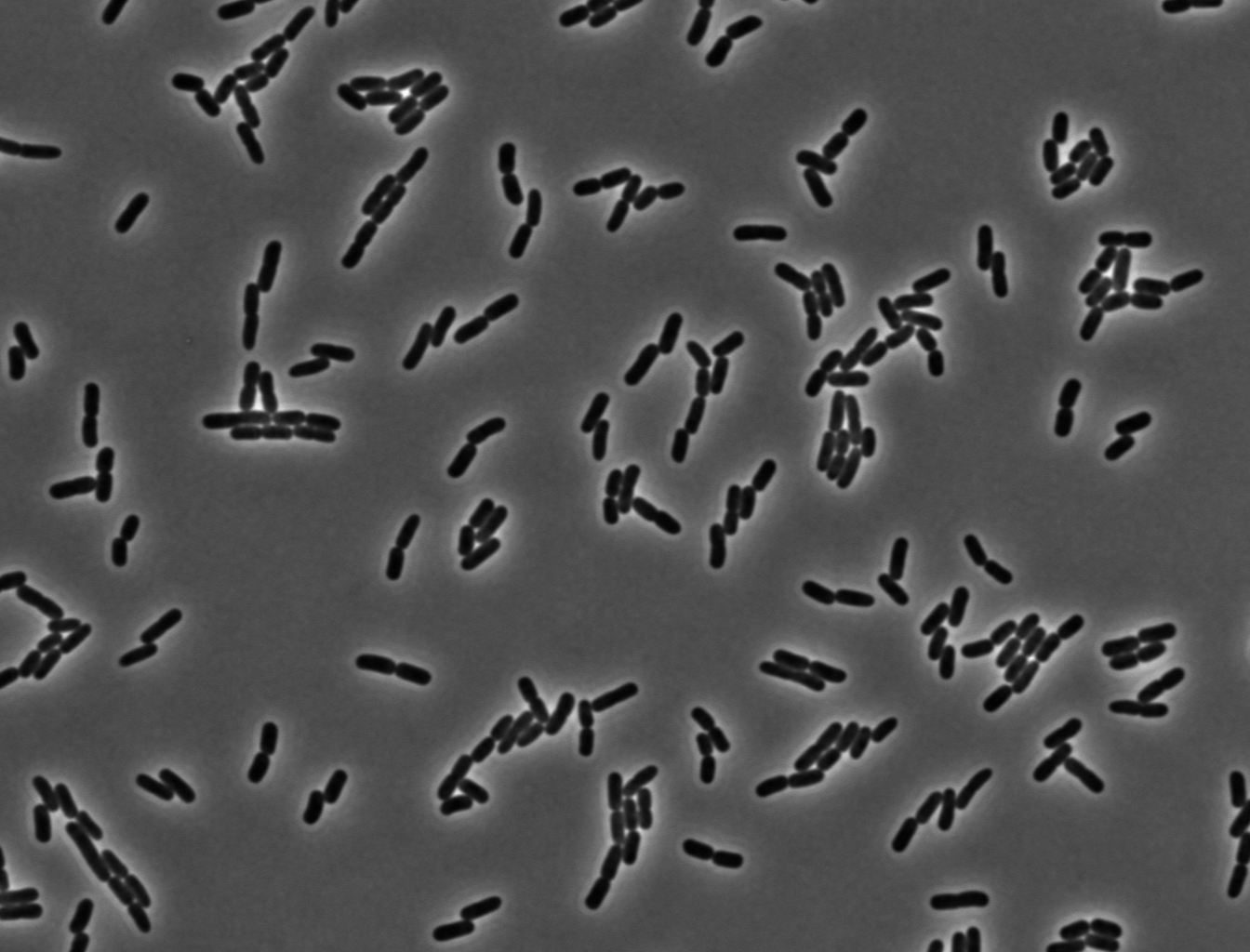

Supplement: Supplementary file 7 — Contains for each dataset the segmentation results of each method (.tif images) and corresponding parameterization files (.mat files). (ZIP 62299 kb) [file 12918_2017_399_MOESM7_ESM.zip › additional file 8/MT image/MicrobeTracker_TL1_2min_2hrs_St1-p-028.tif]

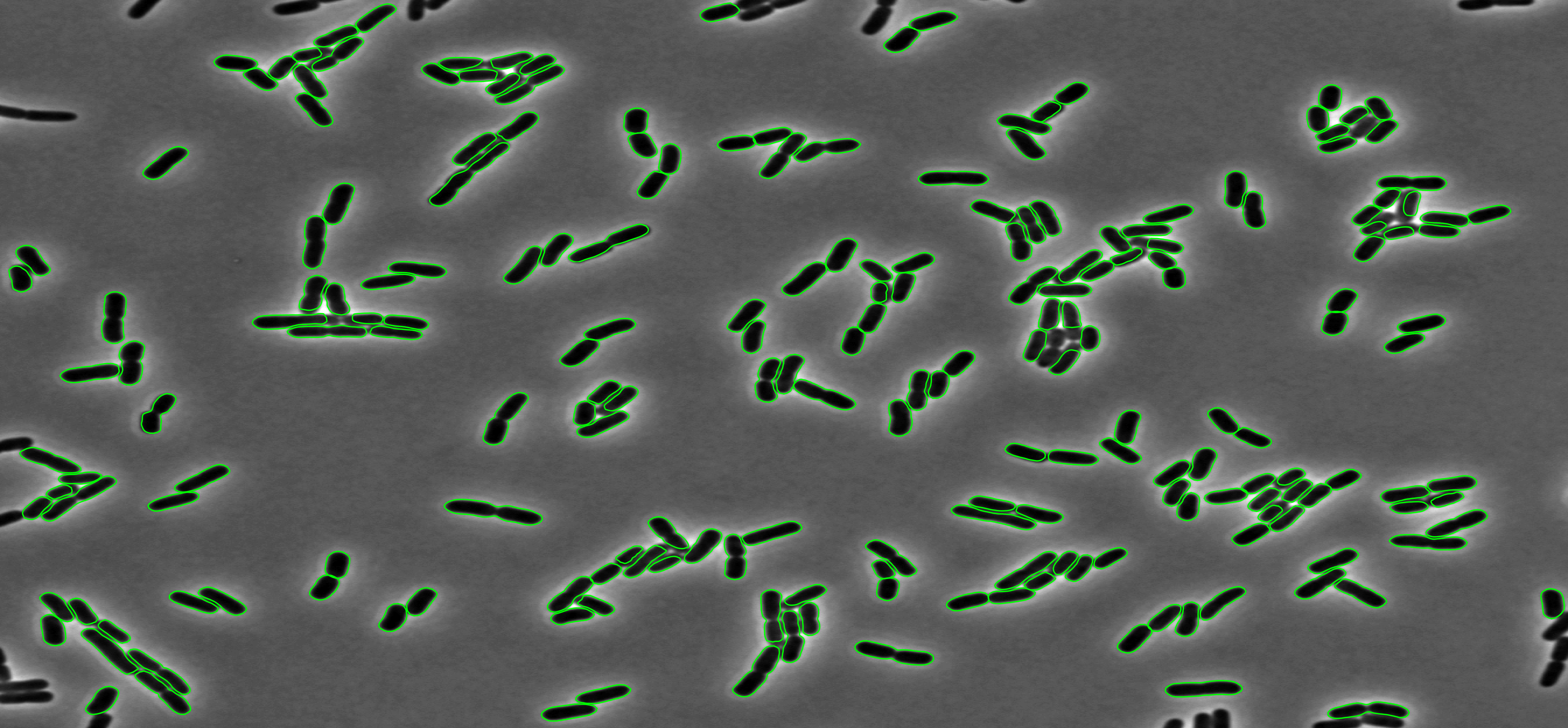

Supplement: Supplementary file 7 — Contains for each dataset the segmentation results of each method (.tif images) and corresponding parameterization files (.mat files). (ZIP 62299 kb) [file 12918_2017_399_MOESM7_ESM.zip › additional file 8/MT image/MicrobeTracker_TL1_2min_2hrs_St1-p-028_Oufti.tif]

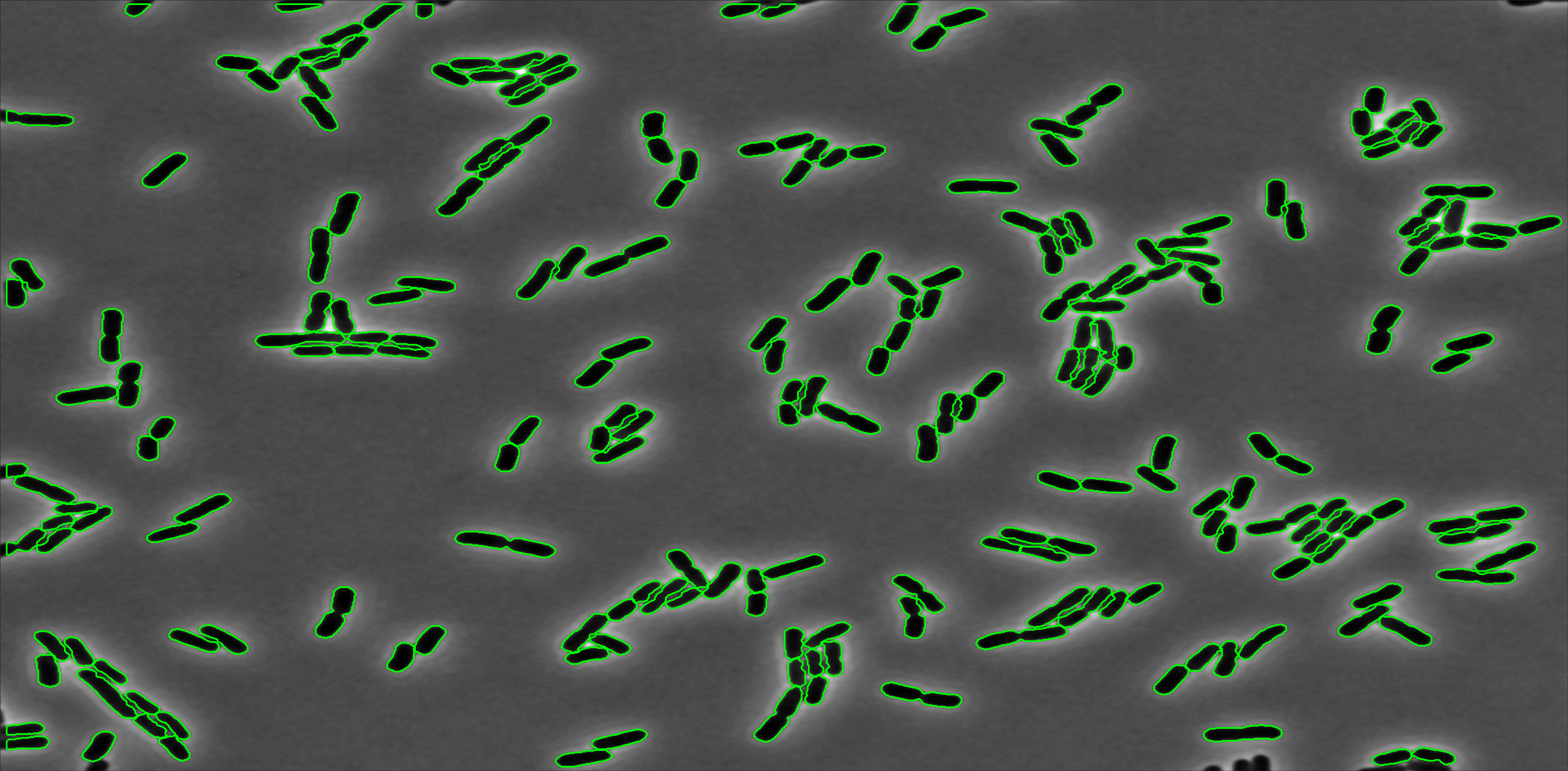

Supplement: Supplementary file 7 — Contains for each dataset the segmentation results of each method (.tif images) and corresponding parameterization files (.mat files). (ZIP 62299 kb) [file 12918_2017_399_MOESM7_ESM.zip › additional file 8/MT image/MicrobeTracker_TL1_2min_2hrs_St1-p-028_Schnitzcells.tif]

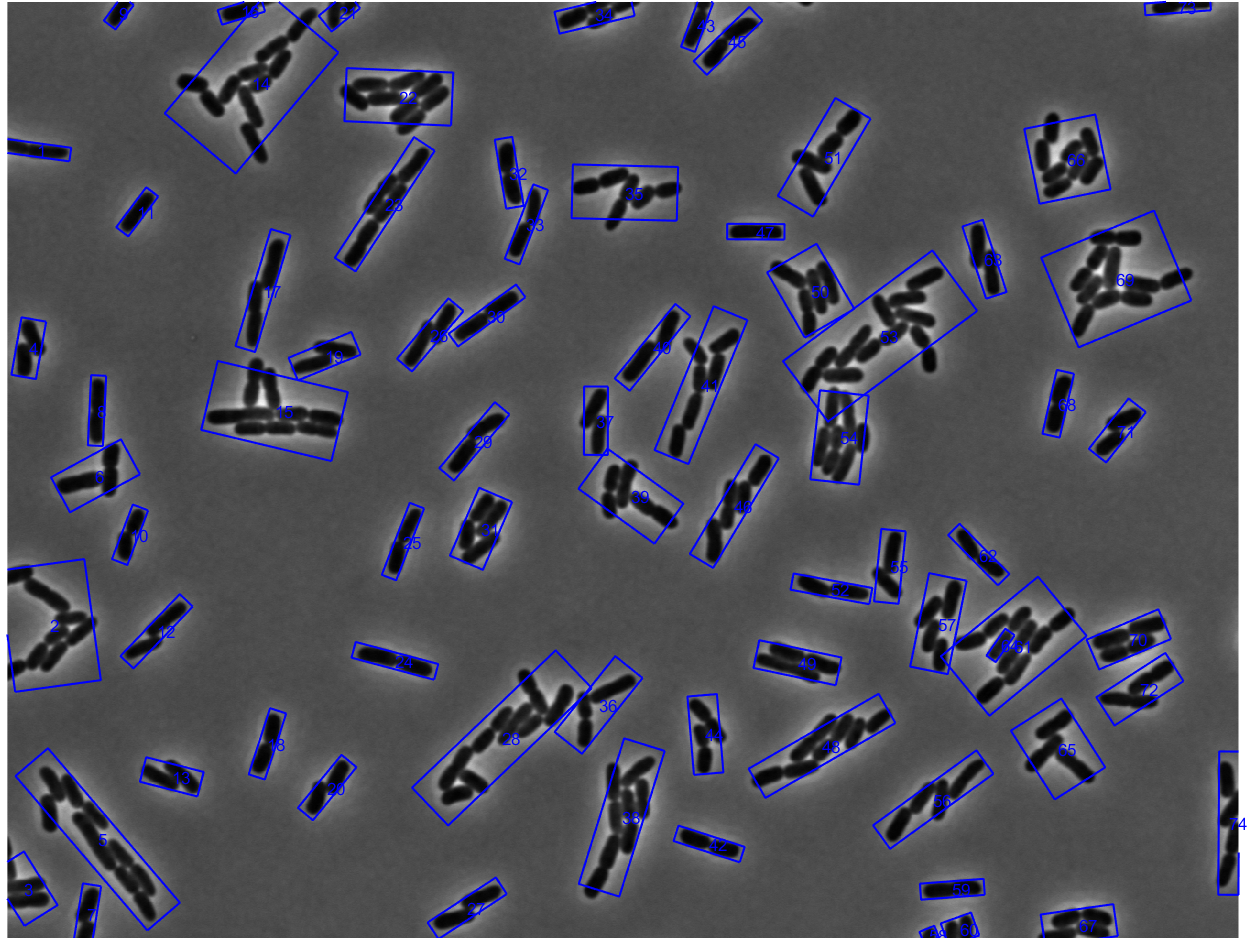

Supplement: Supplementary file 7 — Contains for each dataset the segmentation results of each method (.tif images) and corresponding parameterization files (.mat files). (ZIP 62299 kb) [file 12918_2017_399_MOESM7_ESM.zip › additional file 8/MT image/MicrobeTracker_TL1_2min_2hrs_St1-p-028_TLM-Tracker.tif]

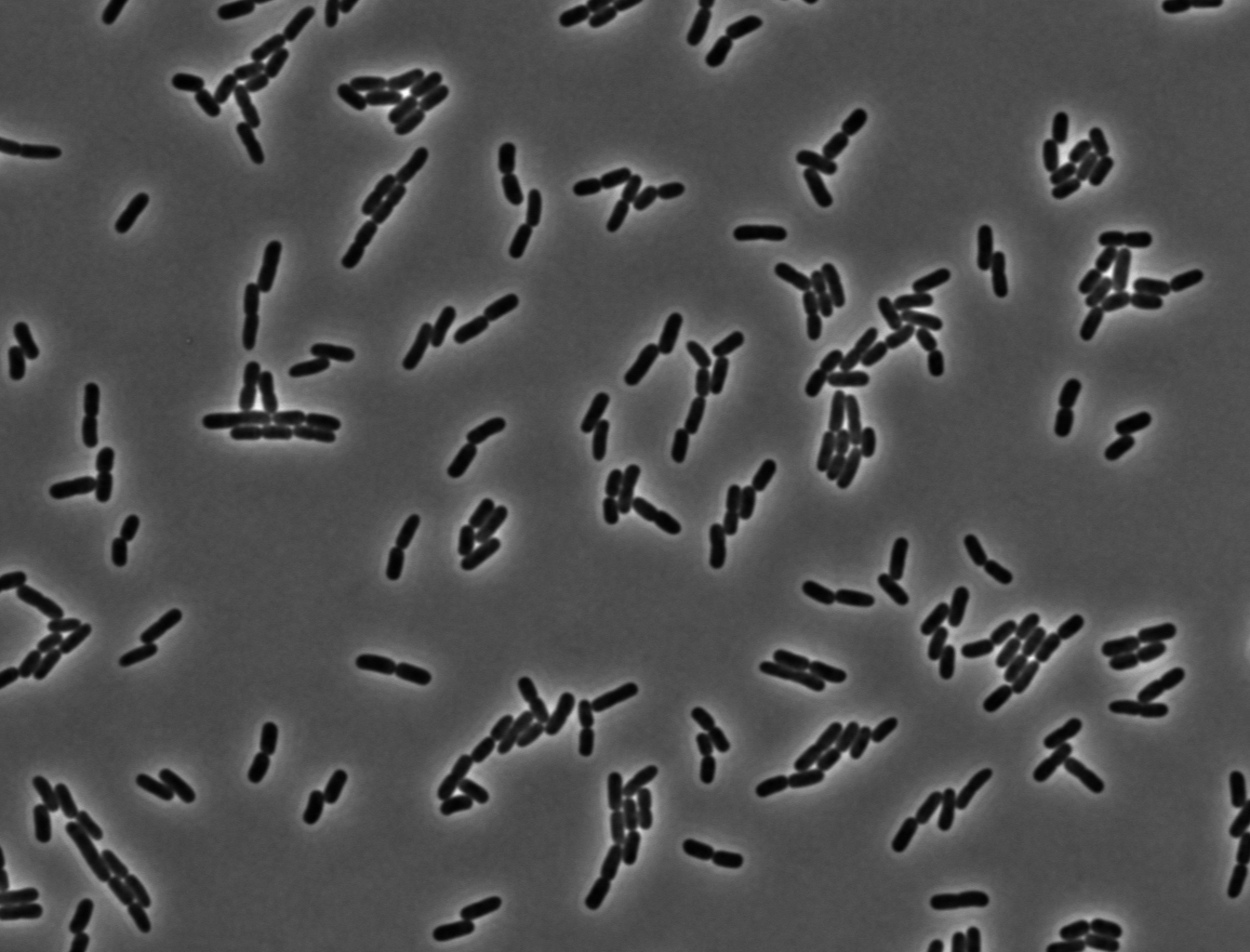

Supplement: Supplementary file 7 — Contains for each dataset the segmentation results of each method (.tif images) and corresponding parameterization files (.mat files). (ZIP 62299 kb) [file 12918_2017_399_MOESM7_ESM.zip › additional file 8/MT image/MicrobeTracker_TL1_2min_2hrs_St1_uint16-p-028.tif]

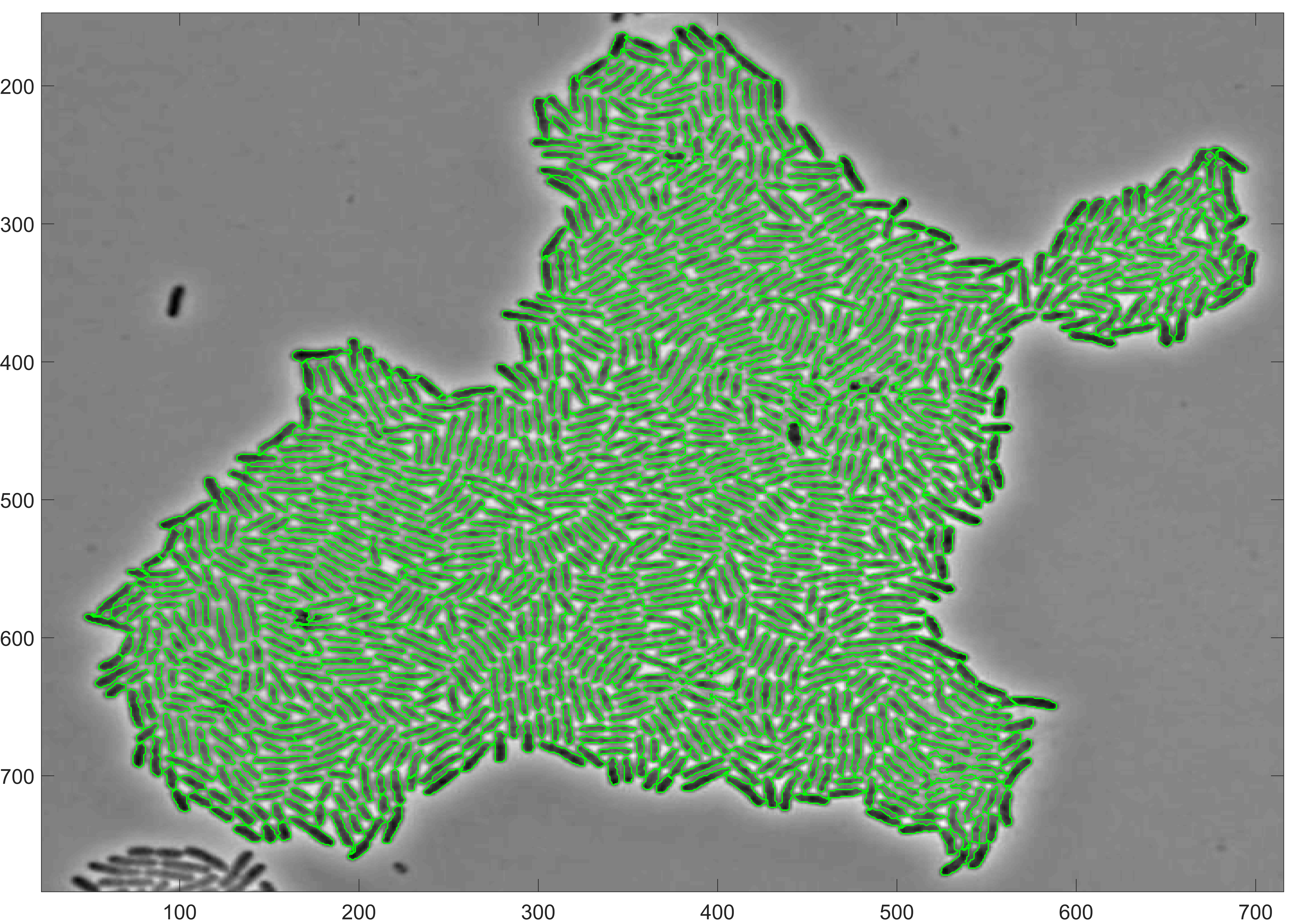

Supplement: Supplementary file 7 — Contains for each dataset the segmentation results of each method (.tif images) and corresponding parameterization files (.mat files). (ZIP 62299 kb) [file 12918_2017_399_MOESM7_ESM.zip › additional file 8/Multi-SalPhase image/sal15_1 078_BaSCA.tif]

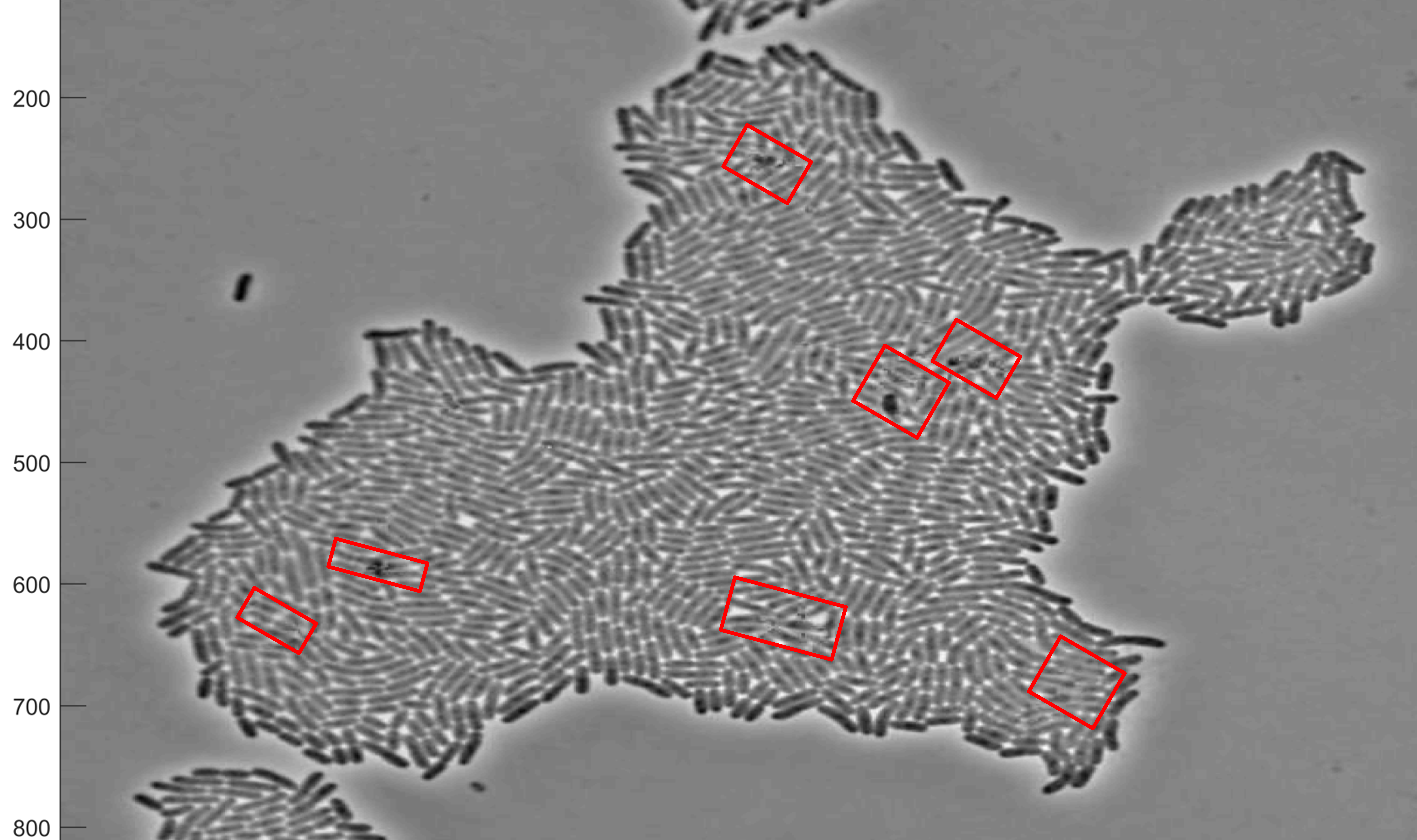

Supplement: Supplementary file 7 — Contains for each dataset the segmentation results of each method (.tif images) and corresponding parameterization files (.mat files). (ZIP 62299 kb) [file 12918_2017_399_MOESM7_ESM.zip › additional file 8/Multi-SalPhase image/sal15_1 078_ignored_regions.tif]

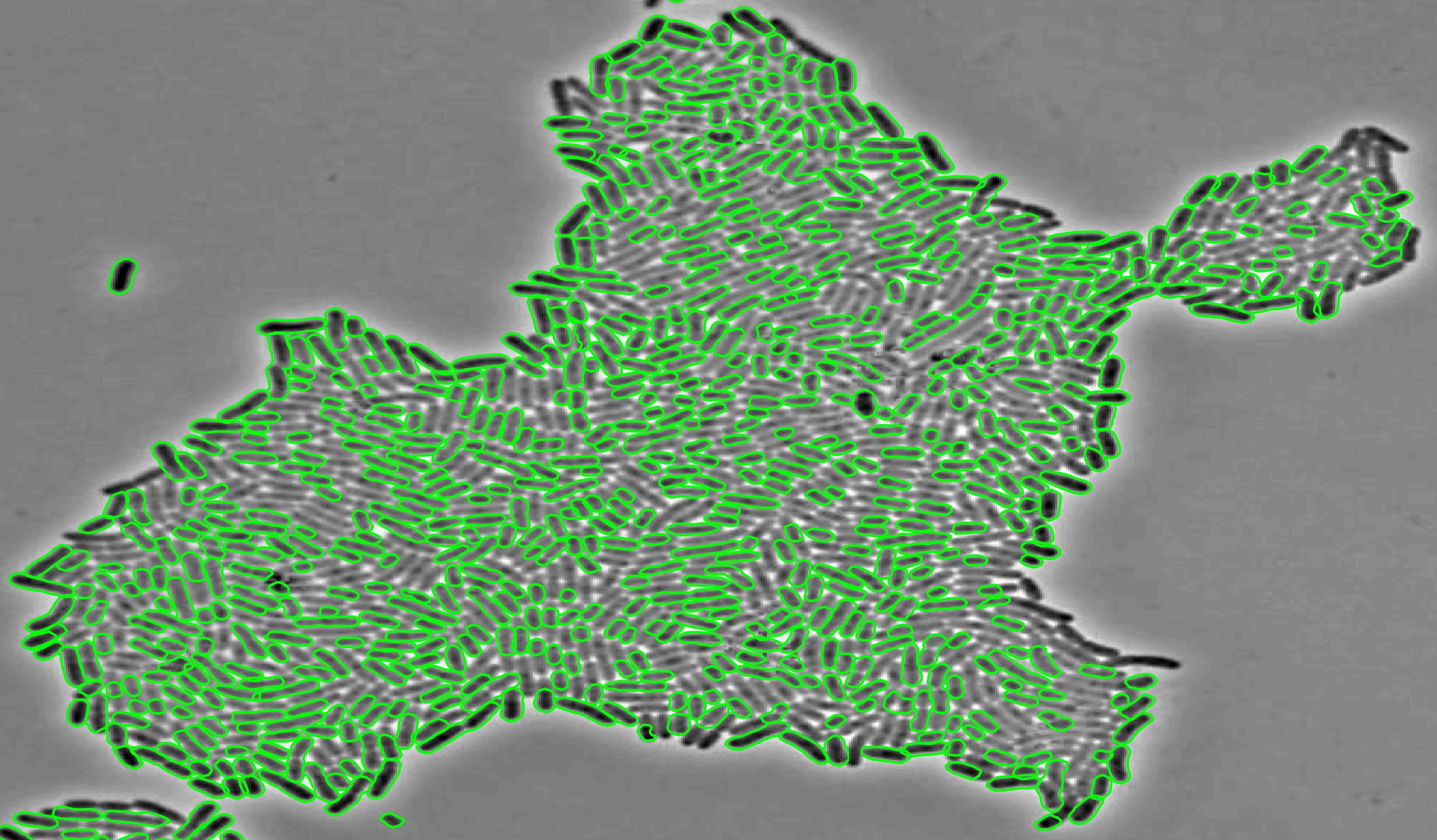

Supplement: Supplementary file 7 — Contains for each dataset the segmentation results of each method (.tif images) and corresponding parameterization files (.mat files). (ZIP 62299 kb) [file 12918_2017_399_MOESM7_ESM.zip › additional file 8/Multi-SalPhase image/sal15_1 078_Oufti.tif]

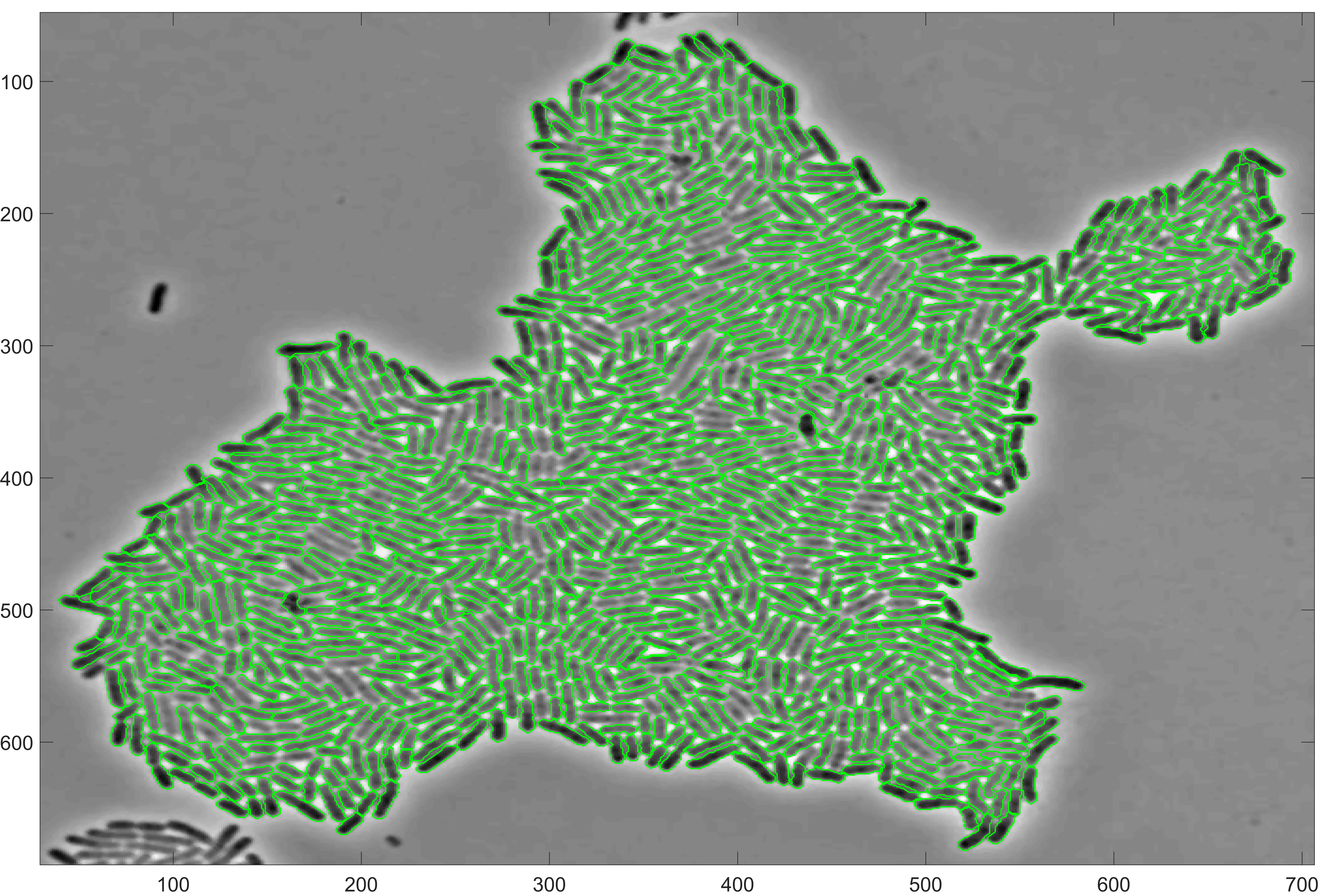

Supplement: Supplementary file 7 — Contains for each dataset the segmentation results of each method (.tif images) and corresponding parameterization files (.mat files). (ZIP 62299 kb) [file 12918_2017_399_MOESM7_ESM.zip › additional file 8/Multi-SalPhase image/sal15_1 078_Schnitzcells.tif]

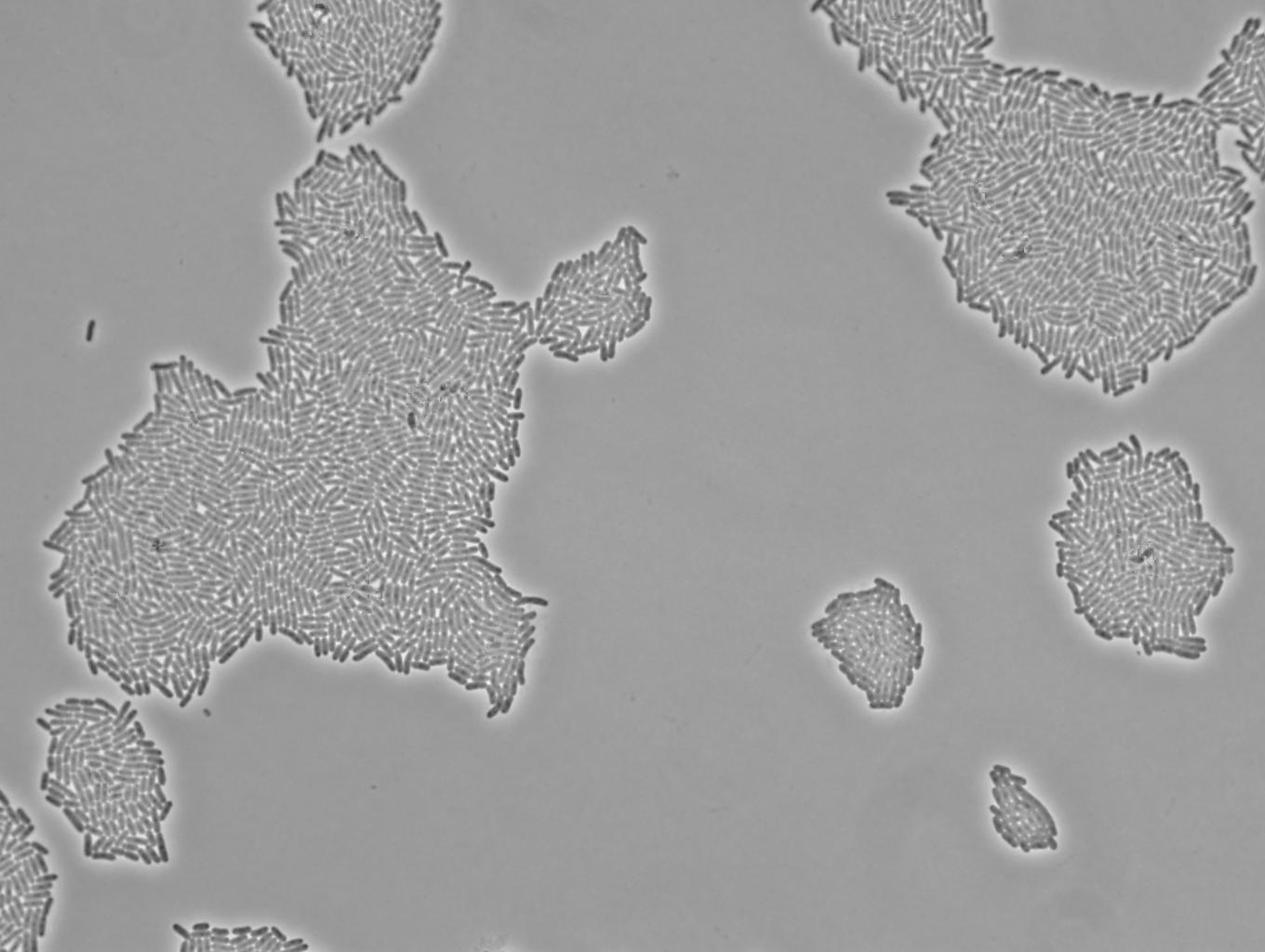

Supplement: Supplementary file 7 — Contains for each dataset the segmentation results of each method (.tif images) and corresponding parameterization files (.mat files). (ZIP 62299 kb) [file 12918_2017_399_MOESM7_ESM.zip › additional file 8/Multi-SalPhase image/sal15_1-p-078.tif]

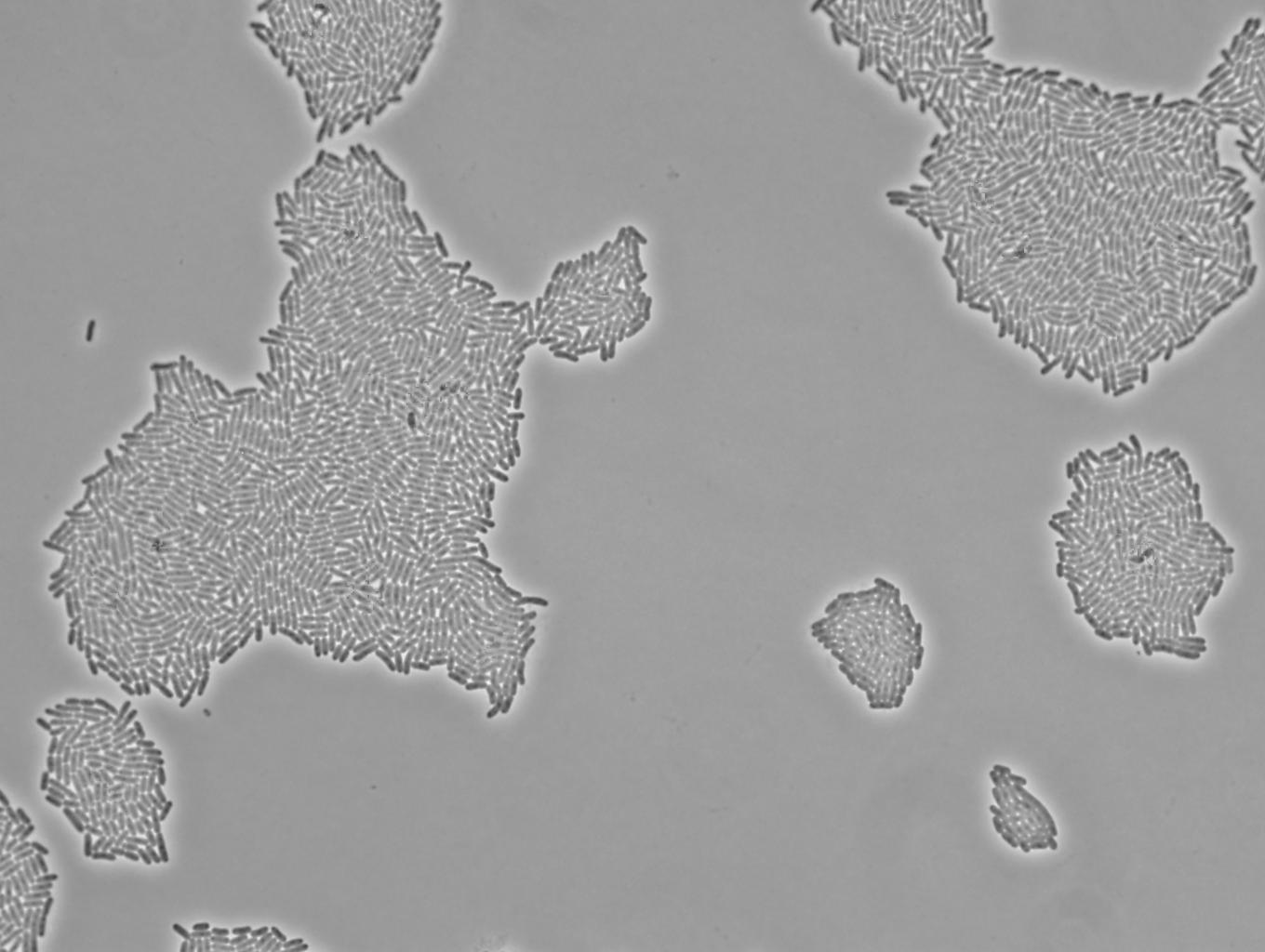

Supplement: Supplementary file 7 — Contains for each dataset the segmentation results of each method (.tif images) and corresponding parameterization files (.mat files). (ZIP 62299 kb) [file 12918_2017_399_MOESM7_ESM.zip › additional file 8/Multi-SalPhase image/sal15_1_uint16-p-078.tif]

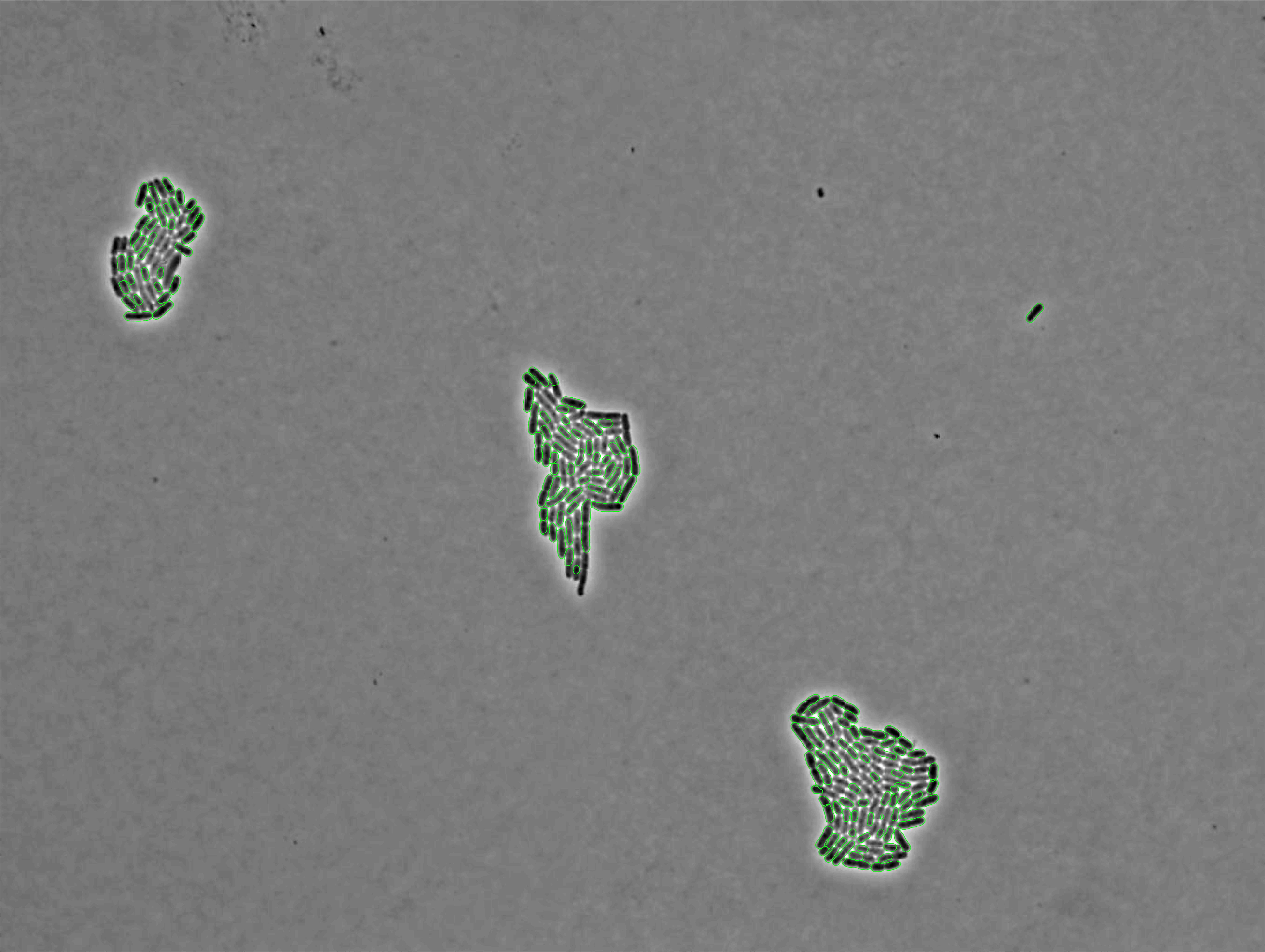

Supplement: Supplementary file 7 — Contains for each dataset the segmentation results of each method (.tif images) and corresponding parameterization files (.mat files). (ZIP 62299 kb) [file 12918_2017_399_MOESM7_ESM.zip › additional file 8/SalPhase image/sal15_9 074_Oufti.tif]

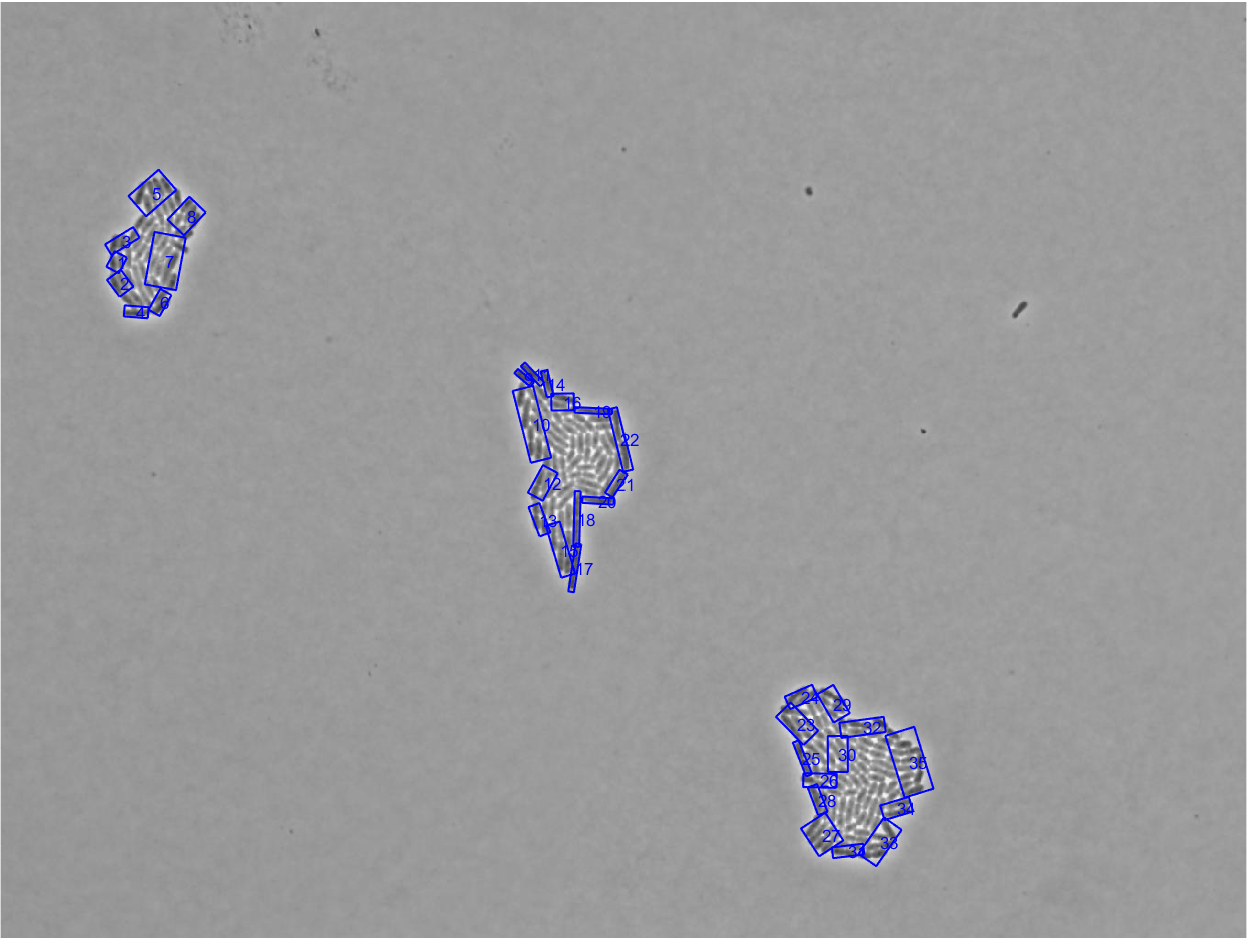

Supplement: Supplementary file 7 — Contains for each dataset the segmentation results of each method (.tif images) and corresponding parameterization files (.mat files). (ZIP 62299 kb) [file 12918_2017_399_MOESM7_ESM.zip › additional file 8/SalPhase image/sal15_9 074_TLM-Tracker.tiff]

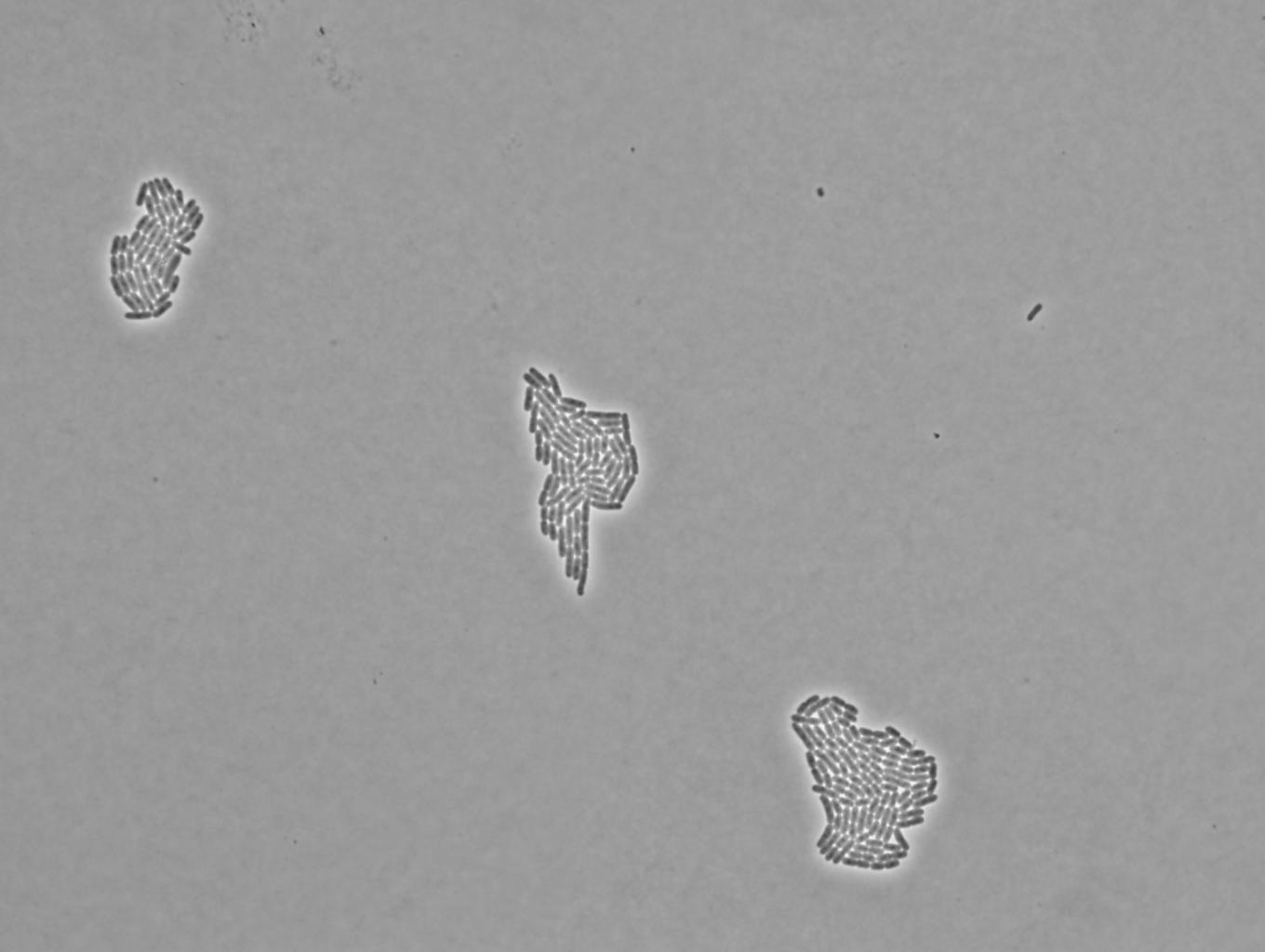

Supplement: Supplementary file 7 — Contains for each dataset the segmentation results of each method (.tif images) and corresponding parameterization files (.mat files). (ZIP 62299 kb) [file 12918_2017_399_MOESM7_ESM.zip › additional file 8/SalPhase image/sal15_9-p-074.tif]

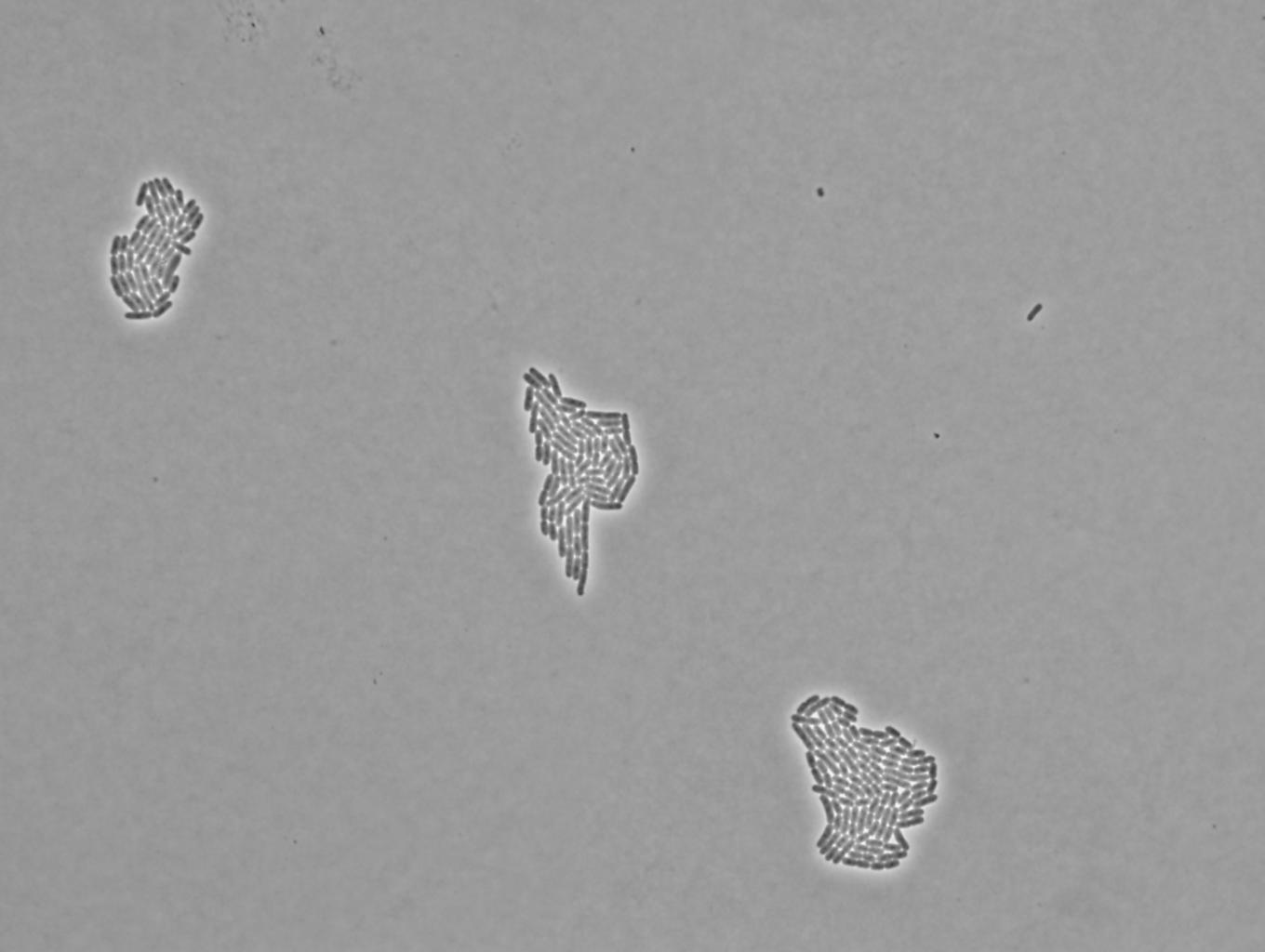

Supplement: Supplementary file 7 — Contains for each dataset the segmentation results of each method (.tif images) and corresponding parameterization files (.mat files). (ZIP 62299 kb) [file 12918_2017_399_MOESM7_ESM.zip › additional file 8/SalPhase image/sal15_9_uint16-p-074.tif]

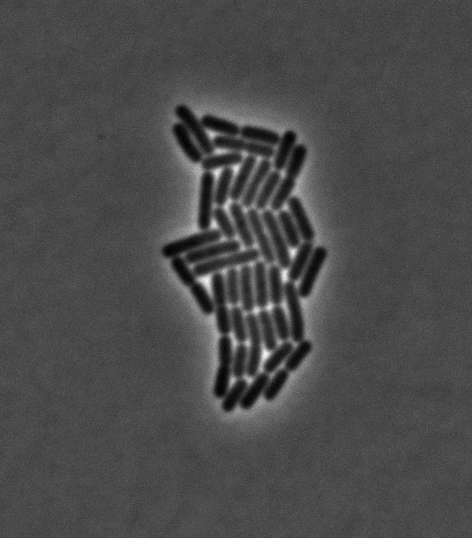

Supplement: Supplementary file 7 — Contains for each dataset the segmentation results of each method (.tif images) and corresponding parameterization files (.mat files). (ZIP 62299 kb) [file 12918_2017_399_MOESM7_ESM.zip › additional file 8/Schnitzcells image/Schnitzcells_TestSchnitz-01-p-020.tif]

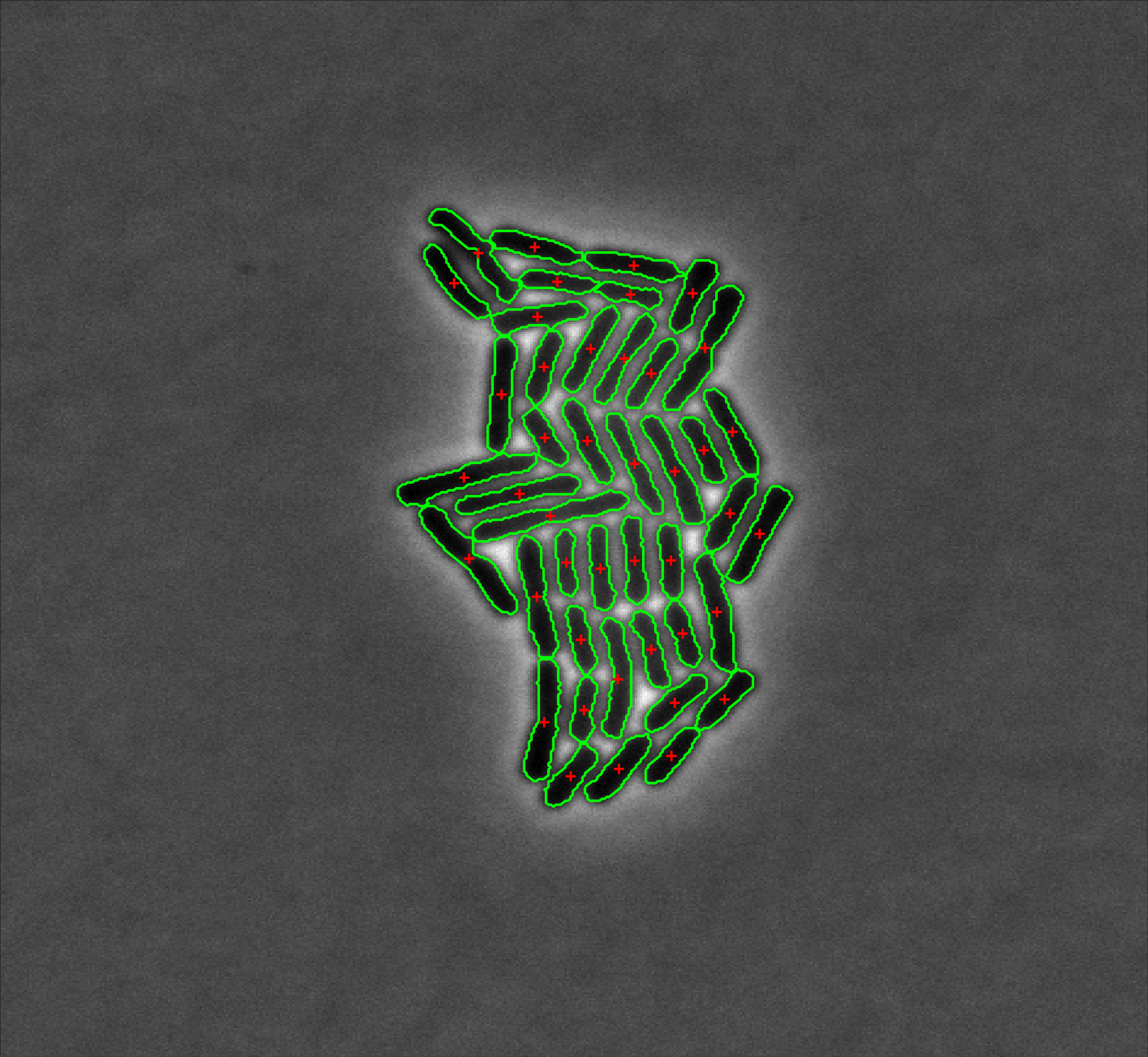

Supplement: Supplementary file 7 — Contains for each dataset the segmentation results of each method (.tif images) and corresponding parameterization files (.mat files). (ZIP 62299 kb) [file 12918_2017_399_MOESM7_ESM.zip › additional file 8/Schnitzcells image/Schnitzcells_TestSchnitz-01-p-020_BaSCA.tif]

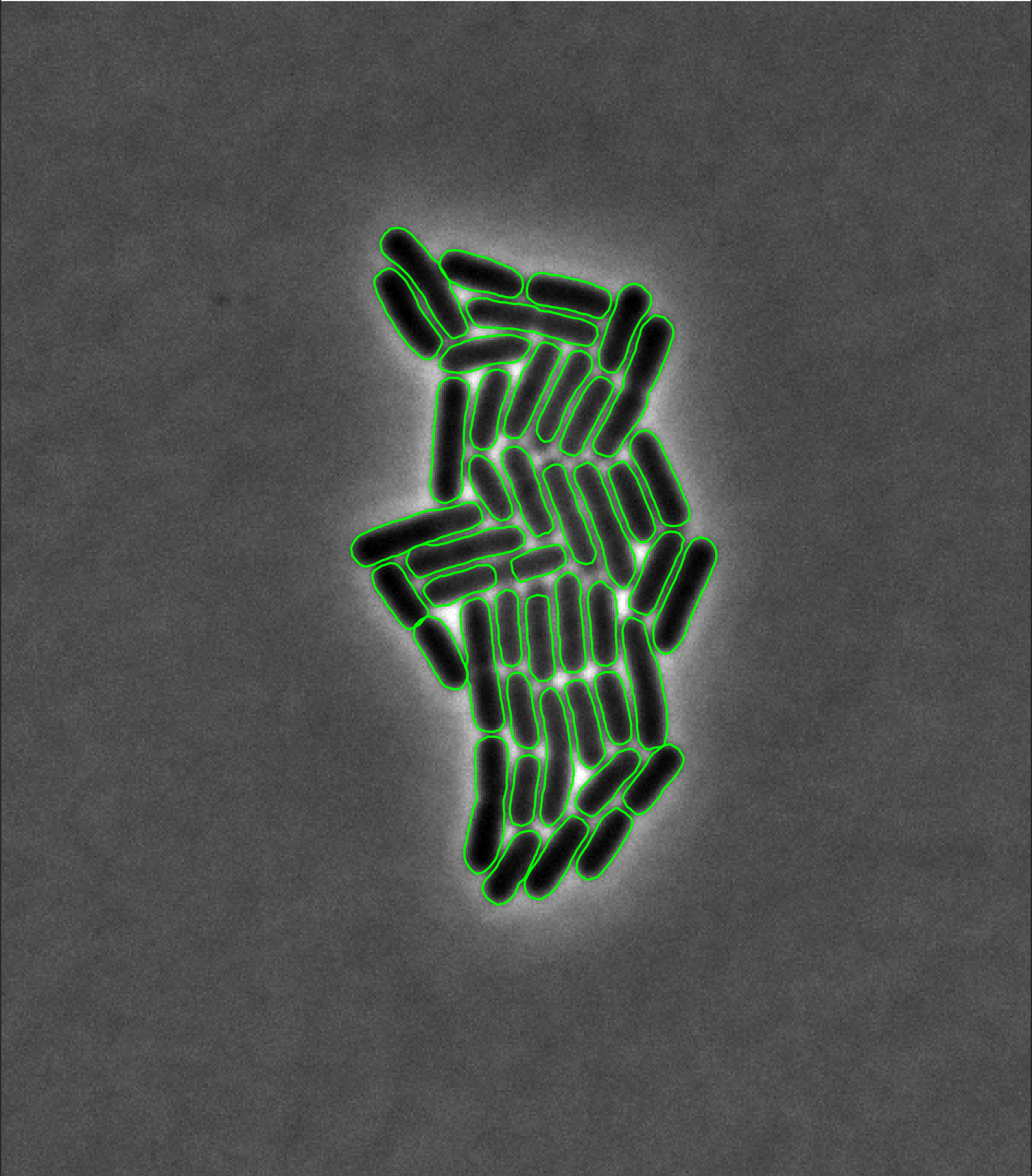

Supplement: Supplementary file 7 — Contains for each dataset the segmentation results of each method (.tif images) and corresponding parameterization files (.mat files). (ZIP 62299 kb) [file 12918_2017_399_MOESM7_ESM.zip › additional file 8/Schnitzcells image/Schnitzcells_TestSchnitz-01-p-020_Oufti.tif]

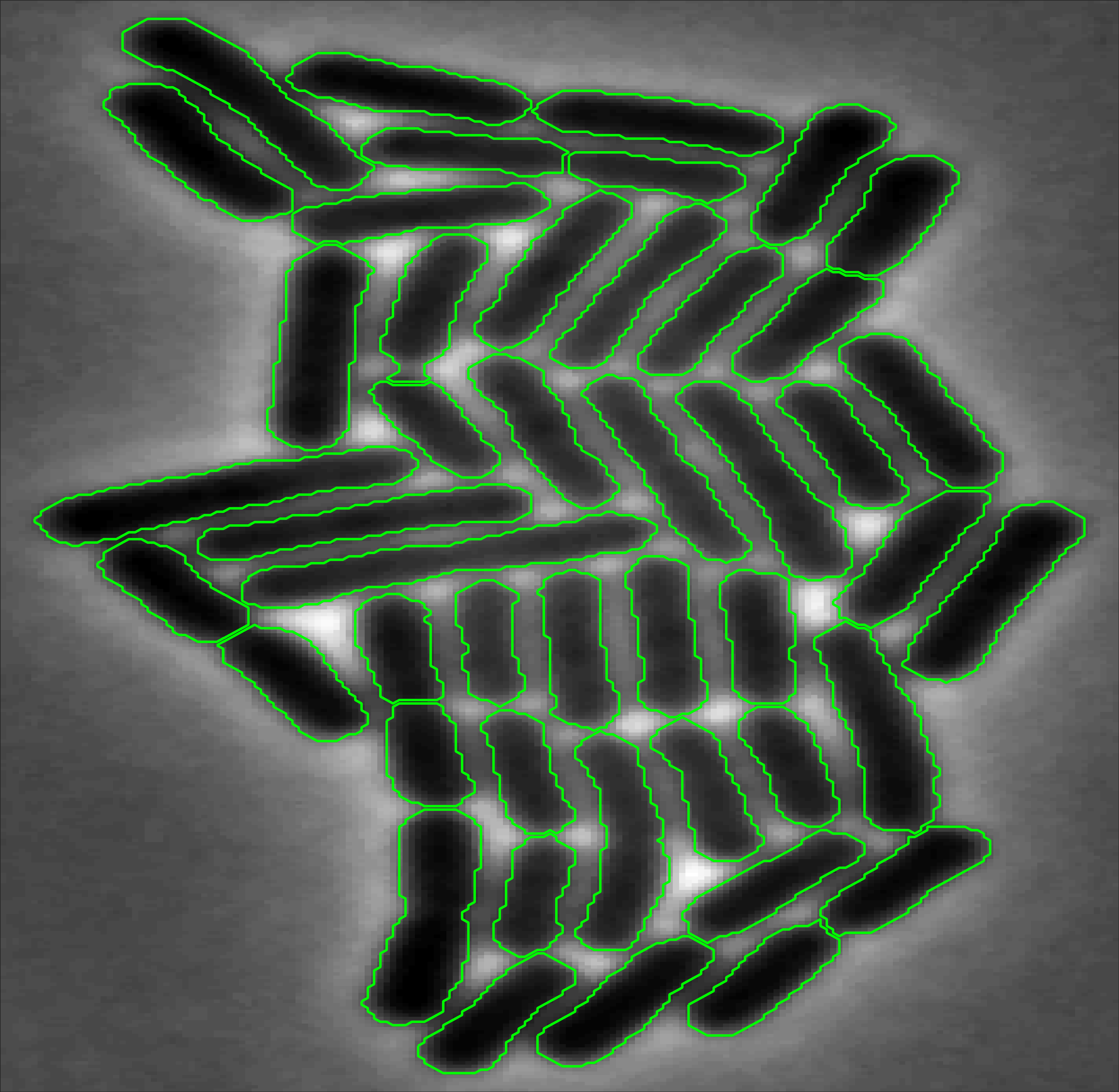

Supplement: Supplementary file 7 — Contains for each dataset the segmentation results of each method (.tif images) and corresponding parameterization files (.mat files). (ZIP 62299 kb) [file 12918_2017_399_MOESM7_ESM.zip › additional file 8/Schnitzcells image/Schnitzcells_TestSchnitz-01-p-020_Schnitzcells.tif]

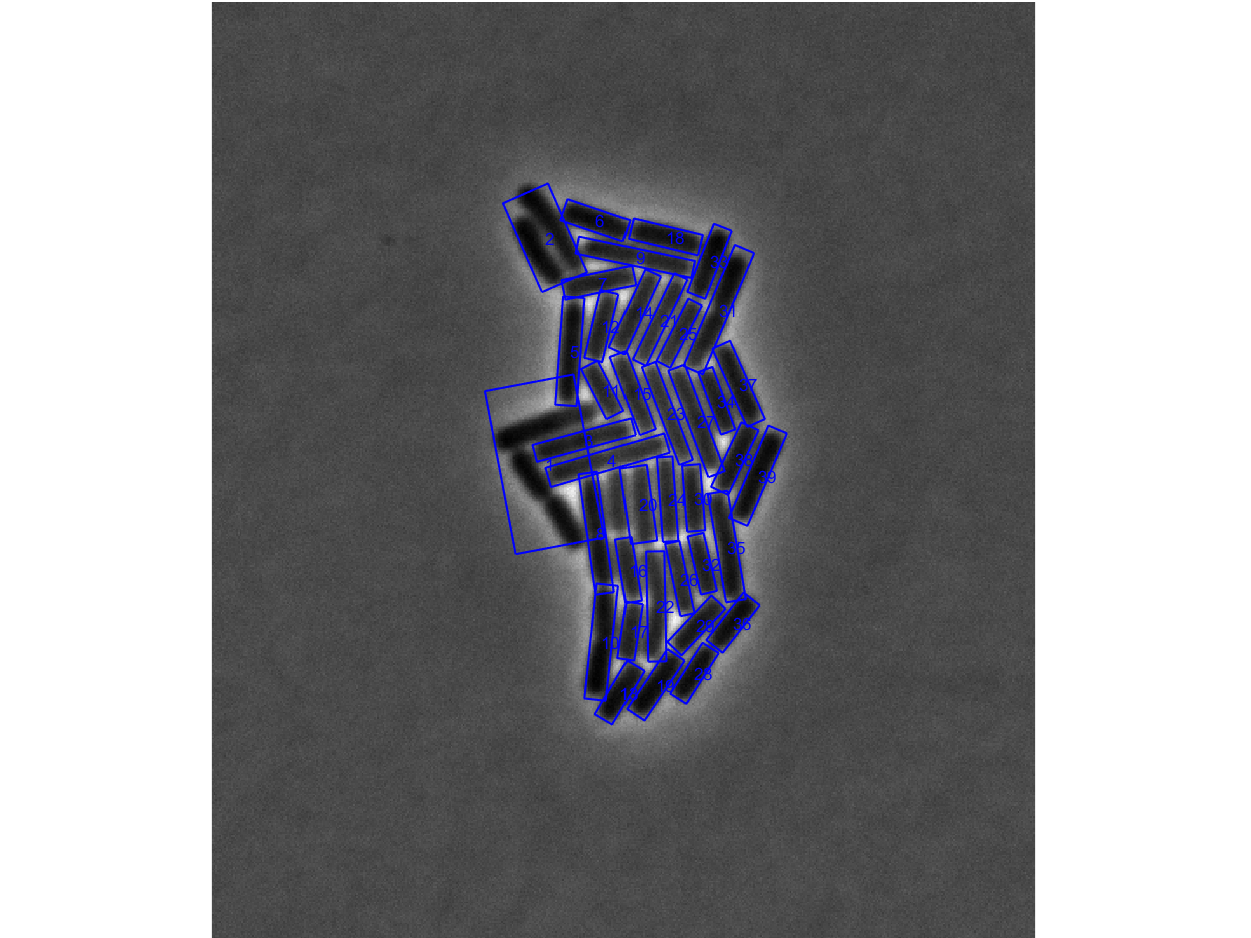

Supplement: Supplementary file 7 — Contains for each dataset the segmentation results of each method (.tif images) and corresponding parameterization files (.mat files). (ZIP 62299 kb) [file 12918_2017_399_MOESM7_ESM.zip › additional file 8/Schnitzcells image/Schnitzcells_TestSchnitz-01-p-020_TLM-Tracker.tiff]

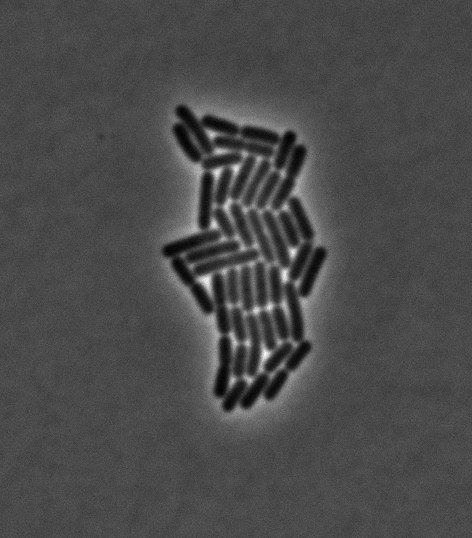

Supplement: Supplementary file 7 — Contains for each dataset the segmentation results of each method (.tif images) and corresponding parameterization files (.mat files). (ZIP 62299 kb) [file 12918_2017_399_MOESM7_ESM.zip › additional file 8/Schnitzcells image/Schnitzcells_TestSchnitz-01_uint16-p-020.tif]

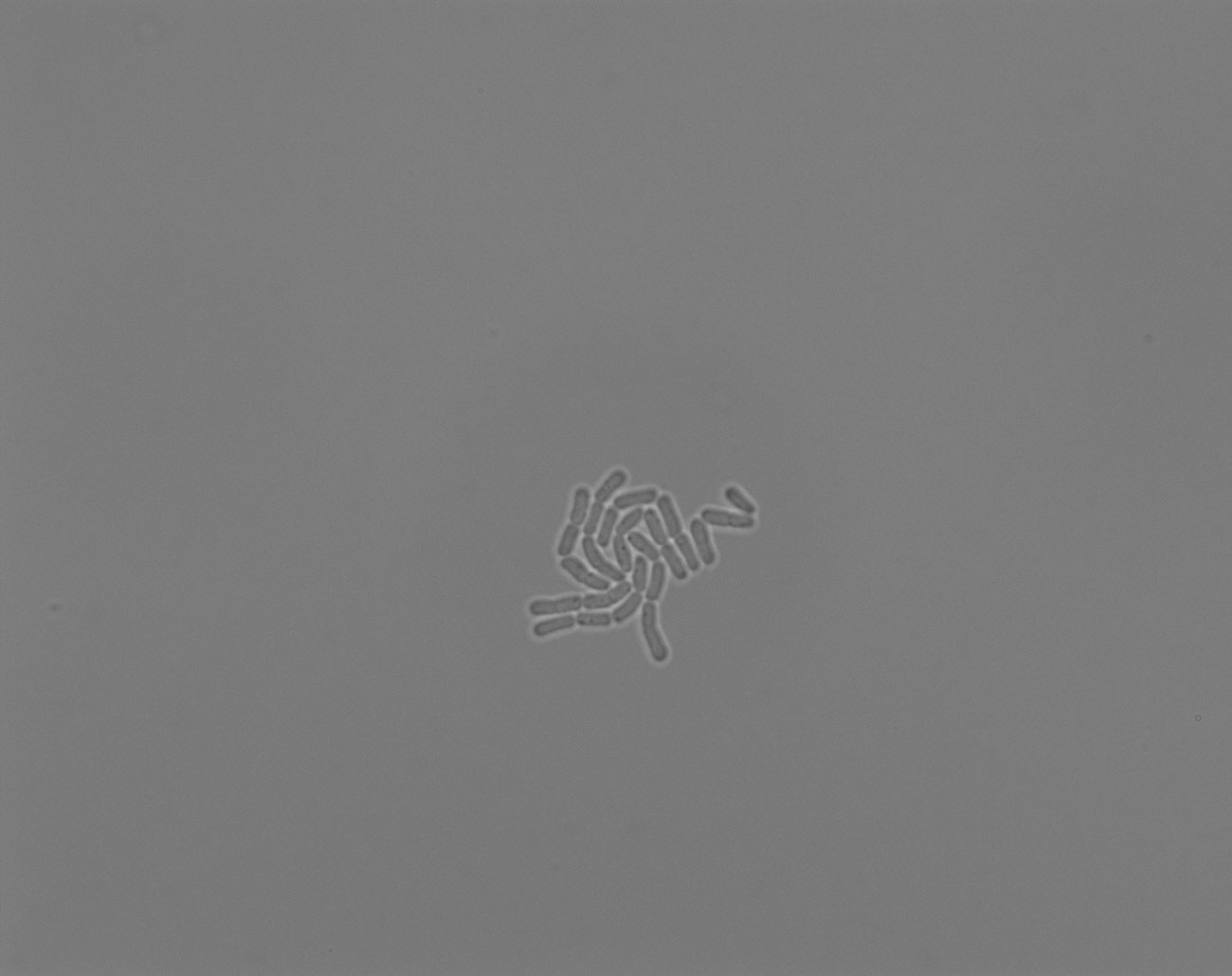

Supplement: Supplementary file 7 — Contains for each dataset the segmentation results of each method (.tif images) and corresponding parameterization files (.mat files). (ZIP 62299 kb) [file 12918_2017_399_MOESM7_ESM.zip › additional file 8/TLM-Tracker image/TLM-tracker_image045.tif]

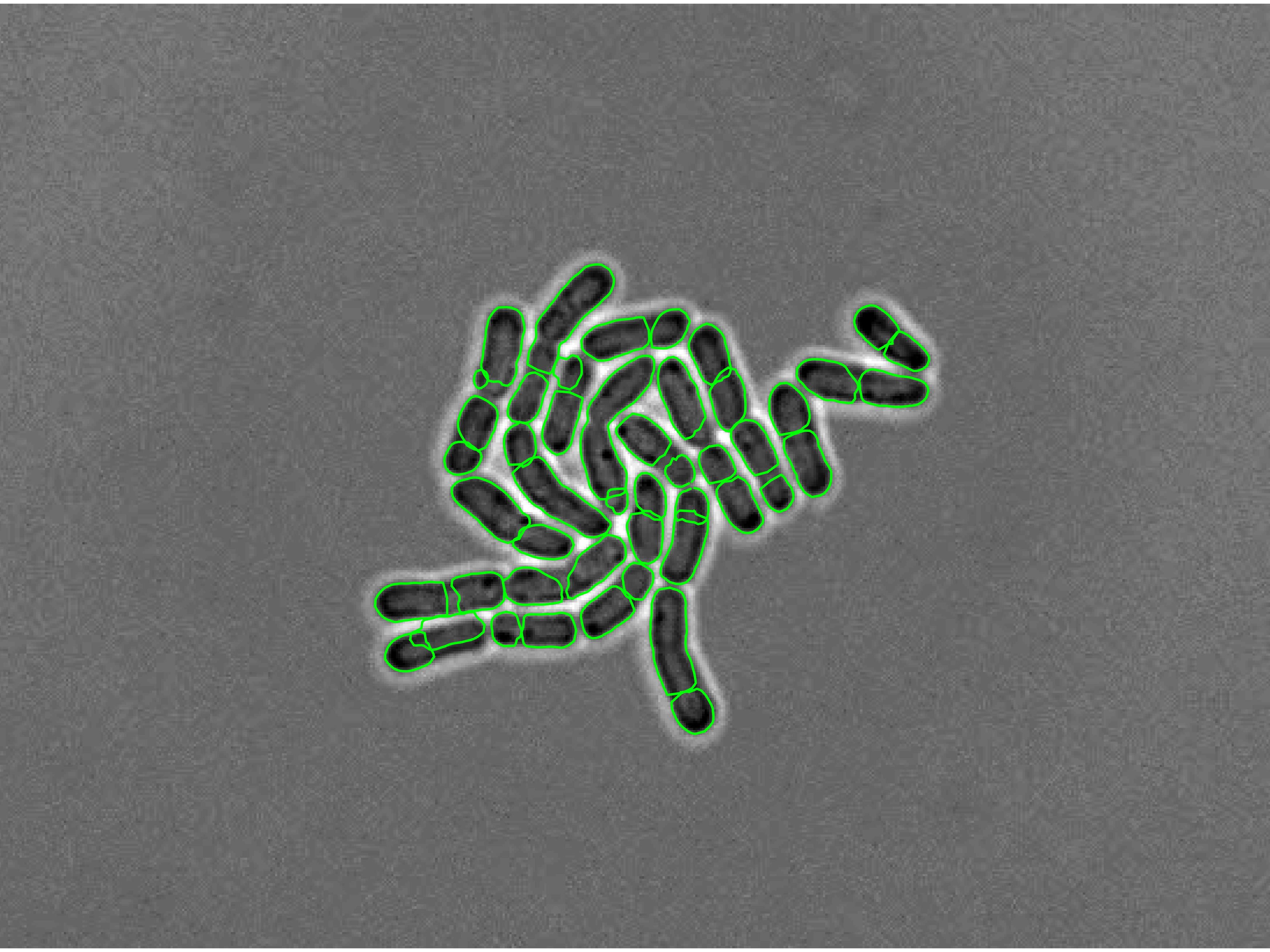

Supplement: Supplementary file 7 — Contains for each dataset the segmentation results of each method (.tif images) and corresponding parameterization files (.mat files). (ZIP 62299 kb) [file 12918_2017_399_MOESM7_ESM.zip › additional file 8/TLM-Tracker image/TLM-tracker_image045_Oufti.tif]

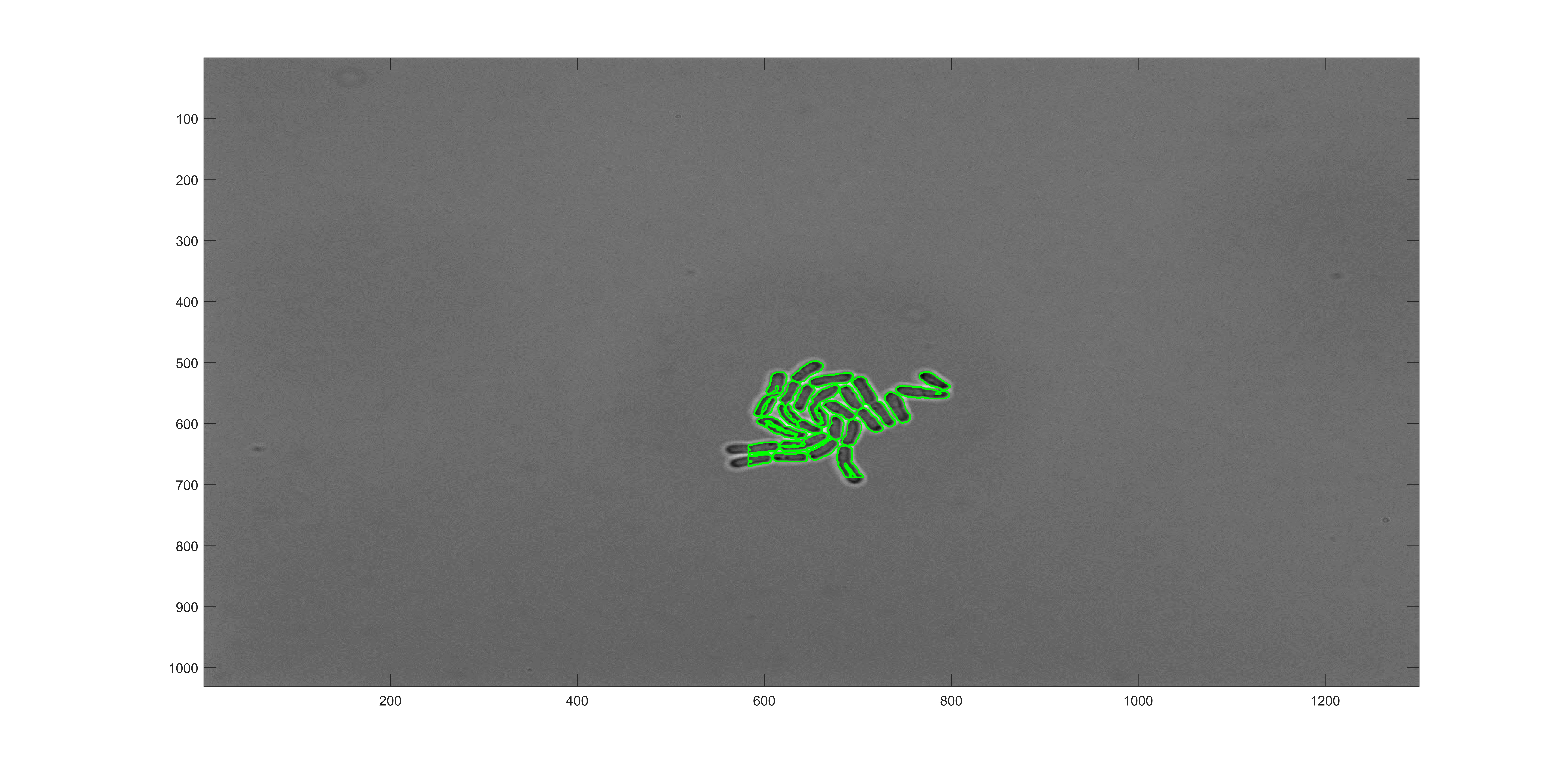

Supplement: Supplementary file 7 — Contains for each dataset the segmentation results of each method (.tif images) and corresponding parameterization files (.mat files). (ZIP 62299 kb) [file 12918_2017_399_MOESM7_ESM.zip › additional file 8/TLM-Tracker image/TLM-tracker_image045_Schnitzcells.tif]

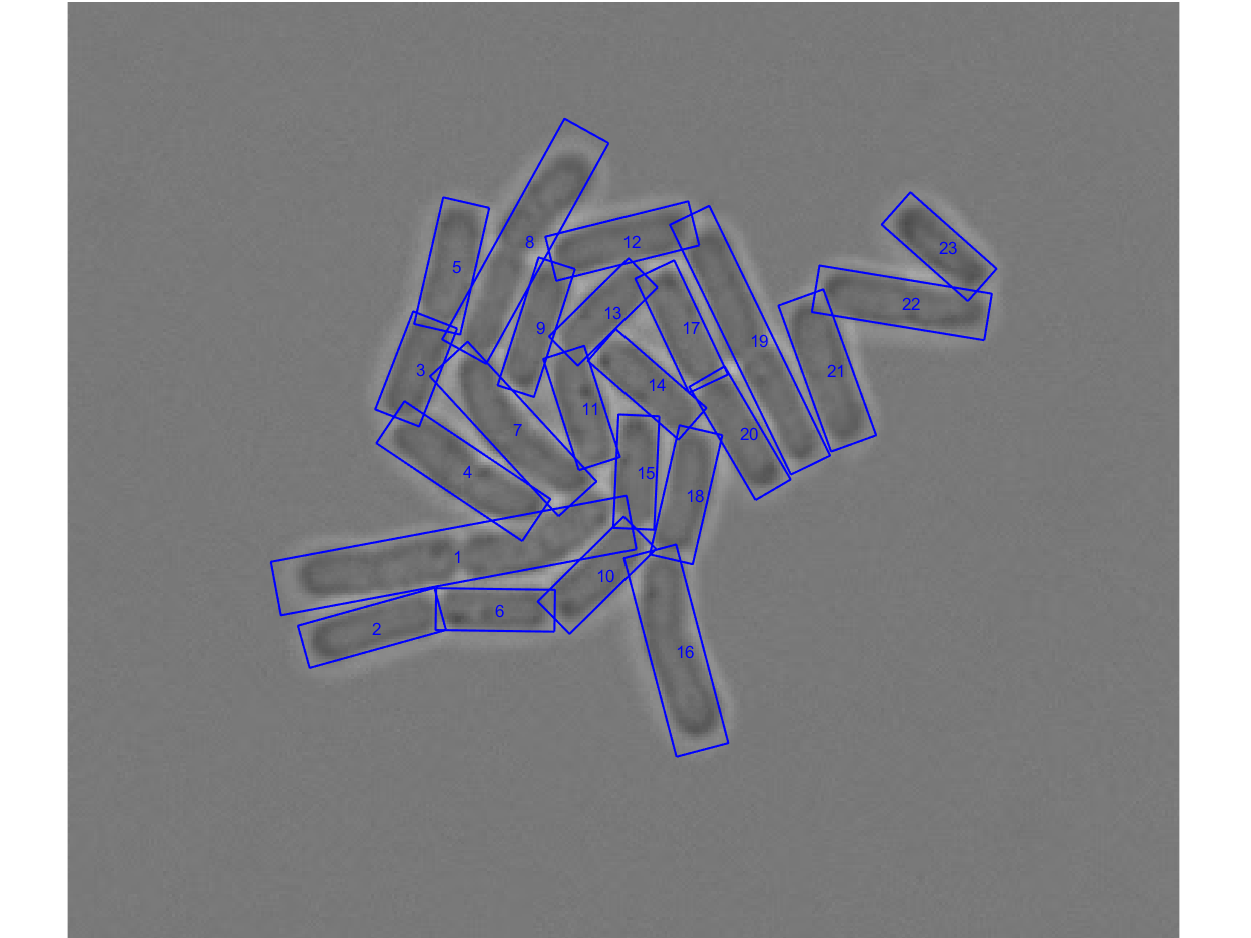

Supplement: Supplementary file 7 — Contains for each dataset the segmentation results of each method (.tif images) and corresponding parameterization files (.mat files). (ZIP 62299 kb) [file 12918_2017_399_MOESM7_ESM.zip › additional file 8/TLM-Tracker image/TLM-tracker_image045_tlm.tiff]

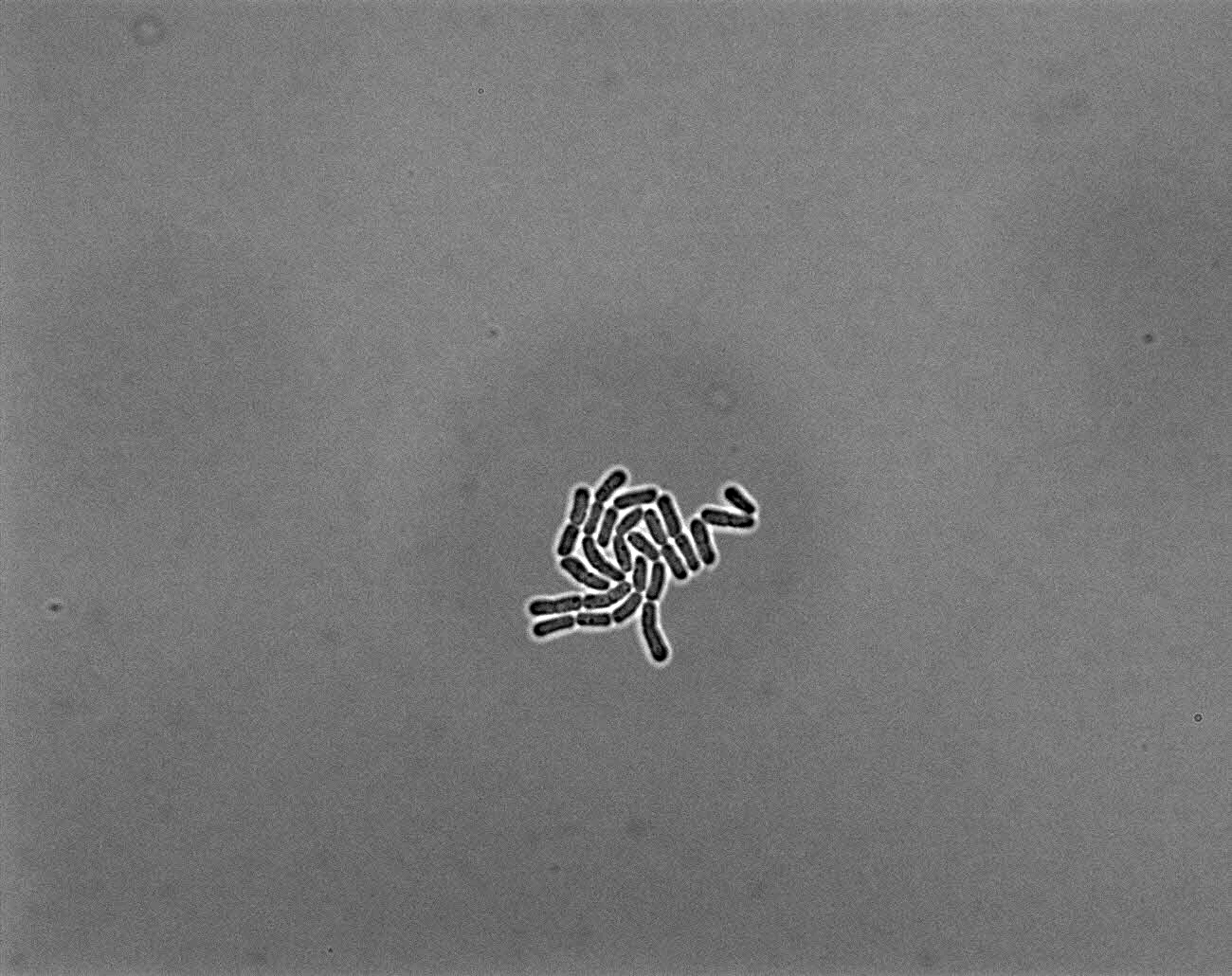

Supplement: Supplementary file 7 — Contains for each dataset the segmentation results of each method (.tif images) and corresponding parameterization files (.mat files). (ZIP 62299 kb) [file 12918_2017_399_MOESM7_ESM.zip › additional file 8/TLM-Tracker image/TLM-tracker_image_uint16-p-045.tif]
